# Oligogenic analysis across broad phenotypes of 46,XY differences in sex development associated with *NR5A1*/SF-1 variants: findings from the international SF1next study

**DOI:** 10.1016/j.ebiom.2025.105624

**Published:** 2025-03-03

**Authors:** Chrysanthi Kouri, Idoia Martinez de Lapiscina, Rawda Naamneh-Elzenaty, Grit Sommer, Kay-Sara Sauter, Christa E. Flück, SF1next study group, Saygin Abali, Zehra Yavas Abali, S. Faisal Ahmed, Leyla Akin, Maricruz Almaraz, Laura Audí, Murat Aydin, Antonio Balsamo, Federico Baronio, Jillian Bryce, Kanetee Busiah, Maria Caimari, Núria Camats-Tarruella, Ariadna Campos-Martorell, Luis Castaño, Anna Casteràs, Semra Çetinkaya, Hedi L. Claahsen - van der Grinten, Martine Cools, Ines Costa, Fatma Feyza Darendeliler, Justin H. Davies, Isabel Esteva, Helena Fabbri-Scallet, Courtney A. Finlayson, Emilio Garcia, Beatriz Garcia- Cuartero, Alina German, Evgenia Globa, Gil Guerra-Junior, Julio Guerrero, Tulay Guran, Sabine E. Hannema, Olaf Hiort, Josephine Hirsch, Ieuan Hughes, Marco Janner, Uchenna Kennedy, Zofia Kolesinska, Katherine Lachlan, Anna Lauber-Biason, Jana Krenek Malikova, Dagmar L’Allemand, Nina Lenhnerr-Taube, Angela Lucas-Herald, Jamala Mammadova, Veronica Mericq, Isabel Mönig, Francisca Moreno, Julia Mührer, Marek Niedziela, Anna Nordenstrom, Burçe Orman, Sukran Poyrazoglu, Jose M. Rial, Meilan M. Rutter, Amaia Rodríguez, Tara Schafer-Kalkhoff, Sumudu Nimali Seneviratne, Maria Sredkova-Ruskova, LIoyd J.W. Tack, Rieko Tadokoro-Cuccaro, Ajay Thankamony, Mónica Tomé, Amaia Vela, Malgorzata Wasniewska, David Zangen, Nataliya Zelinska

**Affiliations:** a Pediatric Endocrinology, Diabetology and Metabolism, Department of Pediatrics, Inselspital, Bern University Hospital, University of Bern, Bern 3010, Switzerland; b Department for BioMedical Research, University of Bern, Bern 3008, Switzerland; c Graduate School for Cellular and Biomedical Sciences, University of Bern, Bern 3012, Switzerland; d Research into the Genetics and Control of Diabetes and Other Endocrine Disorders, Biobizkaia Health Research Institute, Cruces University Hospital, Barakaldo 48903, Spain; e CIBER de Diabetes y Enfermedades Metabólicas Asociadas (CIBERDEM), Instituto de Salud Carlos III, Madrid 28029, Spain; f CIBER de Enfermedades Raras (CIBERER), Instituto de Salud Carlos III, Madrid 28029, Spain; g Endo-ERN, Amsterdam 1081 HV, the Netherlands; h Swiss Childhood Cancer Registry, Institute of Social and Preventive Medicine, University of Bern, Bern 3012, Switzerland

**Keywords:** Differences of sex development (DSD), Steroidogenic factor 1 (SF-1/*NR5A1*), 46,XY DSD, Oligogenicity

## Abstract

**Background:**

Oligogenic inheritance has been suggested as a possible mechanism to explain the broad phenotype observed in individuals with differences of sex development (DSD) harbouring *NR5A1*/SF-1 variants.

**Methods:**

We investigated genetic patterns of possible oligogenicity in a cohort of 30 individuals with *NR5A1*/SF-1 variants and 46,XY DSD recruited from the international SF1next study, using whole exome sequencing (WES) on family trios whenever available. WES data were analysed using a tailored filtering algorithm designed to identify rare variants in DSD and SF-1-related genes. Identified variants were subsequently tested using the Oligogenic Resource for Variant Analysis (ORVAL) bioinformatics platform for a possible combined pathogenicity with the individual *NR5A1*/SF-1 variant.

**Findings:**

In 73% (22/30) of the individuals with *NR5A1*/SF-1 related 46,XY DSD, we identified one to seven additional variants, predominantly in known DSD-related genes, that might contribute to the phenotype. We found identical variants in eight unrelated individuals with DSD in DSD-related genes (e.g., *TBCE, FLNB, GLI3* and *PDGFRA*) and different variants in eight genes frequently associated with DSD (e.g., *CDH23, FLNB, GLI2, KAT6B, MYO7A, PKD1, SPRY4* and *ZFPM2*) in 15 index cases. Our study also identified combinations with *NR5A1*/SF-1 variants and variants in novel candidate genes.

**Interpretation:**

These findings highlight the complex genetic landscape of DSD associated with *NR5A1*/SF-1, where in several cases, the use of advanced genetic testing and filtering with specific algorithms and machine learning tools revealed additional genetic hits that may contribute to the phenotype.

**Funding:**

10.13039/501100001711Swiss National Science Foundation and Boveri Foundation Zurich.

**Research in context**

**Evidence before this study**

Steroidogenic Factor 1/Nuclear Receptor Subfamily 5 Group A Member 1 (SF-1/*NR5A1*) is essential for human sex development and steroidogenesis. Variants in the *NR5A1*/SF-1 gene are associated with broad phenotypes, ranging from severe to mild differences of sex development (DSD), and isolated fertility problems to complete lack of symptoms. Previous research has proposed various mechanisms for explaining this phenotypic variability, but none were confirmed. Even the international SF1next study, which collected data from the thus far largest cohort of 197 individuals with *NR5A1*/SF-1 variants to date, could not find phenotype–genotype correlations. Still, the SF1next study revealed a higher risk for associated anomalies in *NRA5A1*/SF-1 variant carriers, particularly related to the spleen as confirmed by subsequent studies. Moreover, individuals with a *NR5A1*/SF-1 variant and a severe DSD phenotype were reported to have atypical pubertal development and foreseen fertility issues. Reports using next generation sequencing (NGS) identified multiple additional gene variants in individuals with *NR5A1*/SF-1 variants and DSD, suggesting that genetic modifiers or oligogenic inheritance may explain the variability of phenotypes. However, proving oligogenicity is challenging, especially in rare diseases like DSD with limited statistical power and a small pool of individuals available for comprehensive genetic evaluation and segregation analysis.

**Added value of this study**

This study builds on the SF1next international study cohort, offering valuable insights into the complex genetic landscape of individuals with *NR5A1*/SF-1 variants and DSD. It investigates possible oligogenicity through whole exome sequencing (WES), employing a tailored filtering algorithm for detection of rare variants in DSD and SF-1 related genes, followed by bioinformatic prediction analysis for oligogenicity, using ORVAL. Investigating 30 index cases with *NR5A1*/SF-1 variants and 35 family members, possible oligogenic inheritance was found in 73% of index cases, each harbouring one to seven additional variants, predominantly in known DSD-related genes. Common variants were observed in unrelated individuals, and certain DSD-related genes were detected more frequently, though with different variants, while variants in novel genes were also found. These studies together with segregation analysis in several cases, inform that oligogenicity may contribute to the observed phenotypic variability in DSD associated with *NR5A1*/SF-1 variants.

**Implications of all the available evidence**

The identification of possible additional variants in DSD/SF-1- related genes in individuals with *NR5A1*/SF-1 variants and DSD, suggests that the phenotypic variability observed may instead result from the interplay of multiple genetic variants than from a single variant in *NR5A1*/SF-1 alone. This highlights the necessity for advanced genetic testing and bioinformatic analysis with disease-tailored algorithms to capture the full spectrum of genetic variants contributing to DSD and their potential interactions. The genetic basis of DSD linked with *NR5A1*/SF-1 variants might be more complex than initially thought, reinforcing the need for further research addressing the role of additional genetic contributors.

## Introduction

Differences of sex development (DSD; also known as disorders of sex development) represent a heterogenous group of rare congenital conditions affecting the chromosomal, gonadal or anatomical sex.^1^ These conditions may become obvious at different ages of life. Some fetuses or newborns may manifest with ambiguous (atypical) external genitalia early, while dysgenetic gonads, and discordant internal sex organs relative to the sex chromosome composition may be discovered later. A DSD diagnosis may also be made later in life because of missing, delayed and/or atypical pubertal development including absence of menarche, unexpected virilisation and/or gynaecomastia, as well as infertility or the occurrence of a gonadal tumour.^2^

The wide spectrum of phenotypes and underlying genotypes observed in individuals with DSD provides a diagnostic challenge. The DSD phenotype may vary between individuals, even carrying the same variant within a family. Additionally, about 20–30% of individuals with DSD have other organ anomalies or associated medical conditions that may lead to misdiagnosis or delayed diagnosis.^2^, ^3^, ^4^

Although more than hundred genes have been implicated in DSD, half of patients with DSD still have no definite molecular diagnosis with the currently used routine diagnostic methods.^5^ Known genetic causes of DSD include chromosomal aneuploidies (e.g. Turner syndrome, Klinefelter syndrome), large rearrangements, small copy number variants (CNVs) of open reading frames or promoter regions, and specific variants in single genes.^6^ The testing methods primarily detect coding single nucleotide variants (SNVs) and CNVs. However, other types of variants and aetiologies have been identified as potential causes for DSD.^7^ These include variants located in intronic regions,^8^^,^^9^ present in mosaic states,^10^^,^^11^ structural^12^^,^^13^ and epigenetic variations,^14^ as well as oligogenic causation.^15^, ^16^, ^17^

Advances in next generation sequencing (NGS) technologies have enhanced the possibility to discover an oligogenic basis for several endocrine disorders such as hypogonadotropic hypogonadism, hypothyroidism and primary/premature ovarian insufficiency (POI).^18^, ^19^, ^20^, ^21^, ^22^, ^23^ Similarly, oligogenic inheritance patterns have been reported in several cases of DSD.^15^, ^16^, ^17^^,^^24^ The oligogenic mode of inheritance has been proposed as a potential explanation for the broad spectrum of phenotypes observed in individuals with *NR5A1*/SF-1 variants encompassing healthy individuals, individuals with mild to severe or opposite sex DSD, male infertility, POI, and adrenal insufficiency.^24^, ^25^, ^26^, ^27^, ^28^, ^29^ So far, other mechanisms explaining the genotype–phenotype correlation associated with *NR5A1*/SF-1 have not been confirmed, including dominant negative effects^27^^,^^30^, ^31^, ^32^, ^33^ and haploinsufficiency.^26^^,^^29^ To detect oligogenicity, identification of multiple, potentially disease-causing variants through NGS analysis is the first step. Further validation of the identified variants with bioinformatic and machine learning tools and functional testing^34^^,^^35^ is then required for assessing the possible combined effect of multiple variants associated with the disease phenotype. Functional testing is significantly more complex for confirming oligogenic disease mechanisms than it is for monogenic disorders.^36^ However, family-based genetic analysis may help uncovering the disease origin, where the analysis of family trios in particular can reveal variants that violate the rules of Mendelian inheritance patterns.

In this study, we therefore investigated a large group of individuals with heterozygous *NR5A1*/SF-1 variants and a broad range of DSD (recruited through the international SF1next study) for possible oligogenic disease mechanisms using WES and bioinformatic analysis and performing additional genetic analysis of their healthy and/or affected family members.

## Methods

### Participants

Patient and families participating in this study were recruited through the international SF1next study cohort, which comprises 197 individuals.^25^ In this study we included participants with available DNA samples, who consented for additional genetic testing with WES (Supplementary Fig. S1). DNA samples extracted from the blood of individuals carrying *NR5A1*/SF-1 variants, as well as from family members, were collected by the SF1next study collaborators (Supplementary Fig. S1). Data on comprehensive phenotyping of study participants were provided by the caring clinicians of the SF1next study, as previously described.^25^ The classification of the DSD phenotype was a modified version of the external genitalia score to accommodate for retrospective data collection, as described in detail in the SF1next study.^25^ All clinical data and DNA samples included in this study were collected in pseudo-anonymised form. The University of Glasgow has ethical approval and provides guidelines to international partner centres for collecting routine data of individuals with a DSD in the SDM Registries platform that includes I-DSD (https://sdmregistries.org/, UKCRN ID12729).

### Ethics

International clinical partners received approval from their respective ethical committees to participate in the study, and written informed consent was obtained from all study participants. For the Swiss study core centre in Bern ethical approval exists through the Swiss DSD Cohort Study (BASEC ID 2016-01210).

### Genetic analysis

WES was performed on 25 individuals/families by Novogene (UK). In five individuals/families, WES analysis was previously performed and data were reanalysed using the Variant Call Format (VCF) files as input for performing variant annotation by ANNOVAR.^37^ The filtration process of exonic variants was conducted with the R software (R.4.3.0). WES data were analysed using a tailored filtering algorithm designed to identify rare variants in SF-1- and DSD-related genes, as previously reported.^26^^,^^38^ All variants with any predicted consequences, except synonymous ones, and with a read depth of 20 or more were retained. Subsequently, variants were filtered further to include only those with MAF (Minor Allele Frequency) ≤ 0.01 based on gnomAD (v3.1.2), taking into consideration the karyotype of the patient. Annotation was verified using VarSome^39^ and Franklin^40^ platforms, followed by variant classification and in silico analysis.

### *In silico* analysis and variant classification

We utilised various in silico tools to predict the potential impact of identified genetic variants on both structure and function of the protein, including: Polyphen-2, (Polymorphism Phenotyping v2), Panther (Protein Analysis Through Evolutionary Relationships), SNPs and GO, CADD (Combined Annotation Dependent Depletion)^41^ and the calibrated scores given by VarSome^39^ for SIFT (Scale-invariant feature transform), Provean (Protein Variation Effect Analyser), Revel (Rare Exome Variant Ensemble Learner), Mutation taster, and M-CAP (Mendelian Clinically Applicable Pathogenicity). The variants were classified according to the standards and guidelines of the American college of Medical Genetic and Genomics (ACMG)^42^ using VarSome^39^ and Franklin^40^ platforms.

### Oligogenic investigations and selection of candidate variants

We considered filtered variants as candidates for oligogenicity in combination, with *NR5A1*/SF-1 variants based on two criteria, similar to published literature.^43^, ^44^, ^45^ For the first criterion, we utilised the Oligogenic Resource for Variant Analysis (ORVAL) bioinformatics platform^34^ to identify candidate oligogenic variant combinations associated with each individual’s *NR5A1*/SF-1 variant. We specifically tested the variants in each case, using ORVAL’s machine-learning tool, the Variant Combination Pathogenicity Predictor (VarCoPP). VarCoPP is a balanced random forest predictor that assesses the pathogenicity of variant combinations in gene pairs. It uses various biological features of genes, variants, and gene pairs to make the predictions.^46^^,^^47^ The output is a pathogenicity score (VarCoPP score) that indicates the probability (value between 0 and 1) that a variant combination is disease-causing. If this score is above 0.4575 (hg38), the model predicts that the combination is disease-causing. In our analysis, we set a threshold of ≥0.85 (hg38) for the pathogenicity score of candidate variant combinations, to include only those falling in the 99.9% confidence zone, which indicates a 99.9% probability of being true positives. In addition, we evaluated variants using ORVAL’s second machine-learning tool, the Digenic Effect Predictor.^48^ This tool predicts the type of variants combinations and categorise them into three classes: i. true digenic, ii. monogenic and modifier, and iii. dual molecular diagnosis.^47^^,^^49^ As a second criterion, we included variants classified as pathogenic, likely pathogenic or VUS according to the ACMG criteria or classified as pathogenic, likely pathogenic or VUS by at least seven out of nine prediction tools, regardless of a defined prediction in ORVAL (VarCoPP). For both criteria, previously reported clinical associations of variants were checked in ClinVar and HGMD databases. Additionally, extensive literature search (e.g., PubMed) was conducted to explore gene and variant associations with DSD, sex development, and the specific clinical phenotype for each individual case and related family. Variants lacking an association with the observed phenotype of the individuals (according to literature) were rejected. In addition, whenever possible, family segregation and trio analysis were conducted to better understand potential oligogenic inheritance. This approach included a comprehensive assessment of both genetic and phenotypic data between family members with *NR5A1*/SF-1 variants, to determine the possible contribution of individual variants to the observed phenotype. Furthermore, comparative analysis of the identified variants across the entire group of carriers of *NR5A1*/SF-1 variants was performed.

### Statistics

For this very rare disease, sample size estimation and power analysis for statistical calculations do not apply as the numbers are too low, and the analyses are therefore descriptive.

### Role of funders

None of the funding sources (Swiss National Science Foundation and Boveri Foundation Zurich), which supported this study, had any role in study design, data collection, analysis, interpretation, manuscript writing or decision for publication.

## Results

**Fig. 1:**
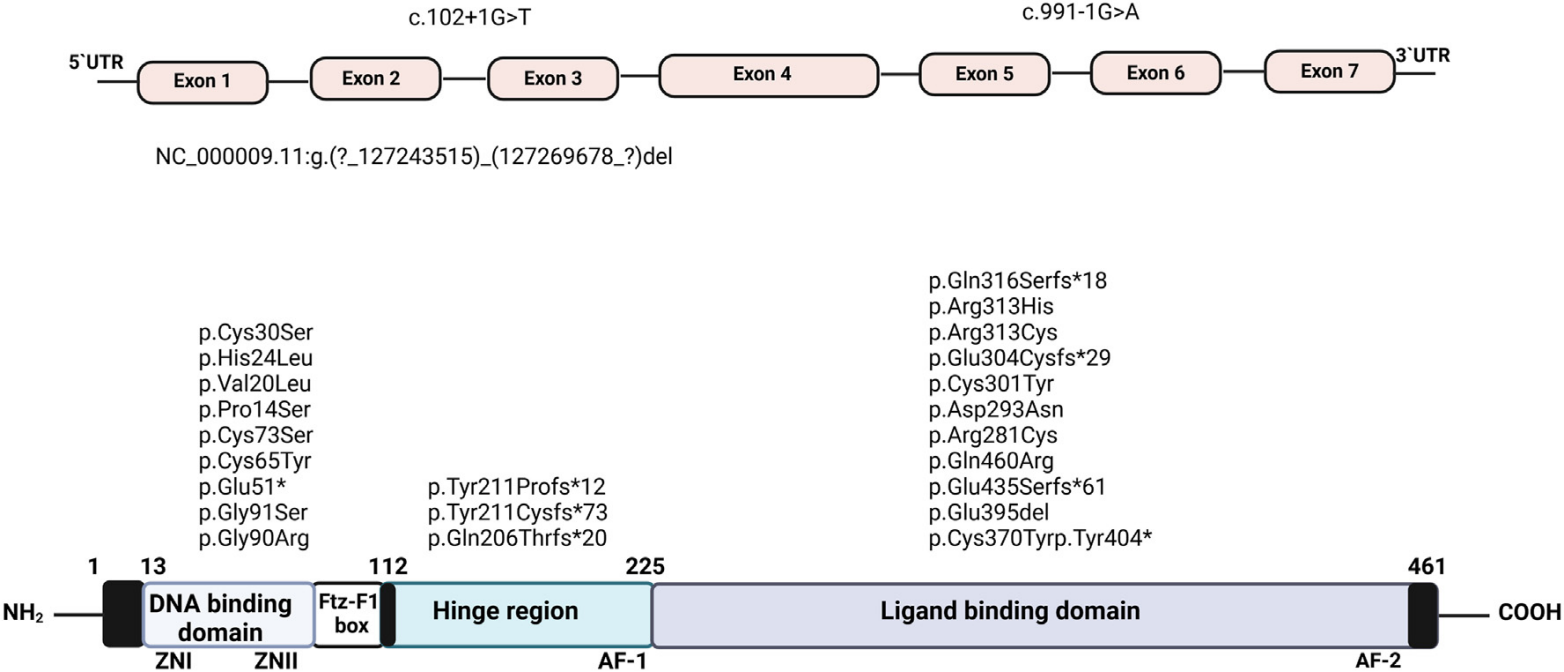
**Summary of the *NR5A1*/SF-1 variants of individuals analysed by newly performed WES analysis for this study, derived from international SF1next study cohort.**^25^ Identified variants in the *NR5A1* gene are shown with respect to the gene and protein sequence. The SF-1 protein comprises the DNA-binding domain, which contains two zinc fingers (Zn1 and Zn2), a Fushi-tarazu factor-1 (FTZ-F1) box, the accessory hinge region, and the ligand-binding domain. It harbours two activation functional (AF) domains, activation function 1 (AF-1) and activation function 2 (AF-2). *NR5A1*, nuclear receptor subfamily 5 group A member 1; UTR, untranslated region.

WES was performed on a total of 30 individuals with *NR5A1*/SF-1 variants and a 46,XY DSD phenotype (index cases) recruited from a larger cohort of 197 individuals participating in the SF1next study^25^; two new individuals were recruited (Supplementary Fig. S1). Twenty of the 30 individuals (67%) had a severe DSD phenotype, and 10 (33%) had an opposite sex phenotype, as previously defined.^25^ Additionally, WES analysis was conducted on 35 family members of the index cases, originating from 18 families; in 12 index cases, family members were not available for WES. Twenty of the 35 family members also carried the respective *NR5A1*/SF-1 variants; of these, six had DSD, one POI, and 13 were asymptomatic. All index cases and family members carried the *NR5A1*/SF-1 variants in a heterozygous state, except for one index case and her cousin who both were homozygous (c.877G > A; p.Asp293Asn). In total 27 different *NR5A1*/SF-1 variants were identified, scattered throughout the whole *NR5A1*/SF-1 gene (Fig. 1). Variants were missense (14/27), small indels (9/27), and splice site mutations (2/27), as well as one large gene deletion, one nonsense mutation and one duplication.

### *NR5A1*/SF-1 related DSD cases without predicted oligogenic variant combinations

Using our specific WES-based variant filtering pipeline and ORVAL testing in search for possible oligogenicity, we did not find any convincing combinations in seven of the 30 (23%) index cases with 46,XY DSD and a *NR5A1*/SF-1 variant (Table 1, Table 2). Index case 1 (Table 1) had a large *NR5A1/*SF-1 gene deletion, preventing inclusion in ORVAL testing, and no additional candidate variants were identified through WES and our specific algorithm filtering. *NR5A1*/SF-1 variants in five index cases (index cases 2,3,4,7,8; Table 1) were located in the ligand binding domain of the SF-1 protein (four deletions, one duplication and one missense mutation), while two deletions of *NR5A1*/SF-1 were located in the hinge region (index cases 5 and 6; Table 1). In five individuals (index case 2–6; Table 1), our analysis revealed no additional variants. Thus, their DSD phenotype may be explained by the heterozygous *NR5A1*/SF-1 variant alone or by other genetic or non-genetic contributing factors, which remained unrecognised by ORVAL. Two individuals (index cases 7 and 8, Table 2) had an additional variant of uncertain significance (VUS) in a DSD-related gene, but in ORVAL testing the combination of the specific variants with *NR5A1*/SF-1 variants did not show an oligogenic prediction. However, index case 7 (Table 2) and her cousin with same opposite sex DSD phenotype, were both identified with an additional heterozygous VUS in the *COL9A3* gene (c.43_48del; p.(Leu15_Leu16del)), which is involved in male gonadal formation.^50^^,^^51^ Another heterozygous variant in this gene has been previously reported as a disease-causing variant/modifier in combination with variants in the *MAMLD1, CDH23, NOTCH1,* and *MAML1* genes in a 46,XY DSD individual with female-typical external genitalia, and has therefore been suggested an oligogenic DSD.^16^

**Table 1:** Clinical and genetic characteristics of index cases without predicted oligogenic variant combinations.

Index case	Karyotype/Sex of rearing	Clinical phenotype	*NR5A1*/SF-1 variant (Zygosity), ACMG	Family tested (Zygosity), DSD-Health
1	46,XY, Female	External genitalia: typical female Internal genitalia: testes Other anomalies: spleen hypoplasia, thrombocytosis & other syndromic features Opposite sex–PGD	NC_000009.11:g.(?_127243515)_(127269678_?)del (Het), Pathogenic	NA
2	46,XY, Female	External genitalia: perineal meatal opening, impalpable gonads, genital tubercle <10 mm, labioscrotal fusion: unfused Internal genitalia: hypoplastic uterus, streak gonads Opposite sex–CGD	c.910_913delGAGC; p.Glu304Cysfs∗29 (Het), Likely pathogenic	Mother (Het), Healthy Sister (WT), Healthy
3	46,XY, Male	External genitalia: meatal opening penoscrotal, gonads labioscrotal or inguinal, genital tubercle 21–30 mm, labioscrotal fusion: unfused Internal genitalia: testes Severe–NSDU	c.1303del; p.Glu435Serfs∗61 (Het), Likely pathogenic	Mother (WT), Healthy Father (Het), Micropenis
4	46,XY, Female	External genitalia: meatal opening perineal, gonads labioscrotal, genital tubercle 10–20 mm, labioscrotal fusion: posterior fusion Internal genitalia: dysgenetic testes Severe–PGD	c.946delC; p.Gln316Serfs∗18 (Het), Likely pathogenic	NA
5	46,XY, Male	External genitalia: hypospadias, cryptorchidism, microphallus Severe–PGD	c.632_668del, p.Tyr211Cysfs∗73 (Het), Likely pathogenic	Mother (Het), Healthy
6	46,XY, Male	External genitalia: meatal opening penoscrotal, gonads labioscrotal, genital tubercle 10–20 mm, labioscrotal fusion: fused Internal genitalia: testes Other anomalies: spleen hypoplasia & thrombocytosis Severe–PGD	c.630_637delGTACGGCT; p.Tyr211Profs∗12 (Het), Likely pathogenic	Mother (Het), Healthy Aunt (Het), POI Grandfather (Het), Hypospadias

ACMG, American College of Medical Genetic; CGD, complete gonadal dysgenesis; DSD, differences of sex development, Het, heterozygous; WT, Wild-type; PGD, partial gonadal dysgenesis; POI, primary ovarian insufficiency; NSDU, Non-specific disorder of under masculinisation; *NR5A1*, NM_004959.5.

**Table 2:** Clinical and genetic characteristics of two index cases with a *NR5A1*/SF-1 variant and an additional gene variant, for which ORVAL showed no prediction for oligogenic variant combinations.

Index case	Karyotype/Sex of rearing	Clinical phenotype	*NR5A1*/SF-1 variant (Zygosity), ACMG	Family tested (Zygosity), DSD-Health	Additional variants (Zygosity)	Found in	gnomAD AF (v3.2.1)	Clinical significance^a^ VarSome/Franklin	CADD
7	46,XY, Female	External genitalia: typical female Internal genitalia: hypoplastic uterus, streak gonads Opposite sex–CGD	c.877G > A; p.Asp293Asn (Hom), Likely pathogenic	Cousin (46,XY) with similar DSD phenotype (Hom)	*COL9A3*: c.43_48del; p.(Leu15_Leu16del)	Index & Cousin	5,63E-05	VUS/VUS	8.77
8	46,XY, Female	External genitalia: typical female Internal genitalia: streak gonads Other anomalies; accessory spleen Opposite sex–CGD	c.1157_1211dup; p.Tyr404^a^ (Het), Likely pathogenic	NA	*DHX37*: c.904G > A; p.(Gly302Ser) (Het)	Index	3,94E-05	VUS/VUS	24.9

a ACMG classification; CGD, complete gonadal dysgenesis; DSD, differences of sex development; Het, heterozygous; Hom, homozygous; PGD, partial gonadal dysgenesis; POI, primary ovarian insufficiency; VUS, Variant of unknown significance; *NR5A1,* NM_004959.5; *COL9A3,* NM_001853.4; *DHX37,* NM_032656.4.

Index case 8 (Table 2) with an opposite sex DSD phenotype carried a heterozygous VUS in the *DHX37* gene (c.904G > A; p.(Gly302Ser)), which is involved in male gonadal formation. Heterozygous variants in the *DHX37* gene have been reported in individuals with 46,XY gonadal dysgenesis and testicular regression.^52^, ^53^, ^54^ In addition, digenic inheritance of heterozygous *DHX37* variants in combination with *NR5A1/SF-1* variants has been previously reported in two individuals with 46,XY DSD.^55^

### *NR5A1*/SF-1 related DSD cases with predicted oligogenic variant combinations

**Fig. 2:**
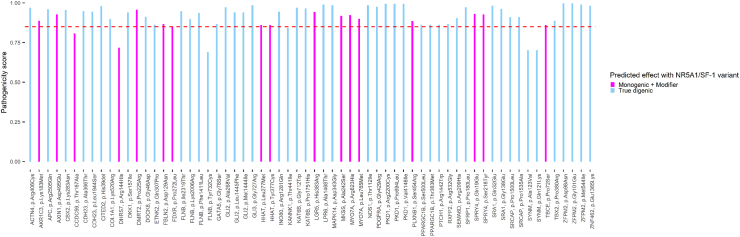
**Bar plot of the VarCoPP score of 64 rare gene variants found in oligogenic combinations with *NR5A1*/SF-1 variants in 22 cases with DSD.** The pathogenicity score (VarCoPP score) generated by ORVAL’s VarCoPP tool represents the probability (value between 0 and 1) that a variant combination belongs to the disease-causing class. If this score is above 0.4575 (hg38), the model predicts that the combination is disease-causing. For stricter analysis a pathogenicity score ≥0.85 (hg38) (dotted red line) was set as the threshold to include only gene pairs with combinations falling into the 99.9%-confidence zone. For one candidate variant (*NR1H2*, p.Arg171_Lys172insAsn) no prediction was found in ORVAL. Predicted digenic effect by ORVAL’s digenic effect predictor tool^47^^,^^49^ for the combination of *NR5A1*/SF-1 variants of each case with the additional variants are indicated by two colours: True digenic combination (blue), where the simultaneous presence of a pathogenic allele in each gene is necessary for the individual to express the disease phenotype. Monogenic and Modifier combination (violet), where a variant on the major gene induces a disease phenotype, while a mutation in the modifier gene modifies it.

**Fig. 3:**
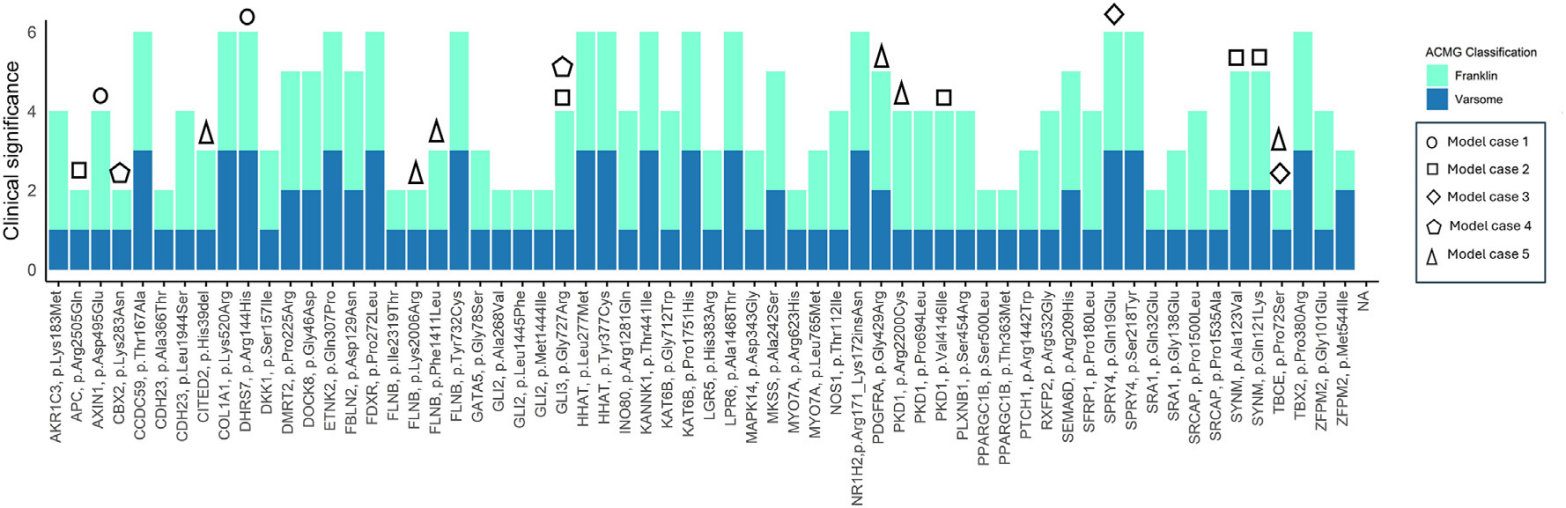
**Stacked bar plot for 22 cases with DSD harbouring *NR5A1*/SF-1 variants showing the predicted pathogenicity of 65 variants based on Franklin (aqua green) and VarSome (blue) classifications.** Clinical significance is given according to ACMG criteria for variants classification: 1 (Benign), 2 (Likely Benign), 3 (Variant of Uncertain Significance, VUS), 4 (Likely Pathogenic), 5 (Pathogenic). Note that none of the variants were predicted pathogenic or likely pathogenic, but need to be included when considering oligogenicity. Symbols indicate model cases that are described in detail in the text.

Twenty-two index cases (22/30, 73%) with *NR5A1*/SF-1 variants were tested in ORVAL, after filtering their WES by our tailored algorithm and finding several additional candidate variants. A summary of the clinical and genetic characteristics of these 22 index cases is shown in Table 3 (short version) and Supplementary Table S2 (comprehensive version). We found 65 variants in 46 distinct genes: 34 in DSD-related genes, seven in DSD and SF-1-related genes and five in SF-1-related genes (Supplementary Table S1 and S3). Fig. 2 shows the pathogenicity scores of these variants in combination with the specific *NR5A1*/SF-1 variant as identified in the index cases. The Digenic Effect predictor in ORVAL classified most of the combinations as “true digenic” (48/65, 74%), while the others were suggested to be “modifiers”. Almost all variants occurred in a heterozygous state and were mostly missense variants, except for one insertion and one deletion. Their predicted clinical significance is depicted in Fig. 3. The average CADD Phred-like score of the tested variants was 23.5, with scores ≥15 indicating a significant likelihood of being deleterious^41^^,^^56^ (Supplementary Fig. S2). Each index case harboured between one to seven candidate variants, additional to the *NR5A1*/SF-1 variant (Table 3). Details on the rejected variants, due to the lack of an association with the observed phenotype of the individuals (according to literature), are given in Supplementary Table S4.

**Table 3:** Clinical and genetic characteristics of DSD index cases with *NR5A1*/SF-1 and additional gene variants suggesting oligogenic inheritance according to ORVAL.^34^

Index case	Karyotype/Sex of rearing	Clinical DSD phenotype	*NR5A1*/SF-1 variant (Zygosity, index case), ACMG	Additional gene variants (Zygosity)	ORVAL score^a^	Clinical significance^b^ VarSome/Franklin	CADD
9	46,XY Female	Opposite sex–CGD	c.902G > A; p.Cys301Tyr (Het), Likely pathogenic	*PKD1*: c.6598C > T; p.(Arg2200Cys) (Het)	0.9950	B/VUS	22.9
				*CITED2*: c.117_119del; p.(His39del) (Het)	0.9800	B/LB	22.0
				*PDGFRA*: c.1285G > A; p.(Gly429Arg) (Het)	0.9775	LB/VUS	22.4
				*FLNB*: c.4233C > G; p.(Phe1411Leu) (Het)	0.9375	B/LB	21
				*FLNB*: c.6017A > G; p.(Lys2006Arg) (Het)	0.9375	B/LB	21
				*TBCE*: c.214C > T; p.(Pro72Ser) (Het)	0.8550	B/B	23.2
10	46,XY Male	Severe–NSDU	c.841C > T; p.Arg281Cys (Het), Likely pathogenic	*ZFPM2*: c.292G > A; p.(Asp98Asn) (Het)	0.9975	B/B	23.9
				*CCDC59*: c.499A > G; p.(Thr167Ala) (Het)	0.8075	VUS/VUS	25.3
11	46,XY Male	Severe–PGD	c.1109G > A; p.Cys370Tyr (Het), Likely pathogenic	*GLI3*: c.2179G > A; p.(Gly727Arg) (Het)	0.9850	B/VUS	25.5
				*KANK1*: c.1322C > T; p.(Thr441Ile) (Het)	0.8425	VUS/VUS	24.3
12	46,XY Female	Opposite sex–CGD	c.217T > A; p.Cys73Ser (Het), Likely pathogenic	*GLI3*: c.2179G > A; p.(Gly727Arg) (Het)	0.9850	B/VUS	25.5
				*APC*: c.7514G > A; p.(Arg2505Gln) (Het)	0.9600	B/B	23.8
				*PKD1*: c.12436G > A; p.(Val4146Ile) (Het)	0.9950	B/VUS	23.3
				*SYNM*: c.361C > A; p.(Gln121Lys) (Het)	0.7025	LB/VUS	19.38
				*SYNM*: c.368C > T; p.(Ala123Val) (Het)	0.7025	LB/VUS	20.5
13	46,XY Male	Severe—TDSD	c.40C > T; p.Pro14Ser (Het), VUS	*GLI3*: c.2179G > A; p.(Gly727Arg) (Het)	0.9850	B/VUS	25.5
				*CBX2*: c.849G > T; p.(Lys283Asn) (Het)	0.9550	B/B	20.7
14	46,XY Male	Severe—NSDU	c.937C > T; p.Arg313Cys (*de novo*), Pathogenic	*SPRY4*: c.55C > G; p.(Gln19Glu) (Het)	0.9300	VUS/VUS	24.6
				*TBCE*: c.214C > T; p.(Pro72Ser) (Het)	0.8600	B/B	23.2
15	46,XY Female	Opposite sex–CGD	c.614_615insC; p.Gln206Thrfs∗20 (Het), Pathogenic	*INO80*: c.3842G > A; p.(Arg1281Gln) (Het)	0.9450	B/VUS	27.6
				FLNB: c.6956T > C; p.(Ile2319Thr) (Het)	0.9400	B/B	28.0
				*SPRY4*: c.653C > A; p.(Ser218Tyr) (Het)	0.9275	VUS/VUS	27.8
				*MKKS*: c.724G > T; p.(Ala242Ser) (Het)	0.9175	LB/VUS	24.9
				*FDXR*: c.815C > T; p.(Pro272Leu) (Het)	0.8525	VUS/VUS	27.6
16	46,XY Female	Severe–TDSD	c.102+1G > T (Het) (*de novo*), Likely pathogenic	FLNB: c.6956T > C; p.(Ile2319Thr) (Het)	0.9475	B/B	28.0
				*KAT6B*: c.5252C > A; p.(Pro1751His) (Het)	0.9650	VUS/VUS	24.4
				*MYO7A*: c.2293C > A; p.(Leu765Met) (Het)	0.9000	B/LB	23.2
				*PKD1*: c.2081C > T; p.(Pro694Leu) (Het)	0.9950	LB/VUS	25.4
17	46,XY Male	Severe–TDSD	c.938G > A; p.Arg313His (Het), Pathogenic	*SEMA6D*: c.626G > A; p.(Arg209His) (Het)	0.9050	LB/VUS	32
				*PDGFRA*: c.1285G > A; p.(Gly429Arg) (Het)	0.9750	LB/VUS	16.3
				*ZNF462*: c.4093G > A; p.(Glu1365Lys) (Het)	0.9825	B/VUS	25.1
18	46,XY Male	Severe–PGD	c.937C > T; p.Arg313Cys (Het), Pathogenic	*DKK1*: c.470G > T; p.Ser157Ile (Het)	0.9400	B/LB	21.7
				*AXIN1:* c.1485C > G; p.(Asp495Glu) (Het)	0.9275	B/VUS	17.2
19	46,XY Female	Severe–PGD	c.194G > A; p.Cys65Tyr (Het), Likely pathogenic	*SFRP1*: c.539C > T; p.(Pro180Leu) (Het)	0.9725	B/VUS	26.8
				*COL1A1:* c.1559A > G*;* p.(Lys520Arg) (Het)	0.8975	VUS/VUS	23.1
20	46,XY Male	Severe–PGD	c.938G > A; p.Arg313His (Het), Pathogenic	*LRP6*: c.4402G > A; p.(Ala1468Thr) (Het)	0.9900	B/VUS	27.4
				*ETNK2*: c.920A > C; p.(Gln307Pro) (Het)	0.8625	VUS/VUS	22.9
21	46,XY Male	Severe–PGD	c.991-1G > A (Het) (*de novo*), Likely pathogenic	*GLI2*: c.803C > T; p.(Ala268Val) (Het)	0.9725	B/B	25.0
				*CDH23*: c.5831T > C; p.(Leu1944Ser) (Het)	0.9450	B/VUS	22.6
				*LGR5*: c.1148A > G; p.(His383Arg) (Het)	0.9425	B/LB	22.3
				*GATA5*: c.232G > A; p.Gly78Ser (Het)	0.8675	B/LB	15.44
				*PPARGC1B*: c.1088C > T; p.(Thr363Met) (Het)	0.8625	B/B	17.2
				*PPARGC1B*: c.1499C > T; p.(Ser500Leu) (Het)	0.8625	B/B	10.03
22	46,XY Male	Severe–PGD	c.71A > T; p.His24Leu (Het), Likely pathogenic	*MAPK14*: c.1028A > G; p.(Asp343Gly) (Het)	0.9850	B/LB	23.4
				*PLXNB1*: c.1360A > C; p.(Ser454Arg) (Het)	0.8850	B/VUS	25.7
				*PTCH1*: c.4324C > T; p.(Leu277Met) (Het)	0.8625	B/LB	20.2
				*HHAT*: c.829C > A; p.(Leu277Met) (Het)	0.8600	VUS/VUS	23.8
				*HHAT*: c.1130A > G; p.(Tyr377Cys) (Het)	0.8600	VUS/VUS	23.0
23	46,XY Female	Opposite sex–PGD	c.151G > T; p.Glu51^b^ (Het), Pathogenic	*SRA1*: c.413G > A; p.(Gly138Glu) (Het)	0.9625	B/LB	19.2
				MYO7A: c.1868G > A; p.(Arg623His) (Het)	0.9225	B/B	26.8
				*SRCAP*: c.4499C > T; p.(Pro1500Leu) (Het)	0.9100	B/VUS	22.1
				*SRCAP*:c.4603C > G; p.(Pro1535Ala) (Het)	0.9100	B/B	17.8
24	46,XY Female	Severe–PGD	c.1379A > G; p.Gln460Arg (Het), VUS	*TBX2*: c.1139C > G; p.(Pro380Arg) (Het)	0.8875	VUS/VUS	24.7
				*FLNB*: c.2195A > G; p.(Tyr732Cys) (Het)	0.6900	VUS/VUS	23.8
25	46,XY Male	Severe–Gonadal regression	c.271G > A; p.Gly91Ser (Het), Likely pathogenic	*NOS1*: c.335C > T; p.(Thr112Ile) (Het)	0.9850	B/VUS	23.2
				*FLNB*: c.6017A > G; p.(Lys2006Arg) (Het)	0.8975	B/B	21.0
				*AKR1C3*: c.548A > T; p.Lys183Met (Het)	0.8875	B/VUS	25.6
				*DHRS7*: c.431G > A; p.(Arg144His) (Het)	0.7175	VUS/VUS	26.7
				*KAT6B:* c.2134G > T*; p.(Gly712Trp) (Het)*	0.9700	B/VUS	28.0
				RXFP2: c.1594C > G; p.(Arg532Gly) (Het)	0.8675	B/VUS	20.4
27	46,XY Male	Opposite sex–CGD	c.1183_1185delGAG; p.Glu395del (Het), Likely pathogenic	*ZFPM2*: c.1632G > A; p.(Met544Ile) (Het)	0.9900	LB/B	20.5
28	46,XY Male	Severe–PGD	c.58G > C; p.Val20Leu (Het), Likely pathogenic	*CDH23*: c.1096G > A; p.(Ala366Thr) (Het)	0.9475	B/B	25.6
				*NR1H2*: c.515_516insCAA; p.(Arg171_Lys172insAsn) (Het)	ND	VUS/VUS	ND
29	46,XY Female	Opposite sex–PGD	c.268G > C; p.Gly90Arg (Het), VUS	*ZFPM2*: c.302G > A; p.(Gly101Glu) (Het)	0.9975	B/VUS	25.2
				*SRA1*: c.94C > G; p.(Gln32Glu) (Het)	0.9825	B/B	26.7
				*FBLN2*: c.385G > A; p.(Asp129Asn) (Het)	0.8675	LB/VUS	29.1
30	46,XY Female	Opposite sex–PGD	c.614_615insC; p.Gln206Thrfs∗20 (Het)	*GLI2*: c.4332G > A; p.(Met1444Ile) (Het)	0.9400	B/B	15.95
				*GLI2*: c.4333C > T; p.(Leu1445Phe) (Het)	0.9400	B/B	22.4

Further details are provided in Supplementary Table S2.

a Pathogenicity score with *NR5A1*/SF-1 variant (ORVAL).

b ACMG American College of Medical Genetics classification, CGD, complete gonadal dysgenesis; DSD, differences of sex development, Het, heterozygous; Hom, homozygous; PGD, partial gonadal dysgenesis; CGD, complete gonadal dysgenesis; DSD, differences of sex development, Het, heterozygous; Hom, homozygous; PGD, partial gonadal dysgenesis; NSDU, Non-specific disorder of under masculinisation, TDSD; testicular DSD; ND, not defined, B, Benign; LB, Likely Benign; VUS, Variant of unknown significance; *NR1H2*: c.515_516insCAA; p.(Arg171_Lys172insAsn), not defined in ORVAL but included in the analysis due to pathogenicity (VUS). *FLNB*: c.2195A > G; p.(Tyr732Cys). *DHRS7*: c.431G > A; p.(Arg144His), *KANK1*: c.1322C > T; p.(Thr441Ile), *CCDC59*: c.499A > G; p.(Thr167Ala), *SYNM*: c.361C > A; p.(Gln121Lys), *SYNM*: c.368C > T; p.(Ala123Val) below ORVAL threshold (≥0.85 (hg38)) but included in the analysis due to their pathogenicity (VUS). *AKR1C3*, NM_003739.6; *APC*, NM_000038.6; *AXIN1*, NM_003502.4; *CBX2*, NM_005189.3; *CCDC59*, NM_014167.5; *CDH23*, NM_022124.6; *CITED2*, NM_006079.5; *COL1A1*, NM_000088.4; *DHRS7*, NM_016029.4; *DKK1*, NM_012242.4; *ETNK2*, NM_018208.4; *FBLN2*, NM_001004019.2; *FDXR*, NM_024417.5; *FLNB*, NM_001457.4; *GATA5*, NM_080473.5; *GLI2*, NM_005270.5; *GLI3*, NM_000168.6; *HHAT*, NM_018194.6; *INO80*, NM_017553.3; *KANK1*, NM_015158.5; *KAT6B*, NM_012330.4; *LGR5*, NM_003667.4; *LRP6*, NM_002336.3; *MAPK14*, NM_139012.3; *MKKS*, NM_170784.3; *MYO7A*, NM_000260.4; *NOS1*, NM_000620.5; *NR1H2*, NM_007121.7; *NR5A1,* NM_004959.5; *PDGFRA*, NM_000358.3; *PKD1*, NM_001009944.3; *PLXNB1*, NM_001130082.3; *PPARGC1B*, NM_133263.4; PTCH1, NM_000264.5; RXFP2, NM_130806.5; *SEMA6D*, NM_001358351.3/ENST00000536845.7; *SFRP1*, NM_003012.5; SPRY4, NM_001127496.3; *SRA1*, ENST00000336283.6; *SRCAP*, NM_006662.3; *SYNM*, NM_145728.3; *TBCE*, NM_003193.5; TBX2, NM_005994.4; *ZFPM2*, NM_012082.4; *ZNF462*, NM_021224.6.

Interestingly, we identified the same additional gene variants in eight unrelated cases with DSD in combination with different *NR5A1*/SF-1 variants.

A heterozygous *GLI3* variant (c.2179G > A; p.(Gly727Arg)) was found in three unrelated DSD cases (index cases 11,12 and 13, Table 3), inherited from their healthy fathers, who did not carry the *NR5A1*/SF-1 variant. All cases had penoscrotal or scrotal hypospadias. GLI3 is a transcription factor involved in male sex differentiation and external genitalia formation,^57^ and *GLI3* variants are described to be associated with hypospadias,^58^ cryptorchidism,^59^ micropenis,^60^ hypogonadotropic hypogonadism^61^ and oligogenic 46,XY DSD.^15^

A variant in the *TBCE* gene (c.214C > T; p.(Pro72Ser)) was found in two 46,XY DSD cases (index cases 9 and 14, Table 3) with inguinal gonads, opposite sex and severe DSD phenotype. The *TBCE* gene is involved in neurodevelopment disorders such as Hypoparathyroidism-Retardation-Dysmorphism Syndrome (OMIM: 241,410), which is associated with micropenis and cryptorchidism.^62^^,^^63^ In a previously reported case of 46,XY DSD with bilateral cryptorchidism, a *TBCE* variant was suggested as the disease-causing variant.^64^

In two other DSD cases (index cases 15 and 16) with severely undervirilised external genitalia, a heterozygous *FLNB* variant (c.6956T > C; p.Ile2319Thr) was detected. Biallelic *FLNB* variants have been reported in a 46,XY DSD individual with female external genitalia and skeletal dysplasia,^65^ and are implicated in Larsen syndrome (OMIM:150,250), which is associated with cryptorchidism.^66^

In addition, a heterozygous variant in the *PDGFRA* (c.1285G > A; p.(Gly429Arg)) gene was found in two 46,XY DSD cases (index cases 9 and 17, Table 3) with opposite sex and severe DSD phenotype. The *PDGFRA* gene seems a crucial mediator for male gonadal formation^67^ and has been associated with anorectal malformations and hypospadias in humans.^68^

Apart from those repeatedly observed variants, different variants in eight specific genes were observed two to five times across 15 studied DSD cases in combination with various *NR5A1*/SF-1 variants. These were variants in genes previously associated with DSD (Table 3), including *CDH23* (n = 2), *FLNB* (n = 5), *GLI2* (n = 2), *KAT6B* (n = 2), *MYO7A* (n = 2), *PKD1* (n = 3), *SPRY4* (n = 2), and *ZFPM2* (n = 3).

**Fig. 4:**
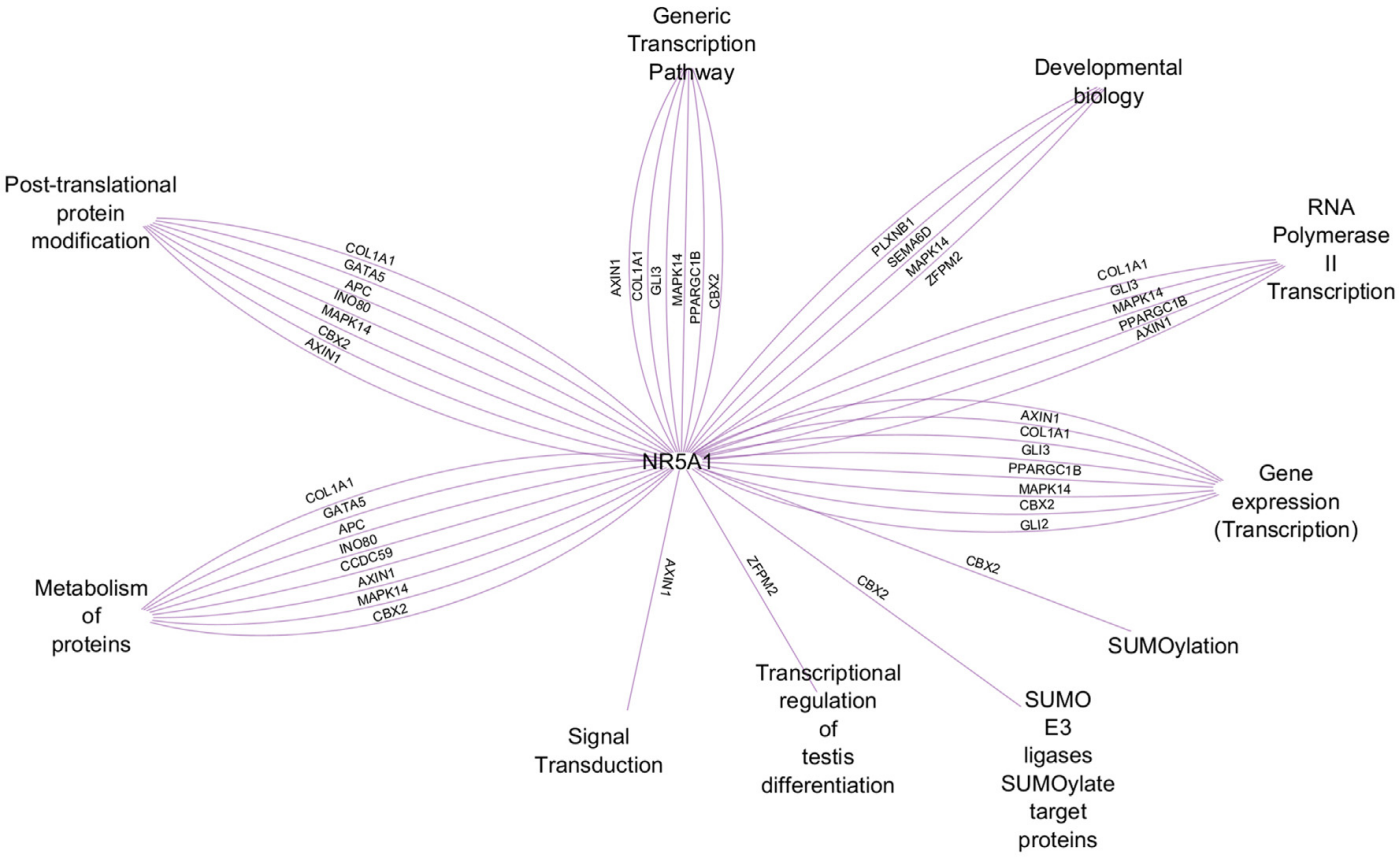
**Summary of common pathways identified between the *NR5A1* gene and 14 other genes, in which additional variants were found in our study participants.** The analysis was performed with Reactome and the visualisation with Cytoscape.^69^

To better understand the collaborative network of SF-1, we searched for common pathways between the *NR5A1* gene and the 46 genes with identified additional variants of our study participants using Reactome. This analysis revealed common pathways for 14 genes (30%). These shared pathways included transcription and gene expression, developmental biology, metabolism of proteins, post-translational modifications, and signal transduction (Fig. 4).

### Consideration of oligogenic DSD in combination with *NR5A1*/SF-1 variants through trio analysis in five model cases

#### Model case 1

**Fig. 5:**
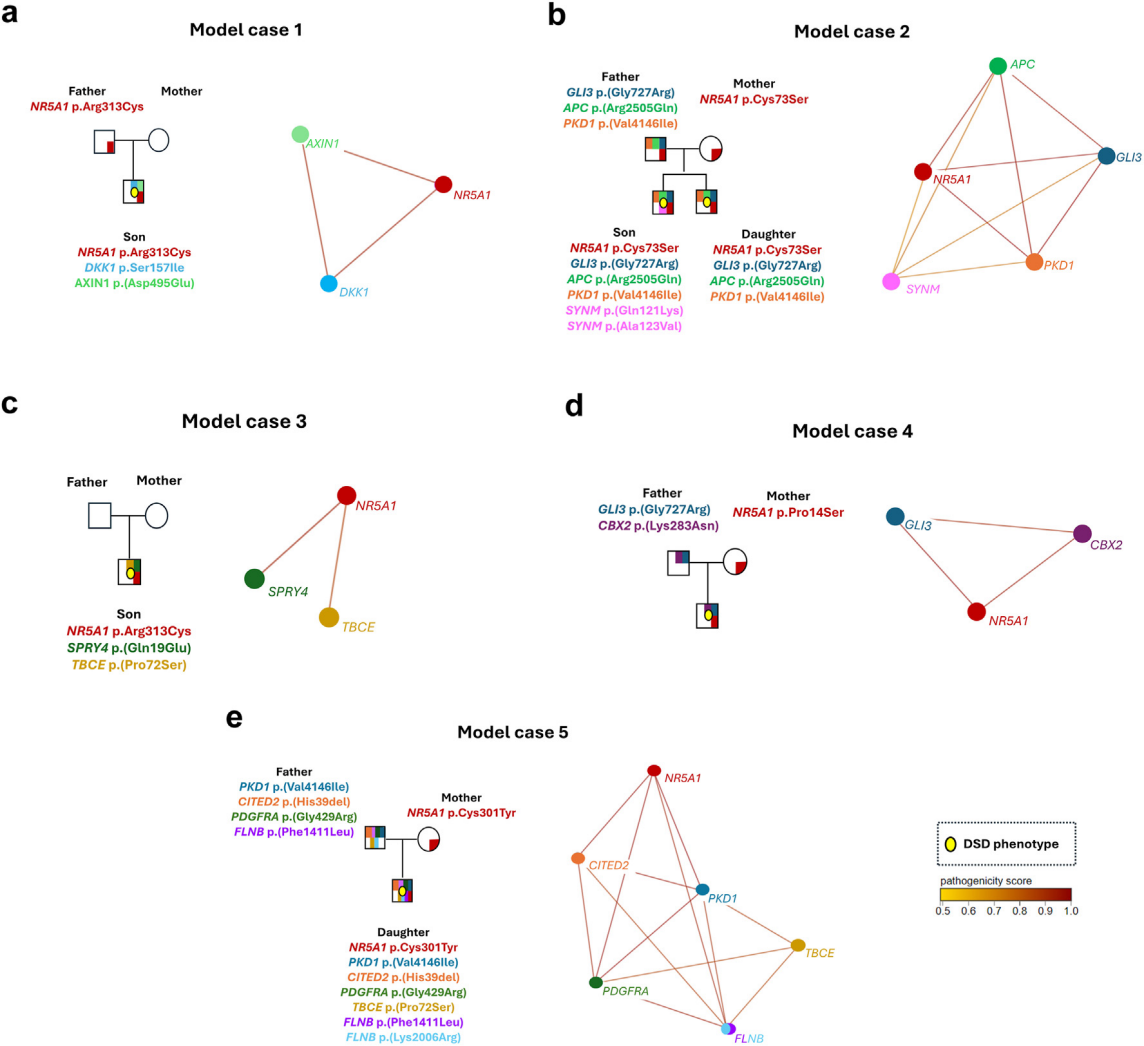
**Family trees showing the individual gene variants (left panel) and the predicted oligogenic network (right panel) in five model cases (a–e).** The pedigrees depict the inheritance patterns of the identified variants. Note that all variants were observed in a heterozygous state. The networks created for each case by ORVAL inform on predicted gene interactions necessary to reveal a disease phenotype.

This male with a severe 46,XY DSD phenotype (index case 18, Table 3, Fig. 3, Fig. 5a) inherited a heterozygous *NR5A1*/SF-1 variant (c.937C > T, p.Arg313Cys) from his healthy father; the mother was wild-type (WT) for *NR5A1*/SF-1. The p.Arg313Cys SF-1 variant has been reported in heterozygous state in five other patients: three males with a less severe DSD phenotype (glandular/scrotal hypospadias with or without microphallus)^70^, ^71^, ^72^ than observed in our case; one female with 46,XY gonadal dysgenesis,^73^ and in model case 3 (Fig. 5C). Functional testing of this *NR5A1*/SF-1 variant showed impaired transactivation activity.^70^, ^71^, ^72^, ^73^ In addition, *in vitro* cellular reprogramming using induced pluripotent stem cells from the female patient with 46,XY gonadal dysgenesis, showed abnormal expression of gonadal transcripts and absence of tubules formation.^74^ WES analysis of model case 1 revealed two additional heterozygous variants in the index DSD case only: *AXIN1* c.1485C > G; p.(Asp495Glu) and *DKK1* c.470G > T; (p.Ser157Ile) (Fig. 5a). *AXIN1* promotes male gonadal formation and inhibits ovarian development.^2^^,^^75^ *AXIN1* variants have been associated with cryptorchidism.^76^ The DKK1 *gene* is crucial for the development of the anorectal and genitourinary tract.^77^ A heterozygous *DKK1* variant has been reported in an individual with anorectal malformation and hypospadias.^78^ The phenotype of model case 1 may be explained by the contribution of the additional variants, as the father is a carrier of the *NR5A1*/SF-1 variant and is completely asymptomatic. However, whether both or predominantly one of the additional variants contribute to the observed phenotype remains open.

#### Model case 2

This is a family with two siblings with a severe/opposite sex 46,XY DSD, one assigned female and one male, respectively. Both inherited a heterozygous *NR5A1*/SF-1 variant (c.217T > A; p.Cys73Ser) from their asymptomatic mother (index case 12, Table 3, Fig. 3, Fig. 5b). The p.Cys73Ser SF-1 variant was newly identified in our SF1next cohort^25^ and classified as pathogenic according to ACMG criteria. This SF-1 variant showed WT activity in previously performed *in vitro* functional tests.^79^ Trio WES identified three heterozygous candidate variants: *GLI3* (c.2179G > A; p.(Gly727Arg)), *APC* (c.7514G > A; p.(Arg2505Gln)) and *PKD1* (c.12436G > A; p.(Val4146Ile)) in both siblings and the healthy father, who is WT for *NR5A1*/SF-1. The transcription factor GLI3 is involved in male sex differentiation and external genitalia formation^57^ and *GLI3* variants have been associated with hypospadias,^58^ cryptorchidism,^59^ hypogonadotropic hypogonadism^61^ and oligogenic 46,XY DSD.^15^ The APC gene acts as a negative regulator of Wnt signalling pathway and decreases SF-1 mediated activation of the Mullerian inhibiting substance type II receptor (MISRII or AMHR2) promoter, which is crucial for Müllerian duct regression in males. *PKD1* variants cause autosomal dominant polycystic kidney disease, which involves reproductive tract abnormalities and infertility in males^80^; while an oligogenic role in 46,XY DSD has been previously suggested.^38^^,^^81^ Comparison of the genotype between the siblings showed two additional *de novo* variants in the *SYNM* gene: c.361C > A; p.(Gln121Lys) and c.368C > T; p.(Ala123Val)), present only in the sibling with the opposite sex DSD. *SYNM* variants have been reported in cases of Ulnar-Mammary-Like Syndrome,^82^ in which genital defects such as micropenis and cryptorchidism have been observed in rare cases.^83^ The *SYNM* variants had a lower CADD score and by the digenic effect predictor were predicted having a “modifier effect” with the *NR5A1*/SF-1 variant, while all other identified variants of both siblings were predicted having “true digenic” effects. Taken together, these findings suggest that the *GLI3, APC, PKD1*, and *NR5A1* variants may work together in an oligogenic network influencing the specific DSD phenotype (Fig. 5b).

#### Model case 3

A male with a *de novo* heterozygous *NR5A1*/SF-1 variant (c.937C > T, p.Arg313Cys) (index case 14, Table 3, Fig. 3, Fig. 5c) presented with severely undervirilised external genitalia and hypogonadism at birth. This SF-1 variant was also identified in the model case 1, as well as in other three patients with mild or severe DSD.^70^, ^71^, ^72^, ^73^ Trio WES revealed two additional heterozygous variants only in the index case. One variant was found in the *SPRY4* gene (c.55C > G; p.(Gln19Glu)), which is associated with hypogonadotropic hypogonadism, where the congenital form was also linked to oligogenic inheritance.^19^^,^^84^, ^85^, ^86^ The other variant was identified in the TBCE gene (c.214C > T; p.(Pro72Ser)), previously mentioned to be related to micropenis and cryptorchidism.^62^, ^63^, ^64^

#### Model case 4

A male with a severe 46,XY DSD phenotype (index case 13, Table 3, Fig. 3, Fig. 5d) inherited a heterozygous *NR5A1*/SF-1 variant (c.40C > T, p.Pro14Ser) from his mother, who underwent ovarian stimulation for conception, and previously had three miscarriages. The p.Pro14Ser SF-1 variant was newly identified in our SF1next cohort,^25^ classified as VUS according to ACMG criteria and showed activity similar to WT *in vitro*.^79^ In trio WES two additional variants were found in the index case and the healthy father, who was WT for *NR5A1*/SF-1. One in the *CBX2* (c.849G > T; p.(Lys283Asn)) gene and one in the *GLI3* (c.2179G > A; p.(Gly727Arg)) gene. The *CBX2* gene plays an important role in gonadal formation,^87^ and activates *NR5A1* expression during testis development.^88^^,^^89^ In addition, compound heterozygous *CBX2* variants were reported in a female with 46,XY DSD.^89^ *GLI3* variants have been identified in other cases of our cohort (see above).

#### Model case 5

A 46,XY DSD individual female (index case 9, Table 3, Fig. 3, Fig. 5e) had inherited a heterozygous *NR5A1*/SF-1 variant (c.902G > A; p.Cys301Tyr) from her asymptomatic mother. This SF-1 variant has previously revealed normal activity in functional testing.^90^ WES of the whole family identified six heterozygous variants: *PDGFRA* (c.1285G > A; p.(Gly429Arg)), *PKD1* (c.6598C > T; p.(Arg2200Cys)), *CITED2* (c.117_119del, p.(His39del)), *FLNB* (c.4233C > G; p.(Phe1411Leu)) were found in the index case and her father (WT for *NR5A1*/SF-1 variant), while *TBCE* (c.214C > T, p.(Pro72Ser)) and *FLNB* (c.6017A > G; p.(Lys2006Arg)) variants were only found in the index case. Variants in *PDGFRA, PKD1, TBCE,* and *FLNB* genes are all associated with a DSD phenotype, consistent with observations in other cases in our cohort (index cases 17,12,16,14,25). It is important to note that this index case has compound heterozygous variants in the *FLNB* gene, which aligns with another reported case of 46,XY sex reversal and skeletal dysplasia caused by biallelic mutations in *FLNB*.^65^ The *CITED2* variant was uniquely identified in this specific index case from our cohort. CITED2 is an important transcription co-regulator in early male gonadal formation. It interacts with SF-1 to enhance its transcriptional activity, which is crucial for proper gonadal development.^91^, ^92^, ^93^, ^94^ *CITED2* deficiency is associated with gonadal defects in mice, including sex reversal^52^ and premature ovarian failure in humans.^95^

## Discussion

In this study, we explored possible oligogenic patterns in 30 individuals and family members with a broad range of DSD and *NR5A1*/SF-1 variants, recruited from the SF1next study cohort,^25^ by conducting WES, oligogenicity testing in ORVAL bioinformatics platform, and phenotype-guided data analysis on identified variants. Oligogenicity was identified in 22 individuals with 46,XY DSD (73%); each carrying one to seven additional variants, predominantly in DSD-related genes, that likely contribute to the DSD phenotype. The combinations varied between individuals, though common variants were identified in genes such as *TBCE, FLNB*, *GLI3* and *PDGFRA*. In addition, variants in eight different genes were more frequently identified together with *NR5A1*/SF-1 variants in 15 index cases; these genes were all previously associated with DSD, including *CDH23, FLNB, GLI2, KAT6B, MYO7A, PKD1, SPRY4*, and *ZFPM2*. Furthermore, two individuals had additional candidate variants in DSD-related genes, but they were not predicted to form oligogenic combinations with the *NR5A1*/SF-1 variants in ORVAL. In seven individuals, our WES analysis did not reveal additional candidate variants, indicating that their DSD phenotype might be explained by high penetrant variants in *NR5A1*/SF-1 or by other, yet undiscovered variants or mechanisms. Interestingly, most of these variants were truncating, resulting in severe loss-of-function effects, except for one missense in the homozygous state and one nonsense variant, while the majority of the variants in *NR5A1* in the rest cohort were missense. Taken together, our data suggest that in about three out of four DSD individuals with *NR5A1*/SF-1 variants, additional variants in DSD associated genes can be found that may act as disease modifiers of the phenotype. Thus, the vast spectrum of additional genetic hits discovered can possibly explain the broad phenotype observed in many individuals with *NR5A1*/SF-1-related DSD and their family members.

The findings in our large SF1next study cohort confirm genetic data from previous case reports and smaller case series of individuals with DSD harbouring *NR5A1*/SF-1 variants, in which a total of more than 50 additional variants in 42 genes have been identified.^15^^,^^30^^,^^38^^,^^55^^,^^72^^,^^73^^,^^96^, ^97^, ^98^, ^99^, ^100^, ^101^, ^102^, ^103^, ^104^, ^105^, ^106^, ^107^, ^108^, ^109^, ^110^, ^111^ For example, variants in the *GLI2* gene were identified in our cohort and have been reported in other cases of 46,XY DSD associated with *NR5A1/*SF-1^38^; but *GLI2* variants were also seen in combination with variants in other genes than *NR5A1*/SF-1 in DSD.^15^^,^^112^ Likewise, variants in the *SRA1* gene were identified in our cohort in combination with *NR5A1*/SF-1 and in a previous study.^104^ Additionally, variants in the *ZFPM2* gene seem to play a role in oligogenic aetiology of DSD, particularly in individuals with *NR5A1*/SF-1 variants.^30^^,^^96^ As some specific genes were found prevalently in our SF1next cohort and in previous reports, these genes may form a core network preferentially working together with SF-1 in sex development (Fig. 4). Yet, our study, along with other reports, identified a wide range of potential oligogenic combinations with *NR5A1*/SF-1, including many unique combinations only found in single patients or families. Additionally, variants in genes that so far have not been associated with DSD and SF-1 were found. Given the rarity of DSD due to *NR5A1*/SF-1 variants and the limited number of individuals analysed so far, it remains difficult to fully map the exact gene networks and predict their disease-causing effects when altered in different combinations. Therefore, it is important to recognise that each individual’s genetic makeup contributing to the DSD phenotype is mostly unique and often more complex than initially thought.

Our analysis did not reveal additional variants in seven individuals with *NR5A1*/SF-1 variants and DSD. Of those, five individuals harboured heterozygous deletions in the *NR5A1* gene resulting mostly in frameshift variants and severe alterations of the SF-1 protein. This finding suggests that frameshift variants in *NR5A1* may be sufficient to cause the observed DSD phenotype. However, in two of these cases, family members harbouring the same *NR5A1*/SF-1 variant as the index cases exhibited a less severe DSD phenotype, indicating that still hidden disease modifiers are at stake. Moreover, three other index cases of our cohort had frameshift *NR5A1*/SF-1 variants and were found to have additional genetic variants predicted to contribute to an oligogenic disease mechanism. Other studies have reported four cases with deletions in the *NR5A1* gene and additional variants in genes such as *TBX2, EGF, SRD5A2, DHX37*.^55^^,^^97^^,^^100^^,^^101^^,^^113^ In the remaining two cases, we found additional candidate variants in *COL9A3* and *DHX37* genes, but without prediction in ORVAL; one was homozygous for a missense *NR5A1*/SF-1 variant and the other had a *NR5A1/*SF-1 duplication. The homozygosity may have led ORVAL/VarCoPP to focus on a monogenic effect, while the duplication represents a known limitation of the ORVAL predictions. The specific *NR5A1/*SF-1 variants in these last two cases could therefore be true monogenic. Overall, these negative findings may reflect the known limitations of ORVAL as a tool for accurately predicting combinations of rare or less characterised variants, such as deletions, duplications and frameshifts.^34^ Additionally, ORVAL’s predictions are limited by: a) its design, which includes program-specific data sets and predictive tools, and b) the lack of inclusion of clinical data. As a result, the tool faces constraints in terms of sensitivity and specificity, but so far it remains the only tool widely used. The evidence supporting a reported gene or variant as causative for DSD varies significantly. To prove the disease-causing mechanism of a genetic disorder can be challenging, particularly if an oligogenic causation is suspected. Multiple variants may exert minor modifying effects, which are not individually, but all together pathogenic; and these variants can be located in genes that are either unknown or not typically associated with the disease phenotype.^114^^,^^115^ Indeed, it has been shown that variants participating in oligogenic combinations exhibit distinct characteristics compared to those in monogenic diseases, such as being more prevalent in the general population, and having lower monogenic pathogenicity scores. They also have smaller effects on gene function, or are located in genes that are not typically associated with the same disease.^36^^,^^48^^,^^49^^,^^114^^,^^115^ Functional testing for studying oligogenicity requires integrating multiple variants into *in vitro* (mostly cell) or *in vivo* models to assess their combined effect on biological functions related to the DSD phenotype. Whether patient-derived reprogrammed cell models will help, remains to be tested. These cell models benefit from carrying the specific genetic background of the donor, while other challenges and limitations encountered with cell models continue.^74^^,^^116^, ^117^, ^118^ By contrast, several computational approaches have recently emerged to assess oligogenicity and prioritise causative variants. In our study, we used VarCoPP (integrated in ORVAL), a machine learning tool designed to assess the potential pathogenicity of variant combinations.^46^^,^^47^ However, while VarCoPP can predict oligogenic combinations, additional investigation of each of the identified combinations is necessary for assessing disease-relevance. Another tool currently under development is Hop (High throughput oligogenic prioritiser); Hop aims to streamline the process by evaluating both phenotyping and genotyping information, to score the disease relevance of the predicted combinations.^35^

We are aware that apart from oligogenic inheritance, other mechanisms should also be considered to explain variable expressivity and phenotypic variability associated with *NR5A1*/SF-1 variants; but these mechanisms have only been formulated as hypotheses without confirmation, including: skewed allelic expression, function within a dosage sensitive developmental time window,^119^ epigenetic regulations,^7^^,^^120^ mosaicism^29^ and tissue-specific somatic reversion mechanisms. All these hypothetical mechanisms are challenging to address, especially in humans.

Regarding the strengths of our study, a key advantage lies in the systematic approach we used to analyse individuals with *NR5A1*/SF-1 variants through WES, employing a tailored algorithm for DSD- and SF-1-related genes, with the aim to search and identify oligogenic patterns. This approach included a substantial number of index cases (n = 30) and their family members (n = 35), providing a more representative (so far largest) cohort compared to previous studies reporting single cases or case series. Unlike previous studies, we believe that our study provides a large enough number to address the specific question of oligogenicity in DSD related to *NR5A1*/SF-1, using current state-of-the art tools and algorithms, which, although advanced, are still not without restrictions.

On the other hand, our study has also several limitations: The tailored algorithm was designed to focus on genes related to DSD and SF-1 and thereby may miss out on other genes not yet associated with these phenotypes or the complex process of sex development. Moreover, our WES-based approach did not account for non-coding variants far away from splice sites, as well as variants in regulatory elements, which could also contribute to the DSD phenotype as just recently shown.^8^^,^^118^ As mentioned before, the genetic, bioinformatic/machine learning tools and experimental models currently available for oligogenicity testing and predictions, are still scarce; so far only ORVAL has been used in several studies.^34^^,^^43^^,^^114^^,^^121^^,^^122^ In addition to the limitations we have addressed previously, it is also important to mention that ORVAL always tests the relationship between only two candidates (called “bi-locus combination” by the program); when finding several candidates, the program cannot discriminate, which variants’ combinations are more likely disease causing. In addition, more extended family studies would maybe help in segregating variants. Further investigation of our cohort could benefit from integrating WES data with gene expression (RNA sequencing) data. This combined analysis could help to understand the effect of the genetic background on expressivity of causative variants associated with DSD. Another approach to identify variants possibly contributing to oligogenic patterns, would be the use of newer, emerging technologies such as long-read sequencing (LRS) or optical genome mapping (OGM), which can identify non-coding variants, and large or complex structural variants that can also cause DSD.^7^^,^^8^^,^^104^^,^^118^^,^^123^ Furthermore, DNA methylation and episignature analysis are increasingly being used to assist in the diagnosis of unsolved diseases and could serve as an additional tool for identifying or clarifying causative variants in complex DSD, such as those linked to *NR5A1*.^7^^,^^124^, ^125^, ^126^ Finally, rareness of the DSD phenotype and specifically persons carrying *NR5A1*/SF-1 variants also hinders statistical approaches for small sample size.

In conclusion, our study shows that approximately three out of four 46,XY DSD individuals with *NR5A1*/SF-1 variants carry additional variants in DSD-related genes that may contribute to the DSD phenotype. These findings possibly explain the broad variability of phenotypes observed with SF-1 related DSD and indicate oligogenic inheritance. Using NGS, resequencing and bioinformatic tools with disease-tailored algorithms for data analysis may yield a molecular genetic diagnosis in a larger number of DSD persons. Uncovering the genetic basis of the DSD phenotype in many individuals and their families might be more complex than assumed.

## Contributors

All authors read and approved the final version of the manuscript.

C.K.–Setup and organisation of data collection. Data collection and assistance to clinical collaborators. Access and verification of data. Data analysis, interpretation, and presentation. Creation of tables and figures. Manuscript writing. Email: chrysanthi.kouri@students.unibe.ch. ORCID 0000-0002-7225-593X

I.M.dLP.–Genetic data analysis and interpretation. Manuscript reviewing and proofreading. Email: idoia.martinezdelapiscina@unibe.ch. ORCID 0000-0001-8943-1429.

R.N.E.–Discussion of data interpretation and presentation. Manuscript reviewing and proofreading. Email: rawda.naamneh@students.unibe.ch. ORCID 0000-0003-2669-4170.

G.S. Discussion of data interpretation and presentation. Manuscript reviewing and proofreading. Email: gritti.sommer@unibe.ch. ORCID 0000-0002-4205-7932.

SF1next study group. Collaborators providing clinical, genetic data and DNA samples of patients included in the SF1next cohort. Manuscript reading and final content approval.

C.E.F. Study PI. Grant holder. Overall idea and design, organisation of study. Access and verification of data. Data analysis, interpretation and presentation. Creation of tables and figures. Manuscript writing and content responsibility and responsible for the decision to submit the manuscript. Fully employed in the academic field at University of Bern, Switzerland. Corresponding author. christa.flueck@unibe.ch. ORCID 0000-0002-4568-5504.

## Data sharing statement

Data were collected in a project-specific REDCap database governed by the Clinical Trials Unit (CTU) at University of Bern, Switzerland. Genetic data are also stored on servers of the University of Bern. These data can also be accessed upon reasonable request, according to the ethical approval and informed consent, by contacting the principal investigator of the study, who is also the corresponding author (C.E.F).

## Declaration of interests

The SF1next study group was formed from the I-DSD research community (https://sdmregistries.org/) and related networks caring for rare patients with DSD. No conflict of interest has been reported from collaborating members.

## Appendix ASupplementary data

List of names of SF1next Study Group Members

## References

[1] Hughes I.A., Houk C., Ahmed S.F., Lee P.A. (2006). Consensus statement on management of intersex disorders. J Pediatr Urol.

[2] Ostrer H. (2014). Disorders of sex development (DSDs): an update. J Clin Endocrinol Metab.

[3] Audi L., Ahmed S.F., Krone N. (2018). Genetics in endocrinology: approaches to molecular genetic diagnosis in the management of differences/disorders of sex development (DSD): position paper of EU COST Action BM 1303 ‘DSDnet’. Eur J Endocrinol.

[4] Baetens D., Mladenov W., Delle Chiaie B. (2014). Extensive clinical, hormonal and genetic screening in a large consecutive series of 46,XY neonates and infants with atypical sexual development. Orphanet J Rare Dis.

[5] Alhomaidah D., McGowan R., Ahmed S.F. (2017). The current state of diagnostic genetics for conditions affecting sex development. Clin Genet.

[6] Délot E.C., Papp J.C., Workgroup D.-T.G., Sandberg D.E., Vilain E. (2017). Genetics of disorders of sex development: the DSD-TRN experience. Endocrinol Metab Clin N Am.

[7] Délot E.C., Vilain E. (2021). Towards improved genetic diagnosis of human differences of sex development. Nat Rev Genet.

[8] Baetens D., Mendonça B.B., Verdin H., Cools M., De Baere E. (2017). Non-coding variation in disorders of sex development. Clin Genet.

[9] Gomes N.L., Chetty T., Jorgensen A., Mitchell R.T. (2020). Disorders of sex development—novel regulators, impacts on fertility, and options for fertility preservation. Int J Mol Sci.

[10] Boucekkine C., Toublanc J.E., Abbas N. (2008). The sole presence of the testis-determining region of the Y chromosome (SRY) in 46,XX patients is associated with phenotypic variability. Horm Res.

[11] Isidor B., Capito C., Paris F. (2009). Familial frameshift SRY mutation inherited from a mosaic father with testicular dysgenesis syndrome. J Clin Endocrinol Metab.

[12] Ho S.S., Urban A.E., Mills R.E. (2020). Structural variation in the sequencing era. Nat Rev Genet.

[13] Hornig N.C., de Beaufort C., Denzer F. (2016). A recurrent germline mutation in the 5’UTR of the androgen receptor causes complete androgen insensitivity by activating aberrant uORF translation. PLoS One.

[14] Gimelli G., Giorda R., Beri S., Gimelli S., Zuffardi O. (2006). A 46,X,inv(Y) young woman with gonadal dysgenesis and gonadoblastoma: cytogenetics, molecular, and methylation studies. Am J Med Genet A.

[15] Zidoune H., Ladjouze A., Chellat-Rezgoune D. (2022). Novel genomic variants, atypical phenotypes and evidence of a digenic/oligogenic contribution to disorders/differences of sex development in a large north African cohort. Front Genet.

[16] Flück C.E., Audí L., Fernández-Cancio M. (2019). Broad phenotypes of disorders/differences of sex development in MAMLD1 patients through oligogenic disease. Front Genet.

[17] Lindstrand A., Frangakis S., Carvalho C.M. (2016). Copy-number variation contributes to the mutational load of bardet-biedl syndrome. Am J Hum Genet.

[18] Cangiano B., Swee D.S., Quinton R., Bonomi M. (2021). Genetics of congenital hypogonadotropic hypogonadism: peculiarities and phenotype of an oligogenic disease. Hum Genet.

[19] Gach A., Pinkier I., Wysocka U. (2022). New findings in oligogenic inheritance of congenital hypogonadotropic hypogonadism. Arch Med Sci.

[20] Shekari S., Stankovic S., Gardner E.J. (2023). Penetrance of pathogenic genetic variants associated with premature ovarian insufficiency. Nat Med.

[21] Luo W., Ke H., Tang S. (2023). Next-generation sequencing of 500 POI patients identified novel responsible monogenic and oligogenic variants. J Ovarian Res.

[22] de Filippis T., Gelmini G., Paraboschi E. (2017). A frequent oligogenic involvement in congenital hypothyroidism. Hum Mol Genet.

[23] Sykiotis G.P., Plummer L., Hughes V.A. (2010). Oligogenic basis of isolated gonadotropin-releasing hormone deficiency. Proc Natl Acad Sci USA.

[24] Kouri C., Sommer G., Flück C.E. (2021). Hormone research in paediatrics.

[25] Kouri C., Sommer G., Martinez de Lapiscina I. (2024). Clinical and genetic characteristics of a large international cohort of individuals with rare *NR5A1*/SF-1 variants of sex development. EBioMedicine.

[26] Camats N., Fernández-Cancio M., Audí L., Schaller A., Flück C.E. (2018). Broad phenotypes in heterozygous NR5A1 46,XY patients with a disorder of sex development: an oligogenic origin?. Eur J Hum Genet.

[27] Camats N., Pandey A.V., Fernández-Cancio M. (2012). Ten novel mutations in the NR5A1 gene cause disordered sex development in 46,XY and ovarian insufficiency in 46,XX individuals. J Clin Endocrinol Metab.

[28] Fabbri-Scallet H., de Sousa L.M., Maciel-Guerra A.T., Guerra-Junior G., de Mello M.P. (2020). Mutation update for the NR5A1 gene involved in DSD and infertility. Hum Mutat.

[29] Ferraz-de-Souza B., Lin L., Achermann J.C. (2011). Steroidogenic factor-1 (SF-1, NR5A1) and human disease. Mol Cell Endocrinol.

[30] Köhler B., Lin L., Ferraz-de-Souza B. (2008). Five novel mutations in steroidogenic factor 1 (SF1, NR5A1) in 46,XY patients with severe underandrogenization but without adrenal insufficiency. Hum Mutat.

[31] Knarston I.M., Robevska G., van den Bergen J.A. (2019). NR5A1 gene variants repress the ovarian-specific WNT signaling pathway in 46,XX disorders of sex development patients. Hum Mutat.

[32] Philibert P., Zenaty D., Lin L. (2007). Mutational analysis of steroidogenic factor 1 (NR5a1) in 24 boys with bilateral anorchia: a French collaborative study. Hum Reprod.

[33] Fabbri-Scallet H., de Mello M.P., Guerra-Júnior G. (2018). Functional characterization of five NR5A1 gene mutations found in patients with 46,XY disorders of sex development. Hum Mutat.

[34] Renaux A., Papadimitriou S., Versbraegen N. (2019). ORVAL: a novel platform for the prediction and exploration of disease-causing oligogenic variant combinations. Nucleic Acids Res.

[35] Gravel B., Renaux A., Papadimitriou S., Smits G., Nowé A., Lenaerts T. (2024). Prioritization of oligogenic variant combinations in whole exomes. Bioinformatics.

[36] Papadimitriou S., Gravel B., Nachtegael C. (2023). Toward reporting standards for the pathogenicity of variant combinations involved in multilocus/oligogenic diseases. HGG Adv.

[37] Yang H., Wang K. (2015). Genomic variant annotation and prioritization with ANNOVAR and wANNOVAR. Nat Protoc.

[38] Martinez de Lapiscina I., Kouri C., Aurrekoetxea J. (2023). Genetic reanalysis of patients with a difference of sex development carrying the NR5A1/SF-1 variant p.Gly146Ala has discovered other likely disease-causing variations. PLoS One.

[39] Kopanos C., Tsiolkas V., Kouris A. (2019). VarSome: the human genomic variant search engine. Bioinformatics.

[40] Genoox Franklin by genoox. https://franklin.genoox.com.

[41] Rentzsch P., Witten D., Cooper G.M., Shendure J., Kircher M. (2019). CADD: predicting the deleteriousness of variants throughout the human genome. Nucleic Acids Res.

[42] Richards S., Aziz N., Bale S. (2015). Standards and guidelines for the interpretation of sequence variants: a joint consensus recommendation of the American college of medical genetics and Genomics and the association for molecular pathology. Genet Med.

[43] Mkaouar R., Abdallah L.C.B., Naouali C. (2021). Oligogenic inheritance underlying incomplete penetrance of PROKR2 mutations in hypogonadotropic hypogonadism. Front Genet.

[44] Jiao X., Ke H., Qin Y., Chen Z.-J. (2018). Molecular genetics of premature ovarian insufficiency. Trends Endocrinol Metab.

[45] Perea-Romero I., Solarat C., Blanco-Kelly F. (2022). Allelic overload and its clinical modifier effect in Bardet-Biedl syndrome. NPJ Genom Med.

[46] Papadimitriou S., Gazzo A., Versbraegen N. (2019). Predicting disease-causing variant combinations. Proc Natl Acad Sci USA.

[47] Versbraegen N., Gravel B., Nachtegael C. (2023). Faster and more accurate pathogenic combination predictions with VarCoPP2.0. BMC Bioinf.

[48] Versbraegen N., Fouché A., Nachtegael C. (2019). Using game theory and decision decomposition to effectively discern and characterise bi-locus diseases. Artif Intell Med.

[49] Gazzo A., Raimondi D., Daneels D. (2017). Understanding mutational effects in digenic diseases. Nucleic Acids Res.

[50] Perera E.M., Martin H., Seeherunvong T. (2001). Tescalcin, a novel gene encoding a putative EF-hand Ca(2+)-binding protein, Col9a3, and renin are expressed in the mouse testis during the early stages of gonadal differentiation. Endocrinology.

[51] Beverdam A., Koopman P. (2005). Expression profiling of purified mouse gonadal somatic cells during the critical time window of sex determination reveals novel candidate genes for human sexual dysgenesis syndromes. Hum Mol Genet.

[52] McElreavey K., Jorgensen A., Eozenou C. (2020). Pathogenic variants in the DEAH-box RNA helicase DHX37 are a frequent cause of 46,XY gonadal dysgenesis and 46,XY testicular regression syndrome. Genet Med.

[53] Zidoune H., Martinerie L., Tan D.S. (2021). Expanding DSD phenotypes associated with variants in the DEAH-box RNA helicase DHX37. Sex Dev.

[54] Tack L.J.W., Brachet C., Beauloye V. (2023). Etiology, histology, and long-term outcome of bilateral testicular regression: a large Belgian series. Hum Reprod Open.

[55] de Oliveira F.R., Mazzola T.N., de Mello M.P. (2023). DHX37 and NR5A1 variants identified in patients with 46,XY partial gonadal dysgenesis. Life.

[56] Kircher M., Witten D.M., Jain P., O’Roak B.J., Cooper G.M., Shendure J. (2014). A general framework for estimating the relative pathogenicity of human genetic variants. Nat Genet.

[57] Kothandapani A., Lewis S.R., Noel J.L. (2020). GLI3 resides at the intersection of hedgehog and androgen action to promote male sex differentiation. PLoS Genet.

[58] Carmichael S.L., Ma C., Choudhry S., Lammer E.J., Witte J.S., Shaw G.M. (2013). Hypospadias and genes related to genital tubercle and early urethral development. J Urol.

[59] Brauner R., Bignon-Topalovic J., Bashamboo A., McElreavey K. (2020). Pituitary stalk interruption syndrome is characterized by genetic heterogeneity. PLoS One.

[60] Narumi Y., Kosho T., Tsuruta G. (2010). Genital abnormalities in Pallister-Hall syndrome: report of two patients and review of the literature. Am J Med Genet A.

[61] Quaynor S.D., Bosley M.E., Duckworth C.G. (2016). Targeted next generation sequencing approach identifies eighteen new candidate genes in normosmic hypogonadotropic hypogonadism and Kallmann syndrome. Mol Cell Endocrinol.

[62] Sanjad S.A., Sakati N.A., Abu-Osba Y.K., Kaddoura R., Milner R.D. (1991). A new syndrome of congenital hypoparathyroidism, severe growth failure, and dysmorphic features. Arch Dis Child.

[63] Padidela R., Kelberman D., Press M., Al-Khawari M., Hindmarsh P.C., Dattani M.T. (2009). Mutation in the TBCE gene is associated with hypoparathyroidism-retardation-dysmorphism syndrome featuring pituitary hormone deficiencies and hypoplasia of the anterior pituitary and the corpus callosum. J Clin Endocrinol Metab.

[64] Globa E., Zelinska N., Shcherbak Y., Bignon-Topalovic J., Bashamboo A., MсElreavey K. (2022). Disorders of sex development in a large Ukrainian cohort: clinical diversity and genetic findings. Front Endocrinol.

[65] Upadhyay K., Loke J., O V., Taragin B., Ostrer H. (2018). Biallelic mutations in FLNB cause a skeletal dysplasia with 46,XY gonadal dysgenesis by activating β-catenin. Clin Genet.

[66] Foresta C., Zuccarello D., Garolla A., Ferlin A. (2008). Role of hormones, genes, and environment in human cryptorchidism. Endocr Rev.

[67] Brennan J., Tilmann C., Capel B. (2003). Pdgfr-alpha mediates testis cord organization and fetal Leydig cell development in the XY gonad. Genes Dev.

[68] Qian C., Wu Z., Ng R.C. (2019). Conditional deletion of platelet derived growth factor receptor alpha (Pdgfra) in urorectal mesenchyme causes mesenchyme apoptosis and urorectal developmental anomalies in mice. Cell Death Differ.

[69] Shannon P., Markiel A., Ozier O. (2003). Cytoscape: a software environment for integrated models of biomolecular interaction networks. Genome Res.

[70] Sreenivasan R., Ludbrook L., Fisher B. (2018). Mutant NR5A1/SF-1 in patients with disorders of sex development shows defective activation of the SOX9 TESCO enhancer. Hum Mutat.

[71] Malikova J., Camats N., Fernández-Cancio M. (2014). Human NR5A1/SF-1 mutations show decreased activity on BDNF (brain-derived neurotrophic factor), an important regulator of energy balance: testing impact of novel SF-1 mutations beyond steroidogenesis. PLoS One.

[72] Allali S., Muller J.-B., Brauner R. (2011). Mutation analysis of NR5A1 encoding steroidogenic factor 1 in 77 patients with 46, XY disorders of sex development (DSD) including hypospadias. PLoS One.

[73] Mazen I., Abdel-Hamid M., Mekkawy M. (2016). Identification of NR5A1 mutations and possible digenic inheritance in 46,XY gonadal dysgenesis. Sex Dev.

[74] Gonen N., Eozenou C., Mitter R. (2023). In vitro cellular reprogramming to model gonad development and its disorders. Sci Adv.

[75] Loke J., Pearlman A., Radi O. (2014). Mutations in MAP3K1 tilt the balance from SOX9/FGF9 to WNT/β-catenin signaling. Hum Mol Genet.

[76] Zhou B., Tang T., Chen P. (2015). The variations in the AXIN1 gene and susceptibility to cryptorchidism. J Pediatr Urol.

[77] Guo C., Sun Y., Guo C., MacDonald B.T., Borer J.G., Li X. (2014). Dkk1 in the peri-cloaca mesenchyme regulates formation of anorectal and genitourinary tracts. Dev Biol.

[78] van de Putte R., Wijers C.H., de Blaauw I. (2015). Sequencing of the DKK1 gene in patients with anorectal malformations and hypospadias. Eur J Pediatr.

[79] Naamneh Elzenaty R., Martinez de Lapiscina I., Kouri C. (2024). Characterization of 35 novel NR5A1/SF-1 variants identified in individuals with atypical sexual development: the SF1next study. J Clin Endocrinol Metab.

[80] Liu B., Chen S.C., Yang Y.M. (2015). Identification of novel PKD1 and PKD2 mutations in a Chinese population with autosomal dominant polycystic kidney disease. Sci Rep.

[81] Tsai M.C., Weng Y.H., Lin Y.F. (2023). Whole-exome sequencing identified rare genetic variants associated with undervirilized genitalia in Taiwanese pediatric patients. Biomedicines.

[82] Zlotina A., Kiselev A., Sergushichev A., Parmon E., Kostareva A. (2018). Rare case of ulnar-mammary-like syndrome with left ventricular tachycardia and lack of TBX3 mutation. Front Genet.

[83] Zhang X., Chen L., Li L. (2023). Literature review, report, and analysis of genotype and clinical phenotype of a rare case of ulnar-mammary syndrome. Front Pediatr.

[84] Miraoui H., Dwyer A.A., Sykiotis G.P. (2013). Mutations in FGF17, IL17RD, DUSP6, SPRY4, and FLRT3 are identified in individuals with congenital hypogonadotropic hypogonadism. Am J Hum Genet.

[85] Men M., Wang X., Wu J. (2021). Prevalence and associated phenotypes of DUSP6, IL17RD and SPRY4 variants in a large Chinese cohort with isolated hypogonadotropic hypogonadism. J Med Genet.

[86] Indirli R., Cangiano B., Profka E. (2019). A rare SPRY4 gene mutation is associated with anosmia and adult-onset isolated hypogonadotropic hypogonadism. Front Endocrinol.

[87] Hart D., Rodriguez Gutierrez D., Lauber-Biason A. (2022). CBX2 in DSD: the quirky kid on the block. Sex Dev.

[88] Doghman M., Figueiredo B.C., Volante M., Papotti M., Lalli E. (2013). Integrative analysis of SF-1 transcription factor dosage impact on genome-wide binding and gene expression regulation. Nucleic Acids Res.

[89] Biason-Lauber A., Konrad D., Meyer M., DeBeaufort C., Schoenle E.J. (2009). Ovaries and female phenotype in a girl with 46,XY karyotype and mutations in the CBX2 gene. Am J Hum Genet.

[90] Martínez de LaPiscina I., Mahmoud R.A., Sauter K.S. (2020). Variants of STAR, AMH and ZFPM2/FOG2 may contribute towards the broad phenotype observed in 46,XY DSD patients with heterozygous variants of NR5A1. Int J Mol Sci.

[91] Calonga-Solís V., Fabbri-Scallet H., Ott F. (2022). MYRF: a new regulator of cardiac and early gonadal development-insights from single cell RNA sequencing analysis. J Clin Med.

[92] Buaas F.W., Val P., Swain A. (2009). The transcription co-factor CITED2 functions during sex determination and early gonad development. Hum Mol Genet.

[93] Ferraz-de-Souza B., Lin L., Shah S. (2011). ChIP-on-chip analysis reveals angiopoietin 2 (Ang2, ANGPT2) as a novel target of steroidogenic factor-1 (SF-1, NR5A1) in the human adrenal gland. FASEB J.

[94] Combes A.N., Spiller C.M., Harley V.R. (2010). Gonadal defects in Cited2-mutant mice indicate a role for SF1 in both testis and ovary differentiation. Int J Dev Biol.

[95] Fonseca D.J., Ojeda D., Lakhal B. (2012). CITED2 mutations potentially cause idiopathic premature ovarian failure. Transl Res.

[96] Eggers S., Sadedin S., van den Bergen J.A. (2016). Disorders of sex development: insights from targeted gene sequencing of a large international patient cohort. Genome Biol.

[97] Werner R., Mönig I., Lünstedt R. (2017). New NR5A1 mutations and phenotypic variations of gonadal dysgenesis. PLoS One.

[98] Wang W., Zhang C., Marimuthu A. (2005). The crystal structures of human steroidogenic factor-1 and liver receptor homologue-1. Proc Natl Acad Sci U S A.

[99] Hughes L.A., McKay-Bounford K., Webb E.A. (2019). Next generation sequencing (NGS) to improve the diagnosis and management of patients with disorders of sex development (DSD). Endocr Connect.

[100] Robevska G., van den Bergen J.A., Ohnesorg T. (2018). Functional characterization of novel NR5A1 variants reveals multiple complex roles in disorders of sex development. Hum Mutat.

[101] Eggers S., Smith K.R., Bahlo M. (2015). Whole exome sequencing combined with linkage analysis identifies a novel 3 bp deletion in NR5A1. Eur J Hum Genet.

[102] Sreenivasan R., Bell K., van den Bergen J. (2022). Whole exome sequencing reveals copy number variants in individuals with disorders of sex development. Mol Cell Endocrinol.

[103] Mazen I., Mekkawy M., Kamel A. (2021). Advances in genomic diagnosis of a large cohort of Egyptian patients with disorders of sex development. Am J Med Genet A.

[104] Fabbri-Scallet H., Werner R., Guaragna M.S. (2022). Can non-coding NR5A1 gene variants explain phenotypes of disorders of sex development?. Sex Dev.

[105] Cheng Y., Chen J., Zhou X., Yang J., Ji Y., Xu C. (2021). Characteristics and possible mechanisms of 46, XY differences in sex development caused by novel compound variants in NR5A1 and MAP3K1. Orphanet J Rare Dis.

[106] Laan M., Kasak L., Timinskas K. (2021). NR5A1 c.991-1G > C splice-site variant causes familial 46,XY partial gonadal dysgenesis with incomplete penetrance. Clin Endocrinol.

[107] Cannarella R., Condorelli R.A., Paolacci S. (2021). Next-generation sequencing: toward an increase in the diagnostic yield in patients with apparently idiopathic spermatogenic failure. Asian J Androl.

[108] Giannakopoulos A., Sertedaki A., Chrysis D. (2022). A human paradigm of LHX4 and NR5A1 developmental gene interaction in the pituitary gland and ovary?. Eur J Hum Genet.

[109] Gomes N.L., Batista R.L., Nishi M.Y. (2022). Contribution of clinical and genetic approaches for diagnosing 209 index cases with 46,XY differences of sex development. J Clin Endocrinol Metab.

[110] Oral E., Toksoy G., Sofiyeva N. (2019). Clinical and genetic investigation of premature ovarian insufficiency cases from Turkey. J Gynecol Obstet Hum Reprod.

[111] Wang N., Zhu W., Han B. (2020). Inherited missense mutation occurring in Arginine76 of the SRY gene does not account for familial 46, XY sex reversal. J Clin Endocrinol Metab.

[112] Li L., Gao F., Fan L., Su C., Liang X., Gong C. (2020). Disorders of sex development in individuals harbouring MAMLD1 variants: WES and interactome evidence of oligogenic inheritance. Front Endocrinol.

[113] Wang H., Zhang L., Wang N. (2018). Next-generation sequencing reveals genetic landscape in 46, XY disorders of sexual development patients with variable phenotypes. Hum Genet.

[114] Kousi M., Katsanis N. (2015). Genetic modifiers and oligogenic inheritance. Cold Spring Harb Perspect Med.

[115] Cordell H.J. (2002). Epistasis: what it means, what it doesn’t mean, and statistical methods to detect it in humans. Hum Mol Genet.

[116] Rodríguez Gutiérrez D., Eid W., Biason-Lauber A. (2018). A human gonadal cell model from induced pluripotent stem cells. Front Genet.

[117] Ruiz-Babot G., Balyura M., Hadjidemetriou I. (2018). Modeling congenital adrenal hyperplasia and testing interventions for adrenal insufficiency using donor-specific reprogrammed cells. Cell Rep.

[118] Houzelstein D., Eozenou C., Lagos C.F. (2024). A conserved NR5A1-responsive enhancer regulates SRY in testis-determination. Nat Commun.

[119] Achermann J.C. (2024). Steroidogenic factor-1 (NR5A1): orphan nuclear receptor finds a home in human reproduction, and beyond. EBioMedicine.

[120] García-Acero M., Moreno O., Suárez F., Rojas A. (2020). Disorders of sexual development: current status and progress in the diagnostic approach. Curr Urol.

[121] Long P., Wang L., Tan H. (2024). Oligogenic basis of premature ovarian insufficiency: an observational study. J Ovarian Res.

[122] Zhao T., Ma Y., Zhang Z. (2021). Young and early-onset dilated cardiomyopathy with malignant ventricular arrhythmia and sudden cardiac death induced by the heterozygous LDB3, MYH6, and SYNE1 missense mutations. Ann Noninvasive Electrocardiol.

[123] Croft B., Ohnesorg T., Sinclair A.H. (2018). The role of copy number variants in disorders of sex development. Sex Dev.

[124] Aref-Eshghi E., Bend E.G., Colaiacovo S. (2019). Diagnostic utility of genome-wide DNA methylation testing in genetically unsolved individuals with suspected hereditary conditions. Am J Hum Genet.

[125] Sadikovic B., Levy M.A., Kerkhof J. (2021). Clinical epigenomics: genome-wide DNA methylation analysis for the diagnosis of Mendelian disorders. Genet Med.

[126] LaFlamme C.W., Rastin C., Sengupta S. (2024). Diagnostic utility of DNA methylation analysis in genetically unsolved pediatric epilepsies and CHD2 episignature refinement. Nat Commun.

