# Supplementary material for: Oligogenic analysis across broad phenotypes of 46,XY differences in sex development associated with NR5A1/SF-1 variants: findings from the international SF1next study
Source: eBioMedicine. 2025 Mar 3;113:105624. doi: 10.1016/j.ebiom.2025.105624 (PMC11925193; doi:10.1016/j.ebiom.2025.105624)
Supplement: List of names of SF1next Study Group Members [file mmc1.docx]

**Oligogenicity may explain the broad phenotype of 46,XY differences in sex development associated with *NR5A1*/SF-1 variants: Findings from the international SF1next study**

**Chrysanthi Kouri^1,2,3^, Idoia Martinez de Lapiscina ^1,2,4,5,6,7^, Rawda Naamneh Elzenaty^1,2,3^, Grit Sommer^1,8^, Kay-Sara Sauter^1,2^, Christa E Flück^1,2^, SF1next study group^#^**

| **List of SF1next Study Group Members** | |  |  |
| --- | --- | --- | --- |
|  |  |  |  |
| **First name** | **Last name** |  |  |
| Saygin | Abali |  |  |
| Zehra | Yavas Abali |  |  |
| S. Faisal | Ahmed |  |  |
| Leyla | Akin |  |  |
| Maricruz | Almaraz |  |  |
| Laura | Audí |  |  |
| Murat | Aydin |  |  |
| Antonio | Balsamo |  |  |
| Federico | Baronio |  |  |
| Jillian | Bryce |  |  |
| Kanetee | Busiah |  |  |
| Maria | Caimari |  |  |
| Núria | Camats-Tarruella |  |  |
| Ariadna | Campos-Martorell |  |  |
| Luis | Castaño |  |  |
| Anna | Casteràs |  |  |
| Semra | Çetinkaya |  |  |
| Hedi L | Claahsen - van der Grinten | |  |
| Martine | Cools | |  |
| Ines | Costa |  |  |
| Fatma Feyza | Darendeliler |  |  |
| Justin H | Davies |  |  |
| Isabel | Esteva |  |  |
| Helena | Fabbri-Scallet |  |  |
| Courtney A | Finlayson |  |  |
| Emilio | Garcia |  |  |
| Beatriz | Garcia- Cuartero |  |  |
| Alina | German |  |  |
| Evgenia | Globa |  |  |
| Gil | Guerra-Junior |  |  |
| Julio | Guerrero |  |  |
| Tulay | Guran |  |  |
| Sabine E | Hannema |  |  |
| Olaf | Hiort |  |  |
| Josephine | Hirsch |  |  |
| Ieuan | Hughes |  |  |
| Marco | Janner |  |  |
| Uchenna | Kennedy |  |  |
| Zofia | Kolesinska |  |  |
| Katherine | Lachlan |  |  |
| Anna | Lauber-Biason |  |  |
| Jana | Krenek Malikova |  |  |
| Dagmar | l'Allemand |  |  |
| Nina | Lenhnerr-Taube |  |  |
| Angela | Lucas-Herald |  |  |
| Jamala | Mammadova |  |  |
| Veronica | Mericq |  |  |
| Isabel | Mönig |  |  |
| Francisca | Moreno |  |  |
| Julia | Mührer |  |  |
| Marek | Niedziela |  |  |
| Anna | Nordenstrom |  |  |
| Burçe | Orman |  |  |
| Sukran | Poyrazoglu |  |  |
| Jose M | Rial |  |  |
| Meilan M | Rutter |  |  |
| Amaia | Rodríguez |  |  |
| Tara | Schafer-Kalkhoff |  |  |
| Sumudu Nimali | Seneviratne |  |  |
| Maria | Sredkova-Ruskova |  |  |
| LIoyd J. W | Tack |  |  |
| Rieko | Tadokoro-Cuccaro |  |  |
| Ajay | Thankamony |  |  |
| Mónica | Tomé |  |  |
| Amaia | Vela |  |  |
| Malgorzata | Wasniewska |  |  |
| David | Zangen |  |  |
| Nataliya | Zelinska |  |  |
