# Supplementary material for: Oligogenic analysis across broad phenotypes of 46,XY differences in sex development associated with NR5A1/SF-1 variants: findings from the international SF1next study
Source: eBioMedicine. 2025 Mar 3;113:105624. doi: 10.1016/j.ebiom.2025.105624 (PMC11925193; doi:10.1016/j.ebiom.2025.105624)
Supplement: Supplementary Figs. S1 and S2 and Tables S1–S4 [file mmc2.pdf]

Supplementary Material to: Oligogenic analysis across broad phenotypes of 46,XY differences in sex development associated with *NR5A1*/SF-1 variants: findings from the international SF1next study

Table of content

Figure S1: Flowchart of participant recruitment and study analysis. 2

Table S1: Additional gene hits associations with DSD and/or SF-1 in the reported literature. 3

Table S2: Comprehensive clinical and genetic characteristics of DSD index cases with *NR5A1*/SF-1 and additional gene variants suggesting oligogenicity according to ORVAL[114] and their family members. 5

Table S3: Gene variant characterisation and *in silico* analysis of 22 DSD cases with predicted oligogenic variant combinations.. 15

Figure S2: Density plot representing the PHRED-scaled CADD scores of 64 variants identified in 22 index cases (plus their family members) with predicted combinations involving *NR5A1*/SF-1 variants. 19

Table S4: List of additional variants per case, discarded after filtering due to lack of association with the observed phenotype of the individuals. 20

References to supplementary material 24

Figure S1: Flowchart of participant recruitment and study analysis.

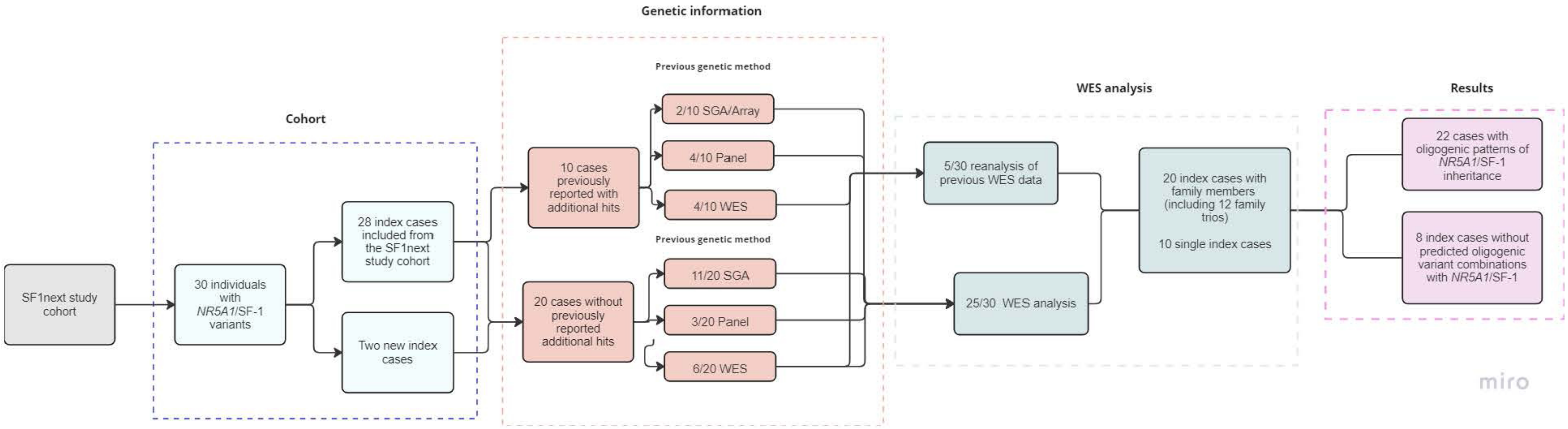

SGA, single gene analysis; WES, Whole exome sequencing

Table S1: Additional gene hits associations with DSD and/or SF-1 in the reported literature.

Gene **(OMIM)**	**Relation with DSD/SF-1**
AKR1C3 (603966)	DSD related gene<br>Hypospadias[1]<br>Testicular descent[2]
APC (611731)	SF-1 related gene<br>Negative regulator of Wnt signalling,<br>interaction with SF-1, regression of Müllerian ducts[3]
AXIN1 (603816)	DSD related gene<br>Male sex development[4,5]<br>Cryptorchidism[6]
CBX2 (602770)	DSD and SF-1 related gene<br>SF-1 binding sites[7]<br>DSD, sex reversal, gonadal development[8,9]
CCDC59 (619280)	DSD related gene<br>Hypospadias (?)
CDH23 (605516)	DSD related gene<br>Oligogenic 46,XY DSD[10-12]<br>Gonadal development[13]
CITED2 (602937)	DSD and SF-1 related gene<br>Gonadal development [14-16]<br>Interaction with SF-1 for testis and ovary differentiation[17]
COL1A1 (120150)	DSD related gene<br>Hypospadias[18]<br>Hypogonadism[19]
COL9A3	DSD related gene<br>Male sex development[20,21]
DHRS7 (612833)	DSD related gene<br>Androgen metabolism[22,23]
DKK1 (605189)	DSD related gene<br>Formation of anorectal and genitourinary tracts[24]<br>Anorectal malformations and hypospadias[25]
DHX37 (617362)	DSD related gene<br>46,XY gonadal dysgenesis[26,27]<br>Testicular regression[26,27]<br>Oligogenic 46,XY DSD[28]
DMRT2 (604935)	DSD related gene (?)<br>46,XY DSD, sex reversal[29,30]<br>46,XY OTDSD[31]
DOCK8 (611432)	DSD related gene (?)<br>Oligogenic in 46,XY DSD with *NR5A1* variant[32]<br>46,XY DSD gonadal dysgenesis[33,34]
ETNK2 (609859)	DSD and SF-1 related gene<br>SF-1 binding sites[35]<br>Sex determination (testis) in mice[36,37]
FBLN2 (135821)	DSD related gene (?)<br>46,XY DSD[38,39]
FDXR (103270)	SF-1 related gene<br>Transcriptional regulation by SF-1[40]<br>Ambiguous genitalia[41]
FLNB (603381)	DSD related gene<br>46,XY DSD[42]<br>Larsen syndrome; cryptorchidism[43]
GATA5 (611496)	DSD related gene (?)<br>Genital anomalies[44]<br>Genitourinary abnormalities in mice[45]
GLI2 (165230)	DSD and SF-1 related gene<br>Oligogenic 46,XY DSD[11,46]<br>Cryptorchidism[47]<br>Hypospadias[48]
GLI3 (165240)	DSD related gene<br>Male sex differentiation[49]<br>Hypospadias,[48] cryptorchidism,[50] HH[51]<br>Oligogenic 46,XY DSD[46]
HHAT (605743)	DSD related gene (?)<br>46,XY DSD[52-55]
INO80 (610169)	DSD related gene<br>Urethral development interaction with BMP4[56,57]
KANK1 (607704)	DSD related gene<br>Embryonic genital development, interaction with β-catenin[58,59]<br>46,XY DSD gonadal dysgenesis[60]
KAT6B (605880)	DSD related gene

	Oligogenic 46,XY DSD[46]<br>Syndromic DSD[61,62]
LGR5 (606667)	DSD related gene (?)<br>46,XY DSD, gonadal dysgenesis, micropenis[38]<br>Regulator of Wnt signaling with R-spondin[63]
LRP6 (606667)	DSD related gene<br>Co-receptor of Wnt/β-catenin pathway in gonadal differentiation[64]<br>Germ cell differentiation[65]
MAPK14 (600289)	DSD related gene<br>Testis development[66]<br>Sex reversal in mice[67]<br>Hypospadias in rats[68]
MKKS (604896)	DSD related gene<br>Bardet-Biedl syndrome and McKusick-Kaufman syndrome (DSD features) [69-71]
MYO7A (276903)	DSD related gene<br>Gonadal development[72]<br>Target gene of SRY and SOX9 genes[73]<br>Oligogenic 46,XY DSD[74,75] and 46,XX POF[10]
NOS1 (163731)	SF-1 related gene<br>SF-1 binding regions[35]<br>HH, micropenis, cryptorchidism[76]
NR1H2 (600380)	SF-1 related gene<br>SF-1 expression[77]<br>Male infertility[78,79]<br>Hypogonadism, testicular physiology, testosterone synthesis in mice[80] Cholesterol homeostasis in testis in mice[81]
PDGFRA (173490)	DSD related gene<br>Testis development[82]<br>Anorectal malformations, hypospadias[83]
PKD1 (601313)	DSD related gene<br>46,XY DSD[75,84]<br>Male reproductive tract development[85]
PLXNB1 (601053)	SF-1 related gene<br>SF-1 binding sites[35]<br>HH[86]<br>HH and cryptorchidism[86]
PPARGC1B (608886)	DSD related gene<br>Oligogenic 46,XY DSD[10]<br>Hypospadias[87]
PTCH1 (601309)	DSD related gene<br>HH and cryptorchidism[88]<br>Hypospadias[89]
RXFP2 (606655)	DSD related gene<br>Cryptorchidism[43,90,91]
SEMA6D (609295)	SF-1 related gene<br>SF-1 binding sites[35]<br>Reproductive function, HH [92-94]
SFRP1 (604156)	DSD related gene<br>Antagonist of Wnt signaling, male sexual development[95,96]
SPRY4 (607984)	DSD related gene<br>HH[97-99]<br>Oligogenic inheritance in HH[100]
SRA1 (603819)	DSD and SF-1 related gene<br>SF-1 interaction[101]<br>Additional hit in 46,XY DSD with *NR5A1* variant [102]<br>HH[103]
SRCAP (611421)	DSD related gene<br>Oligogenic 46,XY DSD[75]<br>46,XY DSD[104]
SYNM (606087)	DSD related gene<br>Ulnar-Mammary-Like Syndrome (DSD features) [105,106]
TBCE (604934)	DSD related gene<br>Micropenis, cryptorchidism[104,107,108]
TBX2 (600747)	DSD related gene<br>Development of reproductive system[109]<br>Oligogenic modifier in 46,XY DSD with *NR5A1* variants [110]
ZFPM2 (603693)	DSD related gene<br>46,XY sex reversal 9 (OMIM: 603693)<br>46,XY DSD, gonadal dysgenesis [111,112]
ZNF462 (617371)	DSD related gene (?)<br>Cryptorchidism[113]<br>HH[114,115]

HH, Hypogonadotropic hypogonadism; POF, Premature ovarian failure, OTDSD, Ovotesticular DSD, (?) under exploration with DSD based on initial evidence

Table S2: Comprehensive clinical and genetic characteristics of DSD index cases with *NR5A1*/SF-1 and additional gene variants suggesting oligogenicity according to ORVAL[116] and their family members.

Index case	Karyotype/ Sex of rearing	Clinical phenotype (index case, family members)	*NR5A1*/SF-1 variant (Zygosity, index case), ACMG	Family members tested (Zygosity)	Additional variants (Zygosity)	Found in	ORVAL score[#]	Digenic Effect[ç]	Clinical significance* VarSome/ Franklin	CADD
**9**	46,XY Female	External genitalia: typical female<br>Internal genitalia: hypoplastic uterus, streak gonads<br>Opposite sex - CGD<br>Mother healthy<br>Father healthy	c.902G>A; p.Cys301Tyr (Het), Likely pathogenic	Mother (Het, mosaic)<br>Father (WT)	*PKD1*: c.6598C>T; p.(Arg2200Cys) (Het)	Index & Father	0.9950	TG	B/VUS	22.9
					CITED2: c.117_119del; p.(His39del) (Het)	Index & Father	0.9800	TG	B/LB	22.0
					PDGFRA: c.1285G>A; p.(Gly429Arg) (Het)	Index & Father	0.9775	TG	LB/VUS	22.4
					FLNB: c.4233C>G; p.(Phe1411Leu) (Het)	Index & Father	0.9375	TG	B/LB	21
					FLNB: c.6017A>G; p.(Lys2006Arg) (Het)	Index	0.9375	TG	B/LB	21
					TBCE: c.214C>T; p.(Pro72Ser) (Het)	Index	0.8550	MM	B/B	23.2
**10**	46,XY Male	External genitalia: meatal opening penoscrotal, impalpable gonads<br>Internal genitalia: abnormal left testes, absent right testes, Müllerian structures present<br>Severe - NSDU	c.841C>T; p.Arg281Cys (Het), Likely pathogenic	NA	*ZFPM2*: c.292G>A; p.(Asp98Asn) (Het)	Index	0.9975	TG	B/B	23.9
					CCDC59: c.499A>G; p.(Thr167Ala) (Het)	Index	0.8075	MM	VUS/VUS	25.3
**11**	46,XY Male	External genitalia: meatal opening penoscrotal, gonads labioscrotal, genital tubercle 21-30 mm, labioscrotal fusion: unfused	c.1109G>A; p.Cys370Tyr (Het), Likely pathogenic	Mother (Het)<br>Father (WT)	*GLI3*: c.2179G>A; p.(Gly727Arg) (Het)	Index & Father	0.9850	TG	B/VUS	25.5

		Internal genitalia: testes<br>Severe - PGD<br>Mother healthy<br>Father healthy			*KANK1*: c.1322C>T;<br>p.(Thr441Ile) (Het)	Index &<br>Father	0.8425	TG	VUS/VUS	24.3
**12**	46,XY<br>Female	External genitalia: typical female<br>Internal genitalia: streak gonads<br>Opposite sex - CGD<br>Sibling (male)<br>External genitalia: meatal opening penoscrotal, gonads labioscrotal, genital tubercle <10 mm, labioscrotal fusion: fused<br>Internal genitalia: testes, rudimentary uterus.<br>Other anomalies: brachymetatarsia, long-jointed hands and feet, decreased muscle mass<br>Severe - Other DSD	c.217T>A;<br>p.Cys73Ser (Het),<br>Likely pathogenic	Mother (Het)<br>Father (WT)<br>Brother (Het)	*GLI3*: c.2179G>A;<br>p.(Gly727Arg) (Het)	Index,<br>Father &<br>Brother	0.9850	TG	B/VUS	25.5
					APC: c.7514G>A;<br>p.(Arg2505Gln) (Het)	Index,<br>Father &<br>Brother	0.9600	TG	B/B	23.8
					PKD1: c.12436G>A;<br>p.(Val4146Ile) (Het)	Index,<br>Father &<br>Brother	0.9950	TG	B/VUS	23.3
					SYNM: c.361C>A;<br>p.(Gln121Lys) (Het)	Brother	0.7025	MM	LB/VUS	19.38
					SYNM: c.368C>T;<br>p.(Ala123Val) (Het)	Brother	0.7025	MM	LB/VUS	20.5
**13**	46,XY Male	External genitalia: meatal opening scrotal, gonads labioscrotal, genital tubercle 10-20, labioscrotal fusion: posterior fusion<br>Internal genitalia: testes<br>Severe - TDSD<br>Mother subfertility (ovarian stimulation), no POI<br>Father healthy	c.40C>T;<br>p.Pro14Ser (Het),<br>VUS	Mother (Het)<br>Father (WT)	*GLI3*: c.2179G>A;<br>p.(Gly727Arg) (Het)	Index &<br>Father	0.9850	TG	B/VUS	25.5
					CBX2: c.849G>T;<br>p.(Lys283Asn) (Het)	Index &<br>Father	0.9550	TG	B/B	20.7
**14**	46,XY Male	External genitalia: meatal opening perineal, gonads inguinal, genital tubercle 21-30 mm, labioscrotal fusion: fused<br>Internal genitalia: testes, vas deferens<br>Abnormal response in HCG stimulation test at birth<br>Other anomalies: Type 1 diabetes, celiac disease<br>Severe - NSDU	c.937C>T;<br>p.Arg313Cys (Het)<br>(*de novo*),<br>Pathogenic	Mother (WT)<br>Father (WT)	*SPRY4*: c.55C>G;<br>p.(Gln19Glu) (Het)	Index	0.9300	MM	VUS/VUS	24.6
					TBCE: c.214C>T;<br>p.(Pro72Ser) (Het)	Index	0.8600	MM	B/B	23.2

					INO80: c.3842G>A; p.(Arg1281Gln) (Het)	Index & Mother	0.9450	TG	B/VUS	27.6
					FLNB: c.6956T>C; p.(Ile2319Thr) (Het)	Index & Mother	0.9400	TG	B/B	28.0
**15**	46,XY Female	External genitalia: typical female Internal genitalia: streak gonads Opposite sex - CGD	c.614_615insC; p.Gln206Thrfs*20 (Het), Pathogenic	Mother (WT)	*SPRY4*: c.653C>A; p.(Ser218Tyr) (Het)	Index	0.9275	MM	VUS/VUS	27.8
					MKKS: c.724G>T; p.(Ala242Ser) (Het)	Index	0.9175	MM	LB/VUS	24.9
					FDXR: c.815C>T; p.(Pro272Leu) (Het)	Index & Mother	0.8525	MM	VUS/VUS	27.6
**16**	46,XY Female	External genitalia: gonads inguinoscrotal and inguinal, labioscrotal fusion: fused Internal genitalia: testes Severe - TDSD	c.102+1G>T (Het) (denovo), Likely pathogenic	NA	FLNB: c.6956T>C; p.(Ile2319Thr) (Het)	Index	0.9475	TG	B/B	28.0
					KAT6B: c.5252C>A; p.(Pro1751His) (Het)	Index	0.9650	TG	VUS/VUS	24.4
					MYO7A: c.2293C>A; p.(Leu765Met) (Het)	Index	0.9000	MM	B/LB	23.2
					PKD1: c.2081C>T; p.(Pro694Leu) (Het)	Index	0.9950	TG	LB/VUS	25.4

					SEMA6D: c.626G>A; p.(Arg209His) (Het)	Index & Twin brother	0.9050	TG	LB/VUS	32
**17**	46,XY Male	External genitalia: meatal opening perineal, gonads labioscrotal, genital tubercle 21-30 mm, labioscrotal fusion: fused<br>Internal genitalia: testes<br>Severe - TDSD<br>Twin brother<br>External genitalia: meatal opening perineal, gonads inguinoscrotal, genital tubercle 21-30 mm, labioscrotal fusion: posterior fusion<br>Internal genitalia: testes<br>Mother healthy<br>Severe - TDSD	c.938G>A; p.Arg313His (Het), Pathogenic	Mother (Het)<br>Father (WT)<br>Twin brother (Het)	*PDGFRA*: c.1285G>A; p.(Gly429Arg)(Het)	Index, Twin brother & Mother	0.9750	TG	LB/VUS	16.3
					ZNF462: c.4093G>A; p.(Glu1365Lys) (Het)	Index, Twin brother & Mother	0.9825	TG	B/VUS	25.1
					DKK1: c.470G>T; p.Ser157Ile (Het)	Index	0.9400	TG	B/LB	21.7
**18**	46,XY Male	External genitalia: meatal opening penoscrotal, gonads impalpable, labioscrotal fusion: posterior fusion<br>Internal genitalia: testes, mullerian remnants<br>Severe - PGD<br>Father healthy	c.937C>T; p.Arg313Cys (Het), Pathogenic	Father (Het)<br>Mother (WT)						
					AXIN1: c.1485C>G; p.(Asp495Glu) (Het)	Index	0.9275	MM	B/VUS	17.2

**19**	46,XY Female	External genitalia: meatal opening scrotal, gonads labioscrotal, genital tubercle 10-20 mm, labioscrotal fusion: posterior fusion<br>Internal genitalia: testes<br>First brother<br>External genitalia: meatal opening penoscrotal, gonads labioscrotal, genital tubercle 10-20 mm, labioscrotal fusion: fused<br>Internal genitalia: testes<br>Second brother<br>External genitalia: meatal opening penoscrotal, gonads inguinal, genital tubercle 10-20 mm, labioscrotal fusion: fused<br>All Severe - PGD	c.194G>A; p.Cys65Tyr (Het), Likely pathogenic	Father (WT)<br>Brothers (Het)	*SFRP1*: c.539C>T; p.(Pro180Leu) (Het)	Index & Brothers	0.9725	TG	B/VUS	26.8
					COL1A1: c.1559A>G; p.(Lys520Arg) (Het)	Index & Brothers	0.8975	TG	VUS/VUS	23.1
**20**	46,XY Male	External genitalia: meatal opening penoscrotal, gonads labioscrotal, genital tubercle 10-20 mm, labioscrotal fusion: unfused<br>Internal genitalia: testes<br>Severe - PGD<br>Mother subfertility, IVF for second pregnancy<br>Father healthy	c.938G>A; p.Arg313His (Het), Pathogenic	Mother (Het)<br>Father (WT)	*LRP6*: c.4402G>A; p.(Ala1468Thr) (Het)	Index & Father	0.9900	TG	B/VUS	27.4
					ETNK2: c.920A>C; p.(Gln307Pro) (Het)	Index & Father	0.8625	TG	VUS/VUS	22.9
**21**	46,XY Male	External genitalia: meatal opening perineal, gonads labioscrotal, genital tubercle 10-20 mm, labioscrotal fusion: posterior fusion<br>Internal genitalia: testes<br>Other anomalies: hyposplenism and thrombocytosis<br>Severe - PGD	c.991-1G>A (Het) (*de novo*), Likely pathogenic	Mother (WT)<br>Father (WT)	*GLI2*: c.803C>T; p.(Ala268Val) (Het)	Index & Mother	0.9725	TG	B/B	25.0
					CDH23: c.5831T>C; p.(Leu1944Ser) (Het)	Index & Father	0.9450	TG	B/VUS	22.6
					LGR5: c.1148A>G; p.(His383Arg) (Het)	Index & Father	0.9425	MM	B/LB	22.3

					GATA5: c.232G>A; p.Gly78Ser (Het)	Index & Mother	0.8675	TG	B/LB	15.44
					PPARGC1B: c.1088C>T; p.(Thr363Met) (Het)	Index & Father	0.8625	TG	B/B	17.2
					PPARGC1B: c.1499C>T; p.(Ser500Leu) (Het)	Index & Father	0.8625	TG	B/B	10.03
**22**	46,XY Male	External genitalia: meatal opening perineal, gonads inguinal, labioscrotal fusion: fused<br>Internal genitalia: small testes<br>Severe - PGD	c.71A>T; p.His24Leu (Het), Likely pathogenic	NA	*MAPK14*: c.1028A>G; p.(Asp343Gly) (Het)	Index	0.9850	TG	B/LB	23.4
					PLXNB1: c.1360A>C; p.(Ser454Arg) (Het)	Index	0.8850	MM	B/VUS	25.7
					PTCH1: c.4324C>T; p.(Leu277Met) (Het)	Index	0.8625	TG	B/LB	20.2
					HHAT: c.829C>A; p.(Leu277Met) (Het)	Index	0.8600	MM	VUS/VUS	23.8

					HHAT: c.1130A>G; p.(Tyr377Cys) (Het)	Index	0.8600	MM	VUS/VUS	23.0
**23**	46,XY Female	External genitalia: typical female, Internal genitalia: abnormal testes, uterus Opposite sex - PGD	c.151G>T; p.Glu51* (Het), Pathogenic	NA	*SRA1*: c.413G>A ; p.(Gly138Glu) (Het)	Index	0.9625	TG	B/LB	19.2
					MYO7A: c.1868G>A; p.(Arg623His) (Het)	Index	0.9225	MM	B/B	26.8
					SRCAP: c.4499C>T; p.(Pro1500Leu) (Het)	Index	0.9100	TG	B/VUS	22.1
					SRCAP:c.4603C>G ; p.(Pro1535Ala) (Het)	Index	0.9100	TG	B/B	17.8
**24**	46,XY Female	External genitalia: meatal opening penoscrotal, gonads impalpable, genital tubercle >30 mm, labioscrotal fusion: posterior fusion Internal genitalia: testes Severe - PGD Other anomalies: slight skull dysmorphism, prominent front drafts, scoliosis, impaired psychomotor development , mild leukopenia	c.1379A>G; p.Gln460Arg (Het), VUS	NA	*TBX2*: c.1139C>G; p.(Pro380Arg) (Het)	Index	0.8875	TG	VUS/VUS	24.7
					FLNB: c.2195A>G; p.(Tyr732Cys) (Het)	Index	0.6900	TG	VUS/VUS	23.8

					NOS1: c.335C>T; p.(Thr112Ile) (Het)	Index	0.9850	TG	B/VUS	23.2
					DMRT2: c.674C>G; p.(Pro225Arg) (Het)	Index	0.9575	MM	LB/VUS	24.7
**25**	46,XY Male	External genitalia: meatal opening penoscrotal, gonads labioscrotal & inguinal, labioscrotal fusion: fused<br>Internal genitalia: testes<br>Severe - Gonadal regression	c.271G>A; p.Gly91Ser (Het), Likely pathogenic	NA	*FLNB*: c.6017A>G; p.(Lys2006Arg) (Het)	Index	0.8975	TG	B/B	21.0
					AKR1C3: c.548A>T; p.Lys183Met (Het)	Index	0.8875	MM	B/VUS	25.6
					DHRS7: c.431G>A; p.(Arg144His) (Het)	Index	0.7175	MM	VUS/VUS	26.7
**26**	46,XY Male	External genitalia: meatal opening scrotal, gonads inguinal, genital tubercle 10-20, labioscrotal fusion: fused<br>Internal genitalia: small testes<br>Other anomalies: bilateral cysts in epididymis, Asperger syndrome<br>Severe - NSDU	c.88T>A; p.Cys30Ser (Het), Likely pathogenic	NA	*DOCK8*: c.137G>A; p.(Gly46Asp) (Het)	Index	0.9125	TG	LB/VUS	22.1
					KAT6B: c.2134G>T; p.(Gly712Trp) (Het)	Index	0.9700	TG	B/VUS	28.0

					RXFP2: c.1594C>G; p.(Arg532Gly) (Het)	Index	0.8675	TG	B/VUS	20.4
**27**	46,XY Male	External genitalia: typical female<br>Internal genitalia: hypoplastic uterus, streak gonads<br>Opposite sex - CGD<br>Mother healthy<br>Father healthy	c.1183_1185delGAG; p.Glu395del (Het),<br>Likely pathogenic	Mother (Het)<br>Father (WT)	*ZFPM2*: c.1632G>A; p.(Met544Ile) (Het)	Index & Father	0.9900	TG	LB/B	20.5
**28**	46,XY Male	External genitalia: meatal opening scrotal, gonads inguinal, labioscrotal fusion fused<br>Internal genitalia: testes<br>Severe - PGD<br>Father healthy	c.58G>C; p.Val20Leu (Het),<br>Likely pathogenic	Father (Het)	*CDH23*: c.1096G>A; p.(Ala366Thr) (Het)	Index	0.9475	TG	B/B	25.6
					NR1H2: c.515_516insCAA; p.(Arg171_Lys172insAsn) (Het)	Index	ND	ND	VUS/VUS	ND
**29**	46,XY Female	External genitalia: typical female<br>Internal genitalia: testes.<br>Mother healthy<br>Opposite sex - PGD	c.268G>C; p.Gly90Arg (Het),<br>VUS	NA	*ZFPM2*: c.302G>A; p.(Gly101Glu) (Het)	Index	0.9975	TG	B/VUS	25.2
					SRA1: c.94C>G; p.(Gln32Glu) (Het)	Index	0.9825	TG	B/B	26.7
					FBLN2: c.385G>A; p.(Asp129Asn) (Het)	Index	0.8675	MM	LB/VUS	29.1
**30**	46,XY Female	External genitalia: meatal opening typical female, gonads labioscrotal, genital tubercle <10, labioscrotal fusion: unfused<br>Internal genitalia dysplastic testes<br>Opposite sex - PGD	c.614_615insC; p.Gln206Thrfs*20 (Het),<br>Pathogenic	NA	*GLI2*: c.4332G>A; p.(Met1444Ile) (Het)	Index	0.9400	TG	B/B	15.95
					GLI2: c.4333C>T; p.(Leu1445Phe) (Het)	Index	0.9400	TG	B/B	22.4

#Pathogenicity score with *NR5A1*/SF-1 variant (ORVAL), ᵹ Digenic effect prediction with *NR5A1*/SF-1 variant (ORVAL-Digenic effect predictor), *ACMG American College of Medical Genetics classification, CGD, complete gonadal dysgenesis; DSD, differences of sex development, Het, heterozygous; Hom, homozygous; PGD, partial gonadal dysgenesis; CGD, complete gonadal dysgenesis; DSD, differences of sex development, Het, heterozygous; Hom, homozygous; WT, Wild type; PGD, partial gonadal dysgenesis; POI, primary ovarian insufficiency; NSDU, Non-specific disorder of under masculinisation, TDSD; testicular DSD; TG, True Digenic, MM; Monogenic + Modifier, NA; Not available; ND, not defined, B, Benign; LB, Likely Benign; VUS, Variant of unknown significance; *NR1H2*: c.515_516insCAA; p.(Arg171_Lys172insAsn), not defined in ORVAL but included in the analysis due to pathogenicity (VUS). *FLNB*: c.2195A>G; p.(Tyr732Cys). *DHRS7*: c.431G>A; p.(Arg144His), *KANK1*: c.1322C>T; p.(Thr441Ile), *CCDC59*: c.499A>G; p.(Thr167Ala), *SYNM*: c.361C>A; p.(Gln121Lys), *SYNM*: c.368C>T; p.(Ala123Val) below ORVAL threshold (≥ 0.85 (hg38)) but included in the analysis due to their pathogenicity (VUS). *AKR1C3*, NM_003739.6; *APC*, NM_000038.6; *AXIN1*, NM_003502.4; *CBX2*, NM_005189.3; *CCDC59*, NM_014167.5; *CDH23*, NM_022124.6; *CITED2*, NM_006079.5; *COL1A1*, NM_000088.4; *DHRS7*, NM_016029.4; *DKK1*, NM_012242.4; *DMRT2*, NM_181872.6; *DOCK8*, NM_203447.4; *ETNK2*, NM_018208.4; *FBLN2*, NM_001004019.2; *FDXR*, NM_024417.5; *FLNB*, NM_001457.4; *GATA5*, NM_080473.5; *GLI2*, NM_005270.5; *GLI3*, NM_000168.6; *HHAT*, NM_018194.6; *INO80*, NM_017553.3; *KANK1*, NM_015158.5; *KAT6B*, NM_012330.4; *LGR5*, NM_003667.4; *LRP6*, NM_002336.3; *MAPK14*, NM_139012.3; *MKKS*, NM_170784.3; *MYO7A*, NM_000260.4; *NOS1*, NM_000620.5; *NR1H2*, NM_007121.7; *NR5A1*, NM_004959.5; *PDGFRA*, NM_000358.3; *PKD1*, NM_001009944.3; *PLXNB1*, NM_001130082.3; *PPARGC1B*, NM_133263.4; *PTCH1*, NM_000264.5; *RXFP2*, NM_130806.5; SEMA6D, NM_001358351.3/ENST00000536845.7; *SFRP1*, NM_003012.5; *SPRY4*, NM_001127496.3; *SRA1*, ENST00000336283.6; *SRCAP*, NM_006662.3; *SYNM*, NM_145728.3; *TBCE*, NM_003193.5; *TBX2*, NM_005994.4; *ZFPM2*, NM_012082.4; *ZNF462*, NM_021224.6.

Table S3: Gene variant characterisation and *in silico* analysis of 22 DSD cases with predicted oligogenic variant combinations.

						***In silico* analysis**									
**Index case**	**Additional variants (Zygosity)**	**Clinical significance (ACMG) VarSome**	**Clinical significance (ACMG) Franklin**	**gnomAD AF (v3.2.1)**	**dbSNP**	**Mutation Taster**	**Polyphen**	**SIFT**	**Provean**	**Panther**	**SNPs & Go**	**M-CAP**	**CADD**	**REVEL**	**Previously reported**
**7**	*COL9A3*: c.43_48del; p.(Leu15_Leu16del) (Het)	VUS (PM1,PP5,BS2,BP4,BP6)	VUS (PM2,PM4)	5,63E-05	ND	ND	ND	ND	ND	ND	ND	ND	ND	ND	No
**8**	*DHX37*: c.904G>A; p.(Gly302Ser) (Het)*	VUS (PP3,PM1,PM2)	VUS (PM2)	3,94E-05	ND	VUS, 1	0.998,D	VUS, 0.07	P, (-5.02)	Prdam	Neu	B	24.9	B	No
**9**	*PKD1*: c.6598C>T; p.(Arg2200Cys) (Het)	B (BP6,BS1,BS2,BP4,BP1)	VUS (PM2)	9,04E-03	rs140869992	VUS, 1	0.854,P	B, 0.046	B,(-1.48)	ND	Neu	ND	22.9	B	Yes[117]
	CITED2: c.117_119del; p.(His39del) (Het)	B (BS1,BS2,BP69	LB (PM2, BS2,BP3,BP6)	1,56E-03	rs749590293	ND	ND	ND	ND	ND	ND	ND	22.0	ND	No
	PDGFRA: c.1285G>A; p.(Gly429Arg)(Het)	LB (BP4,BP1,BP6)	VUS (PM2)	4,00E-04	rs150577828	B, 0.9923	0.202,B	B, 0.115	B, (-1.75)	ND	Dis	B	22.4	B	Yes[118]
	FLNB: c.4233C>G; p.(Phe1411Leu) (Het)	B (BP6,BS1,BS2,BP1)	LB (PP3,PP2,BS2, BP6)	6,37E-04	rs143831841	VUS, 1	0.969,D	P, 0.003	P, (-5.75)	ND	ND	P	27.2	P	No
	FLNB: c.6017A>G; p.(Lys2006Arg) (Het)	B (BP6,BS1,BS2,BP4,BP1)	B (BA1,BS1,BS2, BP6)	9,73E-03	rs62621996	B, 0.9236	0.011,B	B, 0.635	B, (-0.22)	ND	Neu	ND	21.0	B	No
	TBCE: c.214C>T; p.(Pro72Ser) (Het)	B (BP6,BS1,BS2,BP4,BP1)	B (BA1,BS1,BS2, BP6)	2,82E-03	rs62620041	VUS, 1	0.978,D	VUS, 0.02	P, (-5.12)	PsDam	Neu	ND	23.2	B	No
**10**	*ZFPM2*: c.292G>A; p.(Asp98Asn) (Het)	B (BP6,BS2,BP4,PM1)	B (BA1, BS2,BP4,BP6)	3,00E-03	rs202217256	VUS, 1	0.505,P	B, 0.422	B,0	ND	Neu	B	23.9	B	Yes[119]
	CCDC59: c.499A>G; p.(Thr167Ala) (Het)	VUS (PM2,BP4)	VUS (PM2)	6,57E-06	rs143136847	B, 0.9853	0.993,D	VUS, 0.006	B, (-2.09)	Prdam	Neu	B	25.3	B	Yes[120]
**11**	*GLI3*: c.2179G>A; p.(Gly727Arg) (Het)	B (BP6,BS1,BS2,BP1,BP4,PP5)	VUS (PM2)	5,30E-03	rs121917710	VUS, 1	0.803,P	VUS, 0.013	P, (-7.34)	Prdam	Dis	ND	25.5	B	Yes[121]
	KANK1: c.1322C>T; p.(Thr441Ile) (Het)	VUS (PM2,BP1)	VUS (PM2,BP4)	ND	ND	VUS, 1	0.999,D	B, 0.091	B, (-2.19)	Prdam	Neu	B	24.3	B	No
**12**	*GLI3*: c.2179G>A; p.(Gly727Arg) (Het)	B (BS1,BS2,BP6,BP1,BP4,PP5)	VUS (PM2,PM5)	5,30E-03	rs121917710	VUS, 1	0.803,P	VUS, 0.013	P, (-7.34)	Prdam	Dis	ND	25.5	B	Yes[121]
	APC: c.7514G>A; p.(Arg2505Gln) (Het)	B (BP6,BS2,BP4,BP1)	B (BS1,BS2,BP6)	9,40E-04	rs147549623	VUS, 1	0.961,D	B, 0.227	B, 0.07	Prdam	Neu	VUS	23.8	VUS	Yes[122]
	PKD1: c.12436G>A; p.(Val4146Ile) (Het)	B (BP6,BS1,BS2,BP4,PP5,PM1)	VUS (PM2,BP4)	3,29E-03	rs148478410	B, 0.965	0.879,P	B, 0.163	B, (-0.76)	ND	ND	B	23.3	B	Yes[123]

	SYNM: c.361C>A; p.(Gln121Lys) (Het)	LB (PM2,BP1,BP4)	VUS (PM2)	ND	ND	VUS, 1	0.007,B	B, 0.104	B, (-1.37)	Prben	Neu	P	19.38	B	No
	SYNM: c.368C>T; p.(Ala123Val) (Het)	LB 8PM2,BP1,BP49	VUS (PM2)	ND	ND	VUS, 0.978	0.647,P	B, 0.071	B, (-1.96)	Prben	Neu	P	20.5	VUS	No
**13**	*GLI3*: c.2179G>A; p.(Gly727Arg) (Het)	B (BS1,BS2,BP4,BP1,BP6,PP5)	VUS (PM2)	5,08E-03	rs121917710	VUS, 1	0.803,P	VUS, 0.013	P. (-7.34)	Prdam	Dis	ND	25.5	B	Yes[121]
	CBX2: c.849G>T; p.(Lys283Asn) (Het)	B (BS1,BS2,BP4,BP1,BP6,BP3)	B (PM2,BS2,BP4)	3,56E-03	rs149210919	B, 0.999	0.527,P	VUS, 1	B, (-1.31)	Prben	Neu	B	20.7	B	No
**14**	*SPRY4*: c.55C>G; p.(Gln19Glu) (Het)	VUS (PM2,B19	VUS (PM2)	ND	ND	VUS, 1	0.979,D	VUS, 0.007	VUS, (-2.67)	Prdam	Neu	VUS	24.6	B	No
	TBCE: c.214C>T; p.(Pro72Ser) (Het)	B (BS1,BS2,BP4,BP6)	B (BA1,BS1,BS2, BP6)	2,82E-03	rs62620041	VUS, 0.9999	D,0.978	VUS, 0.02	P, (-5.12)	Psdam	Neu	ND	23.2	B	No
**15**	*INO80*: c.3842G>A; p.(Arg1281Gln) (Het)	B (BS2,BP4,PP2)	VUS (PM2,PP2)	1,30E-03	rs141223506	VUS, 1	0.885,P	VUS, 0.02	B, (-1.29)	Prdam	Neu	B	27.6	B	No
	FLNB: c.6956T>C; p.(Ile2319Thr) (Het)	B (BP6,BS1,BS2,BP4,BP1)	B (PP3,PP2,BA1, BS2,BP6)	7,40E-03	rs116826041	VUS, 0.9999	0.535,P	P, 0.002	VUS, (-3.58)	ND	Neu	ND	28.0	P	No
	SPRY4: c.653C>A; p.(Ser218Tyr) (Het)	VUS (PS3,PM1,BP4)	VUS (PM2,PP2,PP3)	4,29E-03	rs139512218	VUS, 1	0.964,D	P, 0.001	VUS, (-3.55)	Prdam	Neu	VUS	27.8	B	No
	MKKS: c.724G>T; p.(Ala242Ser) (Het)	LB (BS2,BP4,PP5,PM1)	VUS (PM2)	4,83E-03	rs74315394	VUS, 0.9999	0.868,P	VUS, 0.025	B, (-2.28)	Psdam	ND	B	24.9	VUS	Yes[69]
	FDXR: c.815C>T; p.(Pro272Leu) (Het)	VUS (PM2,BP1)	VUS (PM2,BP7)	1,32E-05	rs753592918	VUS, 1	0.817,P	VUS, 0.004	P, (-9.37)	ND	Neu	VUS	27.6	B	No
**16**	FLNB: c.6956T>C; p.(Ile2319Thr) (Het)	B (BP6,BS1,BS2,BP4,BP1)	B (PP3,PP2,BA1, BS2,BP6)	7,40E-03	rs116826041	VUS, 1	0.514,P	P, 0.002	VUS, (-3.58)	ND	Neu	ND	28.0	P	Yes[124]
	KAT6B: c.5252C>A; p.(Pro1751His) (Het)	VUS (PM2,PP3,BP1)	VUS (PM2,PP2)	ND	ND	VUS, 1	0.899,D	P, 0	P, (-6.87)	Prdam	Neu	VUS	24.4	VUS	No
	MYO7A: c.2293C>A; p.(Leu765Met) (Het)	B (BP4,PM2)	LB (PM2,BS2,BP6)	2,63E-04	rs201203036	VUS, 0.9999	0.629,D	VUS, 0.013	B, (-1.47)	ND	Dis	VUS	23.2	B	No
	PKD1: c.2081C>T; p.(Pro694Leu) (Het)	LB (BP4,PM1,PM2)	VUS (PM2,BP6)	2,27E-04	rs138575342	VUS, 0.9999	0.899,D	B, 0.101	VUS, (-3.61)	ND	Neu	ND	25.4	B	Yes[125]
**17**	*SEMA6D*: c.626G>A; p.(Arg209His) (Het)	LB (BP1, PM2)	VUS (PM2)	3,99E-03	ND	VUS, 1	1,D	P, 0	P , (-4.58)	Prdam	Dis	ND	32	VUS	No
	PDGFRA: c.1285G>A; p.(Gly429Arg)(Het)	LB (PP5,PM2,PP2,BP4)	VUS (PM2)	4,00E-04	rs150577828	B, 0.447	0.001,B	B, 0.447	VUS, (-3.65)	Prben	Neu	B	16.3	B	Yes[118]
	ZNF462: c.4093G>A; p.(Glu1365Lys)	B (BS1,BS2,BP4,BP1)	VUS (PP2,BP6)	4,40E-04	rs190490829	VUS, 1	0.945,D	VUS, 0.005	B, (-1.58)	Prben	Neu	B	25.1	B	No
**18**	*DKK1*: c.470G>T; p.Ser157Ile	B (BS1,BS2,BP4,BP6,PM1)	LB (PM2,BS2,BP4, BP6)	7,29E-04	rs143388912	VUS, 0.9999	0.347,B	B, 0.12	VUS, (-2.67)	ND	Neu	B	21.7	B	Yes[126]
	AXIN1: c.1485C>G; p.(Asp495Glu)	B (BS1,BS2,BP4,BP6,BP1)	VUS (PM2,BP4)	5,00E-03	rs146947903	B, 0.626	0.054,B	B, 0.379	B, (-0.78)	Prdam	Neu	ND	17.2	B	Yes[127]
**19**	*SFRP1*: c.539C>T; p.(Pro180Leu) (Het)	B (BS1,BS2,BP4,BP1)	B (CADD 26.8)	1,25E-04	rs745486439	VUS, 1	0.068,B	VUS, 0.006	P, (-4.54)	ND	Neu	B	26.8	B	No
	COL1A1: c.1559A>G; p.(Lys520Arg) (Het)	VUS (PM1,PM2)	VUS (CADD 23.1)	ND	ND	B, 0.9999	0.457,P	B, 0.47	B, (-0.5)	ND	Neu	B	23.1	B	No

20	*LRP6*: c.4402G>A; p.(Ala1468Thr) (Het)	B (BS1,BS2,PP3)	VUS (PM2,PP2,PP3)	1,97E-05	rs769406679	VUS, 1	0.993,D	P, 0	VUS, (-2.96)	Prdam	Neu	P	27.4	VUS	No
	ETNK2: c.920A>C; p.(Gln307Pro) (Het)	VUS (PM2,BP4)	VUS (PM2)	1,20E-02	rs767749304	VUS, 1	0.357,B	B, 0.037	VUS, (-3.56)	ND	ND	B	22.9	B	No
21	*GLI2*: c.803C>T; p.(Ala268Val) (Het)	B (BS1,BS2,BP4,BP6,BP1)	B (BA1,BS2,BP6)	2,33E-03	rs146992756	VUS, 1	0.971,D	VUS, 0.034	VUS, (-3.49)	Prdam	ND	ND	25.0	B	Yes[128]
	CDH23: c.5831T>C; p.(Leu1944Ser) (Het)	B (BP4,PM1,PM2)	VUS (PM2,BP6)	2,43E-04	rs201876362	B, 0.9973	0.379,B	ND	ND	ND	Neu	B	22.6	B	No
	LGR5: c.1148A>G; p.(His383Arg) (Het)	B (BS1,BS2,BP4,BP6,BP1)	LB (PM2,BS2,BP4)	2,41E-03	rs12303775	B, 0.9924	0.041,B	B, 0.188	P, (-6.09)	Prben	Neu	B	22.3	B	No
	GATA5: c.232G>A; p.Gly78Ser (Het)	B (BS1,BS2,BP4)	LB (PM2,BS2,BP6)	2,14E-03	rs572247741	B, 0.8815	0.276,B	B, 0.446	B, (- 0.56)	Prdam	Neu	ND	15.44	B	No
	PPARGC1B: c.1088C>T; p.(Thr363Met) (Het)	B (BS1,BS2,BP4,BP6,BP1)	B (BS1,BS2,BP4, BP6)	4,57E-03	rs45526537	B, 0.9953	0.068,B	B, 0.133	B, (-1.44)	Prben	Neu	ND	17.2	B	No
	PPARGC1B: c.1499C>T; p.(Ser500Leu) (Het)	B (BS1,BS2,BP4,BP6,BP1,BP39	B (BS1,BS2,BP4, BP6)	6,50E-03	rs45549037	VUS, 1	0.031,B	VUS, 0.017	B, (-1.64)	Prben	Neu	ND	10.03	B	No
22	*MAPK14*: c.1028A>G; p.(Asp343Gly) (Het)	B (BS1,BS2,BP4,BP6,PP2)	LB (PM2,BS2,BP6)	7,56E-04	ND	VUS, 0.999	0.002,B	B, 0.132	VUS, (-3.16)	ND	Neu	B	23.4	B	Yes[129]
	PLXNB1: c.1360A>C; p.(Ser454Arg) (Het)	B (BS1,BS2,BP4,PS1,PP2)	VUS (PM2)	1,09E-02	rs114964512	VUS, 1	0.677,P	B, 0.097	VUS, (-2.42)	Psdam	Neu	ND	25.7	B	Yes[130]
	PTCH1: c.4324C>T; p.(Leu277Met) (Het)	B (BS2,BP4,BP1,BP6)	LB (PM2,BP7,BP6)	6,11E-04	rs143464326	B, 0.9862	0.0,B	B, 0.14	B, (-0.53)	ND	Neu	B	20.2	B	No
	HHAT: c.829C>A; p.(Leu277Met) (Het)	VUS (PM2, BP1)	VUS (PM2)	ND	ND	B, 9959	0.995, D	VUS, 0.011	B, (- 0.7)	Prdam	Neu	B	23.8	VUS	No
	HHAT: c.1130A>G; p.(Tyr377Cys) (Het)	VUS (PM2, BP1)	VUS (PM2)	ND	rs745491597	B, 06551	0.065,B	B, 0.051	VUS (-2.84)	Psdam	Dis	B	23.0	B	No
23	*SRA1*: c.413G>A ; p.(Gly138Glu) (Het)	B (BS1,BS2,BP4,BP6,BP1)	LB (PM2,BS2,BP4, BP6)	ND	rs149499620	VUS, 0.9999	0.016,B	B, 0.366	B, (-0.44)	Psdam	ND	B	19.2	B	No
	MYO7A: c.1868G>A; p.(Arg623His) (Het)	B (BS1,BS2,BP4,PM1)	B (BS1,BS2,BP6)	2,96E-04	rs111033416	VUS, 0.9999	0.973,D	VUS, 0.004	VUS, (-3-6)	ND	Dis	VUS	26.8	VUS	Yes[131]
	SRCAP: c.4499C>T; p.(Pro1500Leu) (Het)	B (BS2,BP4,BP1,BP3)	VUS (PM2)	1,31E-05	rs770748860	VUS, 0.10000	0.009,B	P, 0.001	B, (-1.92)	Prben	Neu	VUS	22.1	B	No
	SRCAP:c.4603C>G ; p.(Pro1535Ala) (Het)	B (BP6,BS1,BS2,BP4,BP1)	B (PP2,BA1,BS1, BS2)	6,61E-03	rs117804715	B, 0.9924	0.017,B	VUS, 0.004	B, (-1.86)	Prben	Neu	ND	17.8	B	No
24	*TBX2*: c.1139C>G; p.(Pro380Arg) (Het)	VUS (PM2,BP4)	VUS (PM2)	1,31E-05	rs765795326	B, 0991	0.891,P	P, 0.001	B , (-1.78)	Prdam	ND	P	24.7	B	No
	FLNB: c.2195A>G; p.(Tyr732Cys) (Het)	VUS (PP3,PM2)	VUS (PM2,PP3,PP2)	6,57E-06	ND	B, 0.998	0.873,P	P, 0	P, (-5.78)	ND	Dis	VUS	23.8	VUS	Yes[132]
25	*NOS1*: c.335C>T; p.(Thr112Ile) (Het)	B (BS1,BS2,BP4,PP2)	B (CADD 23.2)	1,38E-04	rs75386866	B, 0.8285	0.209,B	VUS, 0.02	VUS, (-2.62)	Prdam	Neu	B	23.2	B	No
	DMRT2: c.674C>G; p.(Pro225Arg) (Het)	LB (BP1,BP4,PM2)	LB (CADD 24.7)	6,57E-06	rs752382017	VUS, 1	0.467,P	B, 0.034	B, (-1.21)	Psdam	ND	B	24.7	B	No
	FLNB: c.6017A>G; p.(Lys2006Arg) (Het)	B (BP6,BS1,BS2,BP4)	B (CADD 21.0)	9,73E-03	rs62621996	B, 0.9236	0.011,B	B, 0.657	B, (-0.22)	ND	Neu	ND	21.0	B	No

	AKR1C3: c.548A>T; p.Lys183Met (Het)	B (BS1,BS2,BP4)	B (CADD 25.4)	2,91E-03	rs61730879	B, 0.9966	0.825,P	P, 0	P, (-5.62)	Prdam	Neu	ND	25.6	B	No
	DHRS7: c.431G>A; p.(Arg144His) (Het)	VUS (PM2, BP4)	VUS (CADD 26.7)	6,57E-06	rs539643284	VUS, 1	0.991,D	VUS, 0.007	VUS, (-3.9)	Prdam	Neu	B	26.7	VUS	No
**26**	*DOCK8*: c.137G>A; p.(Gly46Asp) (Het)	LB (BP4,BP1,PM2)	VUS (PM2,BP4)	6,58E-06	rs758437810	B, 1	ND	ND	ND	ND	Neu	B	22.1	ND	No
	KAT6B: c.2134G>T; p.(Gly712Trp) (Het)	B (BS2,BP1)	VUS (PM2,BS2)	1,32E-05	rs530449640	B, 0.9471	ND	VUS, 0.005	VUS, (-3.64)	Psdam	Neu	VUS	28.0	B	No
	RXFP2: c.1594C>G; p.(Arg532Gly) (Het)	B (BP4, BP1,PM2)	VUS (PM2,BP4)	9,67E-04	rs138951290	VUS, 0.9999	ND	VUS, 0.045	P, (-5.13)	B	Dis	B	20.4	B	No
**27**	ZFPM2: c.1632G>A; p.(Met544Ile) (Het)*	LB (PS1, PS3, PP5, PM1, BP4, BS2,BS3)	B (PP3,BA1,BS2, BP6)	3,45E-03	rs187043152	VUS, 1	0.021,B	B, 0.083	B, (-0.9)	ND	Neu	VUS	20.5	VUS	Yes[111]
**28**	*CDH23*: c.1096G>A; p.(Ala366Thr) (Het)	B (BS1,BS2,BP4, BP1,BP6)	B (BA1,BS2,BP6)	6,53E-03	rs143282422	VUS, 1	0.9973,B	B, 0.379	B (-0.98)	ND	Neu	ND	25.6	B	Yes[133]
	NR1H2: c.515_516insCAA; p.(Arg171_Lys172insAsn) (Het)	VUS (PM2,PM4)	VUS (PM2,PM4)	ND	ND	ND	ND	ND	ND	ND	ND	ND	ND	ND	No
**29**	*ZFPM2*: c.302G>A; p.(Gly101Glu) (Het)*	B (BS2,BP4,PM1)	VUS (PM2,PP3)	7,89E-05	rs199605561	VUS, 1	P, 0	B, 0.362	B, 1	ND	Neu	B	25.2	B	Yes[32]
	SRA1: c.94C>G; p.(Gln32Glu) (Het)*	B (BS1,BS2,BP4,BP1,P P5)	B (BS1,BS2,BP6)	5,91E-03	rs35610885	VUS, 1	B,0.07	B,1	B, (-1.43)	Psdam	ND	B	26.7	B	Yes[32]
	FBLN2: c.385G>A; p.(Asp129Asn) (Het)*	LB (BP4,BP1,PM2)	VUS (PM2)	8,54E-05	rs533268036	VUS, 0.9999	D	P,0	P, (-2.38)	Psdam	Neu	7	29.1	VUS	Yes[32]
**30**	*GLI2*: c.4332G>A; p.(Met1444Ile) (Het)*	B (BA1,BP6,BP4,BP4,B P1,BP3)	B (BA1,BS2,BP4, BP6)	4,72E-03	rs146467786	VUS, 1	0.048,B	B,0,048	B, (-1.39)	Psdam	Dis	ND	15.95	B	Yes[134]
	GLI2: c.4333C>T; p.(Leu1445Phe) (Het)	B (BA1,BP6,BP4,BP4,B P1)	B (BA1,BS2,BP4, BP6)	4,72E-03	rs146207623	VUS, 1	VUS	VUS,0.009	B, (-2.19)	Prben	Dis	ND	22.4	B	Yes[135]

*Variants previously reported in the SF1next study cohort, Het, heterozygous; ND, not defined, B, Benign; LB, Likely Benign; VUS, Variant of unknown significance; P, Pathogenic; gnomAD AF, genome aggregation database allele frequency. D, Deleterious; Dis, Disease causing; Neu, Neutral; Prben, probably benign; Prdam, probably damaging, Psdam, possibly damaging. *In silico* analysis with webtools: SIFT (https://sift.bii.a-star.edu.sg/), Provean (https://www.jcvi.org/), PolyPhen-2 (http://genetics.bwh.harvard.edu/pph2/), MutationTaster (https://www.genecascade.org/MutationTaster2021/#transcript), Panther (http://pantherdb.org/), SNPs&Go (https://snps-and-go.biocomp.unibo.it/snps-and-go/), M-CAP (http://bejerano.stanford.edu/mcap/), CADD (https://cadd.gs.washington.edu/), REVEL (https://sites.google.com/site/revelgenomics/). *AKR1C3*, NM_003739.6; *APC*, NM_000038.6; *AXIN1*, NM_003502.4; *CBX2*, NM_005189.3; *CCDC59*, NM_014167.5; *CDH23*, NM_022124.6; *CITED2*, NM_006079.5; *COL1A1*, NM_000088.4; *DHRS7*, NM_016029.4; *DKK1*, NM_012242.4; *DMRT2*, NM_181872.6; *DOCK8*, NM_203447.4; *ETNK2*, NM_018208.4; *FBLN2*, NM_001004019.2; *FDXR*, NM_024417.5; *FLNB*, NM_001457.4; *GATA5*, NM_080473.5; *GLI2*, NM_005270.5; *GLI3*, NM_000168.6; *HHAT*, NM_018194.6; *INO80*, NM_017553.3; *KANK1*, NM_015158.5; *KAT6B*, NM_012330.4; *LGR5*, NM_003667.4; *LRP6*, NM_002336.3; *MAPK14*, NM_139012.3; *MKKS*, NM_170784.3; *MYO7A*, NM_000260.4; *NOS1*, NM_000620.5; *NR1H2*, NM_007121.7; *PDGFRA*, NM_000358.3; *PKD1*, NM_001009944.3; *PLXNB1*, NM_001130082.3; *PPARGC1B*, NM_133263.4; *PTCH1*, NM_000264.5; *RXFP2*, NM_130806.5; SEMA6D, NM_001358351.3/ENST00000536845.7; *SFRP1*, NM_003012.5; *SPRY4*, NM_001127496.3; *SRA1*, ENST00000336283.6; *SRCAP*, NM_006662.3; *SYNM*, NM_145728.3; *TBCE*, NM_003193.5; *TBX2*, NM_005994.4; *ZFPM2*, NM_012082.4; *ZNF462*, NM_021224.6.

Figure S2: Density plot representing the PHRED-scaled CADD scores of 64 variants identified in 22 index cases (plus their family members) with predicted combinations involving *NR5A1*/SF-1 variants. The density plot visualises how the CADD scores of the 58 variants are distributed. PHRED-scaled CADD score ≥ 15 are predicted to be in the top 0.5% of most deleterious substitutions that can occur in the human genome. One candidate variant (*NR1H2*, p.Arg171_Lys172insAsn) was not predicted in CADD.

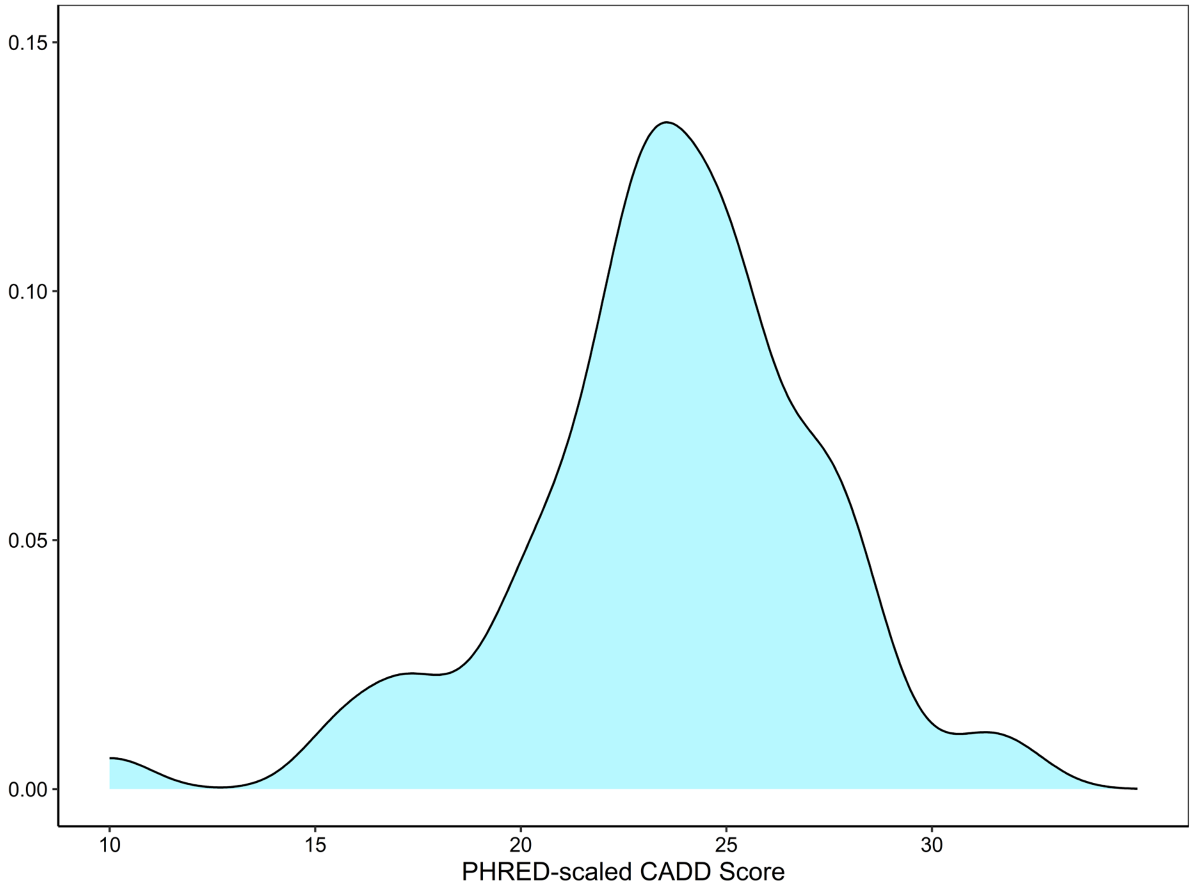

Table S4: List of additional variants per case, discarded after filtering due to lack of association with the observed phenotype of the individuals.

Index case	Gene	Transcript	dbSNP	c. (HGVS)	p. (HGVS)	Found in	Zygosity	Clinical significance (ACMG) VarSome	Pathogenicity score with *NR5A1*/SF-1 variant (ORVAL)	Previously reported
9	*CDH3*	NM_001793.6	rs34394404	c.1285G>A	p.Val429Ile	Index & Father	Het	B	0.9000	No
	SMAD3	NM_005902.4	rs1264997614	c.575G>C	p.Ser192Thr	Index & Father	Het	VUS	0.9800	No
	RORC	NM_005060.4	rs17582155	c.28C>T	p.Arg10*	Index & Father	Hom	LP	0.9825	No
	PRKD1	NM_002742.3	rs572057574	c.20T>C	p.Leu7Pro	Index	Het	B	0.9475	No
	ESRRA	NM_004451.5	rs117285599	c.92C>T	p.Thr31Ile	Index & Father	Het	B	0.9100	No
	CFTR	NM_000492.4	rs143456784	c.1064C>T	p.Pro355Leu	Index & Father	Het	VUS	0.9500	Yes[136]
	AMIGO2	NM_001370299.1	rs769234837	c.575C>T	p.Ala192Val	Index & Father	Het	LB	0.8750	No
	CEP250	NM_007186.6	ND	c.1210G>T	p.Asp404Tyr	Index & Father	Het	VUS	0.8550	No
	ATM	NM_000051.4	rs1800054	c.146C>G	p.Ser49Cys	Index	Het	B	0.9800	Yes[137]
	CCIN	NM_005893.3	rs148954505	c.1183C>T	p.Arg395Cys	Index & Father	Het	VUS	0.7800	No
10	*MED13*	NM_005121.3	rs140673828	c.4642A>G	p.Ser1548Gly	Index	Het	B	0.9725	No
	GRIP1	NM_001366722.1	rs145115262	c.2606G>A	p.Arg869Gln	Index	Het	B	0.9700	No
	NR2C1	NM_003297.4	rs144726031	c.151G>A	p.Gly51Ser	Index	Het	B	0.9625	No
	ROS1	NM_002944.3	rs142201886	c.649C>A	p.Pro217Thr	Index	Het	LB	0.9075	No
	MYO1G	NM_033054.3	ND	c.1216A>T	p.Ser406Cys	Index	Het	LP	0.8950	No
	TBX10	NM_005995.5	rs141787375	c.259G>A	p.Val87Ile	Index	Het	LB	0.8800	No
	FANCM	NM_020937.4	rs768006618	c.2586_2589del	p.Lys863Ilefs*12	Index	Het	P	ND	Yes[138]
	ACAT1	NM_000019.4	rs147872303	c.1217A>G	p.Glu406Gly	Index	Het	VUS	0.8950	Yes[139]
	MNX1	NM_005515.4	rs755038175	c.516_518del	p.Ala174del	Index	Het	VUS	ND	No
	KRAS	NM_004985.5	rs397517043	c.531_533dup	p.Lys180dup	Index	Het	VUS	ND	No
11	*HOXA11*	NM_005523.6	rs143812636	c.396G>C	p.Arg132Ser	Index & Father	Het	B	0.9750	No
	FBN2	NM_001999.4	rs56168072	c.4312G>A	p.Glu1438Lys	Index & Father	Het	B	0.9250	Yes[140]
	RIPK2	NM_003821.6	ND	c.604C>G	p.Gln202Glu	Index & Father	Het	B	0.9250	No
	CACNA1A	NM_001127222.2	rs749638821	c.6656_6658del	p.His2219del	Index only	Het	VUS	0.9250	No
	NSMF	NM_001130969.3	ND	c.2T>A	p.Met1Lys	Index only	Het	LP	0.8675	No
	arr[GRCh37]16 p12.3p12.3(18894303-19162153)x3	ND	ND	ND	ND	Index	ND	VUS	ND	No
12	*NME7*	NM_197972.2	rs755143347	c.998_1001dup	p.Phe334Leufs*8	Siblings & Father	Het	VUS	0.8450	No
13	*SFRP2*	NM_003013.3	rs559360607	c.30_32dup	p.Leu11dup	Index & Father	Het	B	0.9400	No
	MED22	NM_133640.5	rs75461307	c.475G>A	p.Asp159Asn	Index & Father	Het	B	0.8725	No
	CYP11B2	NM_000498.3	rs121912977	c.763G>T	p.Glu255*	Index	Het	LP	0.8400	Yes[141]
	DHODH	NM_001361.5	rs764930937	c.850A>G	p.Thr284Ala	Index & Father	Het	P	0.8800	Yes[142]
	TRERF1	NM_001361.5	rs61756353	c.356G>A	p.Gly119Asp	Index & Father	Het	B	0.9925	No
14	ACTN4	NM_004924.6	rs368725797	c.1216C>T	p.Arg406Cys	Index	Het	LB	0.9700	No
	MAP3K1	NM_005921.2	rs55694258	c.710A>G	p.Gln237Arg	Index	Het	B	0.5350	No
15	*PEG3*	NM_006210.3	rs191932140	c.1669T>C	p.Tyr557His	Index & Mother	Het	LB	0.9275	No

	ATG7	NM_001127496.3	ND	c.409G>T	p.Ala137Ser	Index & Mother	Het	VUS	0.9300	No
	MRPS22	NM_020191.4	ND	c.698C>A	p.Ala233Asp	Index	Het	VUS	ND	No
	MED12	NM_005120.3	ND	c.6327_6329del	p.Gln2115del	Index	Hemi	VUS	0.7625	No
	AMH	NM_000479.5	rs139265145	c.428C>T	p.Thr143Ile	Index	Het	B	0.7775	Yes[143]
**16**	*GJA1*	NM_000165.5	rs17653265	c.758C>T	p.Ala253Val	Index	Het	B	0.8925	Yes[144]
	VDR	NM_000376.3	ND	c.220C>T	p.Arg74Cys	Index	Het	LP	0.9850	No
**17**	*CYR61*	NM_001554.5	rs9658587	c.1000C>T	p.Arg334Trp	Index & Twin & Mother	Het	B	ND	No
	TGFBI	NM_000358.3	rs121909217	c.1998G>C	p.Arg666Ser	Index & Twin & Mother	Het	VUS	0.600	Yes[145]
**18**	*CACNA1A*	NM_001127222.2	ND	c.6674A>C	p.Lys2225Thr	Index only	Het	VUS	0.7925	No
	TNC	NM_002160.4	ND	c.652C>A	p.Gln218Lys	Index only	Het	LB	0.9600	No
**20**	*NR4A2*	NM_006186.4	ND	c.275T>C	p.Val92Ala	Index & Father	Het	VUS	0.9850	No
	BCL9L	NM_001378213.1	rs138908070	c.1048G>A	p.Gly350Ser	Index & Father	Het	B	0.9350	No
**21**	*TBX15*	NM_001330677.2	rs141002143	c.980G>A	p.Arg327His	Index & Father	Het	B	0.9800	No
	BCLAF1	NM_014739.3	rs149487316	c.495A>T	p.Gln165His	Index & Father	Het	LB	0.9875	No
	BTRC	NM_033637.4	rs141008374	c.1240A>G	p.Thr414Ala	Index & Mother	Het	B	0.9725	No
	NOS3	NM_000603.5	rs3918201	c.2654G>T	p.Arg885Met	Index & Father	Het	B	0.9550	Yes[146]
	ZHX2	NM_014943.5	rs116577713	c.1024C>A	p.Pro342Thr	Index & Father	Het	B	0.8750	No
	PTP4A3	NM_032611.3	ND	c.293_307dup	p.Val102_His103insArgCysValAlaVal	Index & Mother	Het	VUS	ND	No
	LATS1	NM_004690.4	rs147560862	c.416G>T	p.Ser139Ile	Index & Mother	Het	B	0.9325	No
	BRCA2	NM_000059.4	rs2227943	c.2786T>C	p.Leu929Ser	Index & Mother	Het	B	0.9250	No
	BRCA2	NM_000059.4	rs2227944	c.2960A>T	p.Asn987Ile	Index & Mother	Het	B	0.9250	No
	MCM9	NM_017696.3	rs34213490	c.397A>G	p.Thr133Ala	Index & Mother	Het	B	0.8925	Yes[147]
	WNT9B	NM_003396.3	rs75199851	c.281G>A	p.Arg94Gln	Index & Father	Het	B	0.8900	No
	DACH1	NM_080759.6	rs538753327	c.203_204insGG	p.Thr69Alafs*126	Index & Father	Het	LP	ND	No
	KCND2	NM_012281.3	rs367713278	c.1598G>A	p.Arg533Gln	Index & Mother	Het	B	0.8575	No
	ATP7A	NM_000052.7	ND	c.2189A>T	p.Tyr730Phe	Index & Mother	Hemi/Het (Mother)	VUS	0.8400	No
**22**	*CACNA1B*	NM_000718.4	rs760115118	c.6830C>T	p.Thr2277Ile	Index	Het	B	0.8625	No
	LZTS1	NM_021020.5	rs149140637	c.338T>C	p.Leu113Pro	Index	Het	B	0.8800	Yes[148]
	MAPK3	NM_002746.3	ND	c.270G>C	p.Gln90His	Index	Het	LB	0.9700	No
	ESRRB	NM_004452.4	rs201344770	c.889G>A	p.Asp297Asn	Index	Het	LB	0.9550	No
	ESRRB	NM_004452.4	rs146351534	c.1237G>A	p.Val413Ile	Index	Het	B	0.9550	Yes[149]
	CDH4	NM_001794.5	ND	c.2137T>C	p.Cys713Arg	Index	Het	VUS	0.9325	No
	INSR	NM_000208.4	rs1799816	c.3034G>A	p.Val1012Met	Index	Het	B	0.9225	Yes[150]
**23**	*PRDM2*	NM_001393986.1	ND	c.80C>T	p.Pro27Leu	Index	Het	VUS	0.9750	No
	NIBAN1	NM_052966.4	ND	c.2773C>T	p.Pro925Ser	Index	Het	LB	0.8625	No
	IRS1	NM_005544.3	ND	c.64C>T	p.Pro22Ser	Index	Het	VUS	0.9700	No
	ROS1	NM_002944.3	rs56274823	c.1108T>C	p.Ser370Pro	Index	Het	B	0.9050	Yes[151]
	PCSK6	NM_002570.5	rs34631529	c.2891C>T	p.Thr964Met	Index	Het	B	0.8950	Yes[152]
	ATM	NM_000051.4	rs140856217	c.4709T>C	p.Val1570Ala	Index	Het	LB	0.8850	Yes[153]
	RANBP2	NM_006267.5	rs2433786	c.7172C>G	p.Thr2391Ser	Index	Het	B	0.8800	No
	TNC	NM_002160.4	rs144032672	c.628G>A	p.Gly210Ser	Index	Het	B	0.9750	Yes[154]
	TBX3	NM_005996.4	rs369538205	c.2087C>T	p.Ala696Val	Index	Het	B	0.9750	No
	TSC2	NM_000548.5	rs796872464	c.4621G>A	p.Asp1541Asn	Index	Het	VUS	0.9725	No

	AXIN2	NM_004655.4	rs142476324	c.1985T>C	p.Leu662Pro	Index	Het	B	0.9525	No
	SRD5A2	NM_000348.4	rs28383082	c.*43G>A	ND	Index	Het	B	0.6300	No
**24**	*ATF3*	NM_001674.4	ND	c.355G>A	p.Glu119Lys	Index	Het	LB	0.9625	No
	SETX	NM_015046.7	rs145438764	c.472T>G	p.Leu158Val	Index	Het	B	0.9025	Yes[155]
	VDR	NM_001374662.1	rs1016178956	c.35A>G	p.Asp12Gly	Index	Het	VUS	0.9475	No
	ZP4	NM_021186.5	rs749980785	c.1140G>T	p.Gln380His	Index	Het	VUS	0.4750	No
**25**	*HSPA5*	NM_005347.5	rs56136100	c.1670A>G	p.Glu557Gly	Index	Het	B	0.9725	No
	HOXA9	NM_152739.4	rs200450890	c.304G>C	p.Ala102Pro	Index	Het	B	0.9625	Yes[156]
	TRPM4	NM_017636.4	rs144421653	c.3224T>C	p.Leu1075Pro	Index	Het	LB	0.9325	Yes[157]
	TJP1	NM_001330239.4	rs373713203	c.3286G>A	p.Asp1096Asn	Index	Het	B	0.9275	No
	IL6ST	NM_002184.4	rs140433866	c.1301C>G	p.Pro434Arg	Index	Het	B	0.9100	No
	ITGA11	NM_001004439.2	rs200576562	c.3443G>T	p.Ser1148Ile	Index	Het	B	0.8975	No
	ENPP2	NM_001040092.3	ND	c.1459G>C	p.Val487Leu	Index	Het	VUS	0.7850	No
	BBS10	NM_024685.4	rs142863601	c.424G>C	p.Asp142His	Index	Het	VUS	ND	Yes[158]
	TRIM47	NM_033452.3	rs930521522	c.1310C>T	p.Ala437Val	Index	Het	VUS	0.8475	No
	CYP21A2	NM_000500.9	rs6471	c.844G>A	p.Val282Met	Index	Het	VUS	ND	Yes[159]
	CAMK1	NM_003656.5	rs138483154	c.254A>G	p.Tyr85Cys	Index	Het	VUS	0.9350	No
	SIK2	NM_015191.3	rs35789057	c.1373C>T	p.Thr458Ile	Index	Het	B	0.9225	No
	POLM	NM_013284.4	rs28382644	c.659G>C	p.Gly220Ala	Index	Het	B	0.9200	Yes[160]
	PNRC1	NM_006813.3	rs141030892	c.124T>A	p.Leu42Ile	Index	Het	LB	0.9125	No
	MACF1	NM_001394062.1	rs148290248	c.16925G>A	p.Gly5642Asp	Index	Het	B	0.8725	No
	MTSS1	NM_014751.6	rs562988130	c.1937A>G	p.Glu646Gly	Index	Het	B	0.8625	No
	SSUH2	NM_001256748.3	rs373708901	c.664G>A	p.Gly222Ser	Index	Het	VUS	0.6350	No
**26**	*NR1H4*	NM_001206977.2	rs61755050	c.518T>C	p.Met173Thr	Index	Het	LB	0.9950	Yes[161]
	CD36	NM_001371077.1	rs551607784	c.1150del	p.Ala384Glnfs*19	Index	Het	LP	0.9450	Yes[162]
	CYP3A4	NM_017460.6	rs72552799	c.389G>A	p.Arg130Gln	Index	Het	B	ND	Yes[163]
	STAR	NM_000349.3	rs34908868	c.361C>T	p.Arg121Trp	Index & Father	Het	B	0.9975	VUS
**27**	*TBX10*	NM_005995.4	rs554161959	c.724G>A	p.Ala242Thr	Index & Father	Het	VUS	0.8200	No
	EP300	NM_001429.4	rs61756764	c.2091T>G	p.Ser697Arg	Index & Father	Het	B	0.9775	Yes[164]
	ATR	NM_001184.4	ND	c.684dup	p.Leu229Thrfs*13	Index & Father	Het	LP	0.8950	No
	BCL9L	NM_182557.4	ND	c.3520A>C	p.Met1174Leu	Index	Het	B	0.9550	No
	TNC	NM_002160.4	rs113301777	c.3739C>A	p.Leu1247Ile	Index & Father	Het	B	0.8650	No
	CDH4	NM_001794.5	rs142900721	c.1567G>A	p.Gly523Ser	Index & Father	Het	B	0.8525	No
	GCK	NM_000162.5	rs771677681	c.343A>G	p.Met115Val	Index & Father	Het	LP	0.8100	Yes[165]
**28**	*NIBAN1*	NM_052966.4	rs148732242	c.929G>A	p.Arg310His	Index	Het	VUS	0.8500	No
	INHA	NM_002191.4	rs139608664	c.675T>G	p.Ser225Arg	Index	Het	LB	0.5825	No
	SRCAP	NM_006662.3	rs146290947	c.7331G>A	p.Arg2444Gln	Index	Het	B	0.9300	Yes[166]
	SCUBE2	NM_001367977.2	ND	c.692C>T	p.Thr231Ile	Index	Het	VUS	0.8450	No
	TCF7L2	NM_001367943.1	rs77673441	c.1535C>G	p.Pro512Arg	Index	Het	B	0.9825	No
**29**	*BTRC*	NM_033637.4	rs202110950	c.1793G>A	p.Arg598Gln	Index	Het	B	0.9825	No
	IRS1	NM_005544.3	rs140117962	c.3056T>C	p.Ile1019Thr	Index	Het	B	0.9100	No
	ZEB2	NM_014795.4	rs750936389	c.2342A>G	p.Asn781Ser	Index	Het	B	0.9550	No
	NCOR2	NM_006312.6	rs750908364	c.5755G>A	p.Gly1919Ser	Index	Het	B	0.9850	ND
	MACF1	NM_012090.5	rs148207245	c.2444G>T	p.Cys815Phe	Index	Het	B	0.8675	ND
	CACNG4	NM_014405.4	rs767219625	c.715C>T	p.Arg239Trp	Index	Het	VUS	0.8400	Yes[32]
	SMAD6	NM_005585.5	ND	c.1450_1451insC	p.Cys484Serfs*81	Index	Het	LP	ND	ND

30	*NAV1*	NM_020443.4	rs145865304	c.2947C>A	p.Pro983Thr	Index	Het	B	0.5325	No
	SALL4	NM_020436.5	ND	c.1287T>G	p.Phe429Leu	Index	Het	B	0.9850	No
	CDH3	NM_001793.6	rs34394404	c.1285G>A	p.Val429Ile	Index	Het	B	0.8800	No
	FGFR2	NM_000141.5	rs3750819	c.17G>C	p.Arg6Pro	Index	Het	B	0.9525	No
	BLM	NM_000057.4	rs35886055	c.419A>G	p.Glu140Gly	Index	Het	B	0.9375	No
	FOXO3	NM_001455.4	rs145259784	c.1021G>A	p.Ala341Thr	Index	Het	B	0.8575	Yes[167]
	BCL9L	NM_001378213.1	rs144029087	c.2165T>C	p.Met722Thr	Index	Het	B	0.9900	No
	GDNF	NM_000514.4	rs36119840	c.277C>T	p.Arg93Trp	Index	Het	B	0.9800	Yes[168]
	TSC2	NM_000548.5	rs45484298	c.1318G>A	p.Gly440Ser	Index	Het	B	0.9675	No
	PPARA	NM_005036.6	rs1800234	c.680T>C	p.Val227Ala	Index	Het	B	0.8650	Yes[169]
	MCM9	NM_017696.3	ND	c.1521A>T	p.Glu507Asp	Index	Het	B	0.8625	Yes[170]
	LMNA	NM_170707.4	rs150924946	c.895A>G	p.Ile299Val	Index	Het	VUS	0.8350	No
	SOX30	NM_178424.2	rs13181859	c.455C>T	p.Pro152Leu	Index	Het	B	0.6025	No
	DENND1A	NM_020946.1	rs189947178	c.2351C>A	p.Ala784Asp	Index	Het	B	0.7125	Yes[171]
	CDH7	NM_017780.4	rs192129249	c.7579A>C	p.Met2527Leu	Index	Het	B	0.7400	Yes[172]

*ACMG classification, Het, heterozygous; ND, not defined, B, Benign; LB, Likely Benign; VUS, Variant of unknown significance; P, Pathogenic; gnomAD AF, genome aggregation database allele frequency.

References to supplementary material

1. Söderhäll C, Körberg IB, Thai HT, Cao J, Chen Y, Zhang X, et al. Fine mapping analysis confirms and strengthens linkage of four chromosomal regions in familial hypospadias. Eur J Hum Genet. 2015;23(4):516-22.
2. Ashley RA, Yu Z, Fung KM, Frimberger D, Kropp BP, Penning TM, et al. Developmental evaluation of aldo-keto reductase 1C3 expression in the cryptorchid testis. Urology. 2010;76(1):67-72.
3. Hossain A, Saunders GF. Synergistic cooperation between the beta-catenin signaling pathway and steroidogenic factor 1 in the activation of the Mullerian inhibiting substance type II receptor. J Biol Chem. 2003;278(29):26511-6.
4. Loke J, Pearlman A, Radi O, Zuffardi O, Giussani U, Pallotta R, et al. Mutations in MAP3K1 tilt the balance from SOX9/FGF9 to WNT/β-catenin signaling. Hum Mol Genet. 2014;23(4):1073-83.
5. Ostrer H. Pathogenic Variants in MAP3K1 Cause 46,XY Gonadal Dysgenesis: A Review. Sex Dev. 2022;16(2-3):92-7.
6. Zhou B, Tang T, Chen P, Pu Y, Ma M, Zhang D, et al. The variations in the AXIN1 gene and susceptibility to cryptorchidism. J Pediatr Urol. 2015;11(3):132.e1-5.
7. Doghman M, Figueiredo BC, Volante M, Papotti M, Lalli E. Integrative analysis of SF-1 transcription factor dosage impact on genome-wide binding and gene expression regulation. Nucleic Acids Res. 2013;41(19):8896-907.
8. Hart D, Rodriguez Gutierrez D, Lauber-Biason A. CBX2 in DSD: The Quirky Kid on the Block. Sexual Development. 2022;16:1-9.
9. Biason-Lauber A, Konrad D, Meyer M, DeBeaufort C, Schoenle EJ. Ovaries and female phenotype in a girl with 46,XY karyotype and mutations in the CBX2 gene. Am J Hum Genet. 2009;84(5):658-63.
10. Flück CE, Audí L, Fernández-Cancio M, Sauter K-S, Martinez de LaPiscina I, Castaño L, et al. Broad Phenotypes of Disorders/Differences of Sex Development in MAMLD1 Patients Through Oligogenic Disease. Frontiers in Genetics. 2019;10(746).
11. Li L, Gao F, Fan L, Su C, Liang X, Gong C. Disorders of Sex Development in Individuals Harbouring MAMLD1 Variants: WES and Interactome Evidence of Oligogenic Inheritance. Frontiers in endocrinology. 2020;11:582516-.
12. Li L, Su C, Fan L, Gao F, Liang X, Gong C. Clinical and molecular spectrum of 46,XY disorders of sex development that harbour MAMLD1 variations: case series and review of literature. Orphanet J Rare Dis. 2020;15(1):188.
13. Piprek RP, Kloc M, Mizia P, Kubiak JZ. The Central Role of Cadherins in Gonad Development, Reproduction, and Fertility. Int J Mol Sci. 2020;21(21).
14. Calonga-Solís V, Fabbri-Scallet H, Ott F, Al-Sharkawi M, Künstner A, Wünsch L, et al. MYRF: A New Regulator of Cardiac and Early Gonadal Development-Insights from Single Cell RNA Sequencing Analysis. J Clin Med. 2022;11(16).
15. Buaas FW, Val P, Swain A. The transcription co-factor CITED2 functions during sex determination and early gonad development. Hum Mol Genet. 2009;18(16):2989-3001.
16. Larney C, Bailey TL, Koopman P. Switching on sex: transcriptional regulation of the testis-determining gene Sry. Development. 2014;141(11):2195-205.
17. Combes AN, Spiller CM, Harley VR, Sinclair AH, Dunwoodie SL, Wilhelm D, et al. Gonadal defects in Cited2-mutant mice indicate a role for SF1 in both testis and ovary differentiation. Int J Dev Biol. 2010;54(4):683-9.
18. Yuri P, Gunadi, Lestari RP, Fardilla FP, Setyaningsih WAW, Arfian N, et al. The impact of COL1A1 and COL6A1 expression on hypospadias and penile curvature severity. BMC Urol. 2020;20(1):189.
19. Wang D, Zhang M, Guan H, Wang X. Osteogenesis Imperfecta Due to Combined Heterozygous Mutations in Both COL1A1 and COL1A2, Coexisting With Pituitary Stalk Interruption Syndrome. Front Endocrinol (Lausanne). 2019;10:193.
20. Perera EM, Martin H, Seeherunvong T, Kos L, Hughes IA, Hawkins JR, et al. Tescalcin, a novel gene encoding a putative EF-hand Ca(2+)-binding protein, Col9a3, and renin are expressed in the mouse testis during the early stages of gonadal differentiation. Endocrinology. 2001;142(1):455-63.

21. Beverdam A, Koopman P. Expression profiling of purified mouse gonadal somatic cells during the critical time window of sex determination reveals novel candidate genes for human sexual dysgenesis syndromes. Human Molecular Genetics. 2005;15(3):417-31.
22. Araya S, Kratschmar DV, Tsachaki M, Stücheli S, Beck KR, Odermatt A. DHRS7 (SDR34C1) - A new player in the regulation of androgen receptor function by inactivation of 5α-dihydrotestosterone? J Steroid Biochem Mol Biol. 2017;171:288-95.
23. Zemanová L, Kirubakaran P, Pato IH, Štambergová H, Vondrášek J. The identification of new substrates of human DHRS7 by molecular modeling and in vitro testing. Int J Biol Macromol. 2017;105(Pt 1):171-82.
24. Guo C, Sun Y, Guo C, MacDonald BT, Borer JG, Li X. Dkk1 in the peri-cloaca mesenchyme regulates formation of anorectal and genitourinary tracts. Dev Biol. 2014;385(1):41-51.
25. van de Putte R, Wijers CH, de Blaauw I, Feitz WF, Marcelis CL, Hakobjan M, et al. Sequencing of the DKK1 gene in patients with anorectal malformations and hypospadias. Eur J Pediatr. 2015;174(5):583-7.
26. McElreavey K, Jorgensen A, Eozenou C, Merel T, Bignon-Topalovic J, Tan DS, et al. Pathogenic variants in the DEAH-box RNA helicase DHX37 are a frequent cause of 46,XY gonadal dysgenesis and 46,XY testicular regression syndrome. Genet Med. 2020;22(1):150-9.
27. Zidoune H, Martinerie L, Tan DS, Askari M, Rezgoune D, Ladjouze A, et al. Expanding DSD Phenotypes Associated with Variants in the DEAH-Box RNA Helicase DHX37. Sex Dev. 2021;15(4):244-52.
28. de Oliveira FR, Mazzola TN, de Mello MP, Francese-Santos AP, Lemos-Marini SHV, Maciel-Guerra AT, et al. DHX37 and NR5A1 Variants Identified in Patients with 46,XY Partial Gonadal Dysgenesis. Life (Basel). 2023;13(5).
29. Raymond CS, Parker ED, Kettlewell JR, Brown LG, Page DC, Kusz K, et al. A region of human chromosome 9p required for testis development contains two genes related to known sexual regulators. Hum Mol Genet. 1999;8(6):989-96.
30. Kulkarni V, Chellasamy SK, Dhangar S, Ghatanatti J, Vundinti BR. Comprehensive molecular analysis identifies eight novel variants in XY females with disorders of sex development. Mol Hum Reprod. 2023;29(2).
31. Ounap K, Uibo O, Zordania R, Kiho L, Ilus T, Oiglane-Shlik E, et al. Three patients with 9p deletions including DMRT1 and DMRT2: a girl with XY complement, bilateral ovotestes, and extreme growth retardation, and two XX females with normal pubertal development. Am J Med Genet A. 2004;130a(4):415-23.
32. Camats N, Fernández-Cancio M, Audí L, Schaller A, Flück CE. Broad phenotypes in heterozygous NR5A1 46,XY patients with a disorder of sex development: an oligogenic origin? Eur J Hum Genet. 2018;26(9):1329-38.
33. Vinci G, Chantot-Bastaraud S, El Houate B, Lortat-Jacob S, Brauner R, McElreavey K. Association of deletion 9p, 46,XY gonadal dysgenesis and autistic spectrum disorder. Molecular Human Reproduction. 2007;13(9):685-9.
34. Ledig S, Hiort O, Scherer G, Hoffmann M, Wolff G, Morlot S, et al. Array-CGH analysis in patients with syndromic and non-syndromic XY gonadal dysgenesis: evaluation of array CGH as diagnostic tool and search for new candidate loci. Hum Reprod. 2010;25(10):2637-46.
35. de Souza BF. Analysis of Novel Steroidogenic Factor-1 Targets in the Human Adrenal Gland: University College London; 2011.
36. Hurley TM, McClive PJ, Sarraj MA, Sinclair AH. Eki2 is upregulated specifically in the testis during mouse sex determination. Gene Expr Patterns. 2004;4(2):135-40.
37. Gustin SE, Western PS, McClive PJ, Harley VR, Koopman PA, Sinclair AH. Testis development, fertility, and survival in Ethanolamine kinase 2-deficient mice. Endocrinology. 2008;149(12):6176-86.
38. Barseghyan H, Symon A, Zadikyan M, Almalvez M, Segura EE, Eskin A, et al. Identification of novel candidate genes for 46,XY disorders of sex development (DSD) using a C57BL/6J-Y (POS) mouse model. Biol Sex Differ. 2018;9(1):8.
39. 53rd Annual Meeting of the European Society for Paediatric Endocrinology (ESPE). Dublin, Ireland, September 18-20, 2014: Abstracts. Hormone Research in Paediatrics. 2014;82(Suppl. 1):1-508.

40. Imamichi Y, Mizutani T, Ju Y, Matsumura T, Kawabe S, Kanno M, et al. Transcriptional regulation of human ferredoxin reductase through an intronic enhancer in steroidogenic cells. Biochim Biophys Acta. 2014;1839(1):33-42.
41. Slone J, Peng Y, Chamberlin A, Harris B, Kaylor J, McDonald MT, et al. Biallelic mutations in FDXR cause neurodegeneration associated with inflammation. J Hum Genet. 2018;63(12):1211-22.
42. Upadhyay K, Loke J, O V, Taragin B, Ostrer H. Biallelic mutations in FLNB cause a skeletal dysplasia with 46,XY gonadal dysgenesis by activating β-catenin. Clin Genet. 2018;93(2):412-6.
43. Foresta C, Zuccarello D, Garolla A, Ferlin A. Role of hormones, genes, and environment in human cryptorchidism. Endocr Rev. 2008;29(5):560-80.
44. Hempel M, Casar Tena T, Diehl T, Burczyk MS, Strom TM, Kubisch C, et al. Compound heterozygous GATA5 mutations in a girl with hydrops fetalis, congenital heart defects and genital anomalies. Hum Genet. 2017;136(3):339-46.
45. Molkentin JD, Tymitz KM, Richardson JA, Olson EN. Abnormalities of the genitourinary tract in female mice lacking GATA5. Mol Cell Biol. 2000;20(14):5256-60.
46. Zidoune H, Ladjouze A, Chellat-Rezgoune D, Boukri A, Dib SA, Nouri N, et al. Novel Genomic Variants, Atypical Phenotypes and Evidence of a Digenic/Oligogenic Contribution to Disorders/Differences of Sex Development in a Large North African Cohort. Front Genet. 2022;13:900574.
47. França MM, Jorge AA, Carvalho LR, Costalonga EF, Vasques GA, Leite CC, et al. Novel heterozygous nonsense GLI2 mutations in patients with hypopituitarism and ectopic posterior pituitary lobe without holoprosencephaly. J Clin Endocrinol Metab. 2010;95(11):E384-91.
48. Carmichael SL, Ma C, Choudhry S, Lammer EJ, Witte JS, Shaw GM. Hypospadias and genes related to genital tubercle and early urethral development. J Urol. 2013;190(5):1884-92.
49. Kothandapani A, Lewis SR, Noel JL, Zacharski A, Krellwitz K, Baines A, et al. GLI3 resides at the intersection of hedgehog and androgen action to promote male sex differentiation. PLoS Genet. 2020;16(6):e1008810.
50. Brauner R, Bignon-Topalovic J, Bashamboo A, McElreavey K. Pituitary stalk interruption syndrome is characterized by genetic heterogeneity. PLoS One. 2020;15(12):e0242358.
51. Quaynor SD, Bosley ME, Duckworth CG, Porter KR, Kim SH, Kim HG, et al. Targeted next generation sequencing approach identifies eighteen new candidate genes in normosmic hypogonadotropic hypogonadism and Kallmann syndrome. Mol Cell Endocrinol. 2016;437:86-96.
52. Baz-Redón N, Soler-Colomer L, Fernández-Cancio M, Benito-Sanz S, Garrido M, Moliné T, et al. Novel variant in HHAT as a cause of different sex development with partial gonadal dysgenesis associated with microcephaly, eye defects, and distal phalangeal hypoplasia of both thumbs: Case report. Front Endocrinol (Lausanne). 2022;13:957969.
53. Mazen I, Kamel A, McElreavey K, Bashamboo A, Elaidy A, Abdel-Hamid MS. A Homozygous Missense Variant in Hedgehog Acyltransferase (HHAT) Gene Associated with 46,XY Gonadal Dysgenesis. Sex Dev. 2022;16(4):261-5.
54. Rjiba K, Mougou-Zerelli S, Hamida IH, Saad G, Khadija B, Jelloul A, et al. Additional evidence for the role of chromosomal imbalances and SOX8, ZNRF3 and HHAT gene variants in early human testis development. Reprod Biol Endocrinol. 2023;21(1):2.
55. Callier P, Calvel P, Matevossian A, Makrythanasis P, Bernard P, Kurosaka H, et al. Loss of function mutation in the palmitoyl-transferase HHAT leads to syndromic 46,XY disorder of sex development by impeding Hedgehog protein palmitoylation and signaling. PLoS Genet. 2014;10(5):e1004340.
56. Qiu Z, Elsayed Z, Peterkin V, Alkatib S, Bennett D, Landry JW. Ino80 is essential for proximal-distal axis asymmetry in part by regulating Bmp4 expression. BMC Biol. 2016;14:18.
57. Fan Y, Zhang X, Wang L, Wang R, Huang Z, Sun Y, et al. Diagnostic Application of Targeted Next-Generation Sequencing of 80 Genes Associated with Disorders of Sexual Development. Sci Rep. 2017;7:44536-.
58. Wang Y, Kakinuma N, Zhu Y, Kiyama R. Nucleo-cytoplasmic shuttling of human Kank protein accompanies intracellular translocation of beta-catenin. J Cell Sci. 2006;119(Pt 19):4002-10.
59. Maatouk DM, DiNapoli L, Alvers A, Parker KL, Taketo MM, Capel B. Stabilization of β-catenin in XY gonads causes male-to-female sex-reversal. Human Molecular Genetics. 2008;17(19):2949-55.

60. Tannour-Louet M, Han S, Corbett ST, Louet J-F, Yatsenko S, Meyers L, et al. Identification of De Novo Copy Number Variants Associated with Human Disorders of Sexual Development. PLOS ONE. 2010;5(10):e15392.
61. Davarnia B, Panahi M, Rahimi B, Anari H, Farajollahi R, Rodbaneh EA, et al. De novo KAT6B mutation causes Say-Barber-Biesecker-Young-Simpson variant of Ohdo syndrome in an Iranian boy: a case report. J Med Case Rep. 2024;18(1):4.
62. Campeau PM, Lu JT, Dawson BC, Fokkema IF, Robertson SP, Gibbs RA, et al. The KAT6B-related disorders genitopatellar syndrome and Ohdo/SBBYS syndrome have distinct clinical features reflecting distinct molecular mechanisms. Hum Mutat. 2012;33(11):1520-5.
63. de Lau W, Peng WC, Gros P, Clevers H. The R-spondin/Lgr5/Rnf43 module: regulator of Wnt signal strength. Genes Dev. 2014;28(4):305-16.
64. Arboleda VA, Sandberg DE, Vilain E. DSDs: genetics, underlying pathologies and psychosexual differentiation. Nat Rev Endocrinol. 2014;10(10):603-15.
65. He X, Semenov M, Tamai K, Zeng X. LDL receptor-related proteins 5 and 6 in Wnt/beta-catenin signaling: arrows point the way. Development. 2004;131(8):1663-77.
66. Warr N, Carre GA, Siggers P, Faleato JV, Brixey R, Pope M, et al. Gadd45γ and Map3k4 interactions regulate mouse testis determination via p38 MAPK-mediated control of Sry expression. Dev Cell. 2012;23(5):1020-31.
67. Warr N, Siggers P, Carré GA, Wells S, Greenfield A. Genetic Analyses Reveal Functions for MAP2K3 and MAP2K6 in Mouse Testis Determination. Biol Reprod. 2016;94(5):103.
68. An N, Peng J, He G, Fan X, Li F, Chen H. Involvement of Activation of Mitogen-Activated Protein Kinase (MAPK)/Extracellular Signal-Regulated Kinase (ERK) Signaling Pathway in Proliferation of Urethral Plate Fibroblasts in Finasteride-Induced Rat Hypospadias. Med Sci Monit. 2018;24:8984-92.
69. Stone DL, Slavotinek A, Bouffard GG, Banerjee-Basu S, Baxevanis AD, Barr M, et al. Mutation of a gene encoding a putative chaperonin causes McKusick-Kaufman syndrome. Nat Genet. 2000;25(1):79-82.
70. Baxter RM, Vilain E. Translational genetics for diagnosis of human disorders of sex development. Annual review of genomics and human genetics. 2013;14:371-92.
71. Waleed MS, Varughese AA, Amba V, Pathalapati R. Bardet-Biedl Syndrome With Renal, Cardiac, and Genitourinary Malformations: A Case Report. Cureus. 2021;13(12):e20577.
72. Jameson SA, Natarajan A, Cool J, DeFalco T, Maatouk DM, Mork L, et al. Temporal transcriptional profiling of somatic and germ cells reveals biased lineage priming of sexual fate in the fetal mouse gonad. PLoS Genet. 2012;8(3):e1002575.
73. Li Y, Zheng M, Lau YF. The sex-determining factors SRY and SOX9 regulate similar target genes and promote testis cord formation during testicular differentiation. Cell Rep. 2014;8(3):723-33.
74. Li L, Gao F, Fan L, Su C, Liang X, Gong C. Disorders of Sex Development in Individuals Harbouring MAMLD1 Variants: WES and Interactome Evidence of Oligogenic Inheritance. Front Endocrinol (Lausanne). 2020;11:582516.
75. Martinez de Lapiscina I, Kouri C, Aurrekoetxea J, Sanchez M, Naamneh Elzenaty R, Sauter KS, et al. Genetic reanalysis of patients with a difference of sex development carrying the NR5A1/SF-1 variant p.Gly146Ala has discovered other likely disease-causing variations. PLoS One. 2023;18(7):e0287515.
76. Chachlaki K, Messina A, Delli V, Leysen V, Maurnyi C, Huber C, et al. NOS1 mutations cause hypogonadotropic hypogonadism with sensory and cognitive deficits that can be reversed in infantile mice. Sci Transl Med. 2022;14(665):eabh2369.
77. Nilsson M, Stulnig TM, Lin CY, Yeo AL, Nowotny P, Liu ET, et al. Liver X receptors regulate adrenal steroidogenesis and hypothalamic-pituitary-adrenal feedback. Mol Endocrinol. 2007;21(1):126-37.
78. Jarvis S, Williamson C, Bevan CL. Liver X Receptors and Male (In)fertility. Int J Mol Sci. 2019;20(21).
79. Rondanino C, Ouchchane L, Chauffour C, Marceau G, Déchelotte P, Sion B, et al. Levels of liver X receptors in testicular biopsies of patients with azoospermia. Fertil Steril. 2014;102(2):361-71.e5.

80. Maqdasy S, El Hajjaji FZ, Baptissart M, Viennois E, Oumeddour A, Brugnon F, et al. Identification of the Functions of Liver X Receptor-β in Sertoli Cells Using a Targeted Expression-Rescue Model. Endocrinology. 2015;156(12):4545-57.
81. Robertson KM, Schuster GU, Steffensen KR, Hovatta O, Meaney S, Hultenby K, et al. The liver X receptor-{beta} is essential for maintaining cholesterol homeostasis in the testis. Endocrinology. 2005;146(6):2519-30.
82. Brennan J, Tilmann C, Capel B. Pdgfr-alpha mediates testis cord organization and fetal Leydig cell development in the XY gonad. Genes Dev. 2003;17(6):800-10.
83. Qian C, Wu Z, Ng RC, Garcia-Barceló MM, Yuan ZW, Wong KKY, et al. Conditional deletion of platelet derived growth factor receptor alpha (Pdgfra) in urorectal mesenchyme causes mesenchyme apoptosis and urorectal developmental anomalies in mice. Cell Death Differ. 2019;26(8):1396-410.
84. Tsai MC, Weng YH, Lin YF, Wang YC, Yu HW, Chou YY, et al. Whole-Exome Sequencing Identified Rare Genetic Variants Associated with Undervirilized Genitalia in Taiwanese Pediatric Patients. Biomedicines. 2023;11(2).
85. Nie X, Arend LJ. Pkd1 is required for male reproductive tract development. Mech Dev. 2013;130(11-12):567-76.
86. Chen Y, Sun T, Niu Y, Wang D, Xiong Z, Li C, et al. Correlations Among Genotype and Outcome in Chinese Male Patients With Congenital Hypogonadotropic Hypogonadism Under HCG Treatment. J Sex Med. 2020;17(4):645-57.
87. Richard MA, Sok P, Canon S, Brown AL, Peckham-Gregory EC, Nembhard WN, et al. The role of genetic variation in DGKK on moderate and severe hypospadias. Birth Defects Res. 2019;111(13):932-7.
88. Barraud S, Delemer B, Poirsier-Violle C, Bouligand J, Mérol JC, Grange F, et al. Congenital Hypogonadotropic Hypogonadism with Anosmia and Gorlin Features Caused by a PTCH1 Mutation Reveals a New Candidate Gene for Kallmann Syndrome. Neuroendocrinology. 2021;111(1-2):99-114.
89. Saraç M, Canpolat Ş, Önalan Etem E, Tektemur A, Tartar T, Bakal U, et al. The role of sonic hedgehog homologue signal pathway in hypospadias aetiology. J Pediatr Urol. 2021;17(5):630.e1-.e7.
90. Ayers K, Kumar R, Robevska G, Bruell S, Bell K, Malik MA, et al. Familial bilateral cryptorchidism is caused by recessive variants in RXFP2. J Med Genet. 2019;56(11):727-33.
91. Dicke AK, Albrethsen J, Hoare BL, Wyrwoll MJ, Busch AS, Fietz D, et al. Bi-allelic variants in INSL3 and RXFP2 cause bilateral cryptorchidism and male infertility. Hum Reprod. 2023;38(7):1412-23.
92. Giacobini P. Shaping the Reproductive System: Role of Semaphorins in Gonadotropin-Releasing Hormone Development and Function. Neuroendocrinology. 2015;102(3):200-15.
93. Kotan LD, Ternier G, Cakir AD, Emeksiz HC, Turan I, Delpouve G, et al. Loss-of-function variants in SEMA3F and PLXNA3 encoding semaphorin-3F and its receptor plexin-A3 respectively cause idiopathic hypogonadotropic hypogonadism. Genet Med. 2021;23(6):1008-16.
94. Jaillard S, Akloul L, Beaumont M, Hamdi-Roze H, Dubourg C, Odent S, et al. Array-CGH diagnosis in ovarian failure: identification of new molecular actors for ovarian physiology. J Ovarian Res. 2016;9(1):63.
95. Warr N, Siggers P, Bogani D, Brixey R, Pastorelli L, Yates L, et al. Sfrp1 and Sfrp2 are required for normal male sexual development in mice. Dev Biol. 2009;326(2):273-84.
96. Kawano Y, Diez S, Uysal-Onganer P, Darrington RS, Waxman J, Kypta RM. Secreted Frizzled-related protein-1 is a negative regulator of androgen receptor activity in prostate cancer. Br J Cancer. 2009;100(7):1165-74.
97. Miraoui H, Dwyer AA, Sykiotis GP, Plummer L, Chung W, Feng B, et al. Mutations in FGF17, IL17RD, DUSP6, SPRY4, and FLRT3 are identified in individuals with congenital hypogonadotropic hypogonadism. Am J Hum Genet. 2013;92(5):725-43.
98. Men M, Wang X, Wu J, Zeng W, Jiang F, Zheng R, et al. Prevalence and associated phenotypes of DUSP6, IL17RD and SPRY4 variants in a large Chinese cohort with isolated hypogonadotropic hypogonadism. J Med Genet. 2021;58(1):66-72.
99. Indirli R, Cangiano B, Profka E, Mantovani G, Persani L, Arosio M, et al. A Rare SPRY4 Gene Mutation Is Associated With Anosmia and Adult-Onset Isolated Hypogonadotropic Hypogonadism. Front Endocrinol (Lausanne). 2019;10:781.

100. Gach A, Pinkier I, Wysocka U, Sałacińska K, Salachna D, Szarras-Czapnik M, et al. New findings in oligogenic inheritance of congenital hypogonadotropic hypogonadism. Arch Med Sci. 2022;18(2):353-64.
101. Xu B, Yang WH, Gerin I, Hu CD, Hammer GD, Koenig RJ. Dax-1 and steroid receptor RNA activator (SRA) function as transcriptional coactivators for steroidogenic factor 1 in steroidogenesis. Mol Cell Biol. 2009;29(7):1719-34.
102. Fabbri-Scallet H, Werner R, Guaragna MS, de Andrade JGR, Maciel-Guerra AT, Hornig NC, et al. Can Non-Coding NR5A1 Gene Variants Explain Phenotypes of Disorders of Sex Development? Sex Dev. 2022;16(4):252-60.
103. Kotan LD, Cooper C, Darcan Ş, Carr IM, Özen S, Yan Y, et al. Idiopathic Hypogonadotropic Hypogonadism Caused by Inactivating Mutations in SRA1. J Clin Res Pediatr Endocrinol. 2016;8(2):125-34.
104. Globa E, Zelinska N, Shcherbak Y, Bignon-Topalovic J, Bashamboo A, McElreavey K. Disorders of Sex Development in a Large Ukrainian Cohort: Clinical Diversity and Genetic Findings. Front Endocrinol (Lausanne). 2022;13:810782.
105. Zlotina A, Kiselev A, Sergushichev A, Parmon E, Kostareva A. Rare Case of Ulnar-Mammary-Like Syndrome With Left Ventricular Tachycardia and Lack of TBX3 Mutation. Front Genet. 2018;9:209.
106. Zhang X, Chen L, Li L, An J, He Q, Zhang X, et al. Literature review, report, and analysis of genotype and clinical phenotype of a rare case of ulnar-mammary syndrome. Front Pediatr. 2023;11:1052931.
107. Sanjad SA, Sakati NA, Abu-Osba YK, Kaddoura R, Milner RD. A new syndrome of congenital hypoparathyroidism, severe growth failure, and dysmorphic features. Archives of Disease in Childhood. 1991;66(2):193-6.
108. Padidela R, Kelberman D, Press M, Al-Khawari M, Hindmarsh PC, Dattani MT. Mutation in the TBCE Gene Is Associated with Hypoparathyroidism-Retardation-Dysmorphism Syndrome Featuring Pituitary Hormone Deficiencies and Hypoplasia of the Anterior Pituitary and the Corpus Callosum. The Journal of Clinical Endocrinology & Metabolism. 2009;94(8):2686-91.
109. Douglas NC, Heng K, Sauer MV, Papaioannou VE. Dynamic expression of Tbx2 subfamily genes in development of the mouse reproductive system. Dev Dyn. 2012;241(2):365-75.
110. Werner R, Mönig I, Lünstedt R, Wünsch L, Thorns C, Reiz B, et al. New NR5A1 mutations and phenotypic variations of gonadal dysgenesis. PloS one. 2017;12(5):e0176720-e.
111. Bashamboo A, Brauner R, Bignon-Topalovic J, Lortat-Jacob S, Karageorgou V, Lourenco D, et al. Mutations in the FOG2/ZFPM2 gene are associated with anomalies of human testis determination. Human Molecular Genetics. 2014;23(14):3657-65.
112. Bastian C, Muller JB, Lortat-Jacob S, Nihoul-Fékété C, Bignon-Topalovic J, McElreavey K, et al. Genetic mutations and somatic anomalies in association with 46,XY gonadal dysgenesis. Fertil Steril. 2015;103(5):1297-304.
113. Zhou Y, Liu J, Wu S, Li W, Zheng Y. Case report: A heterozygous mutation in ZNF462 leads to growth hormone deficiency. Front Genet. 2022;13:1015021.
114. Pellino G, Chiasso L, Fiori G, Mazzone S, Zama D, Cordelli DM, et al. Acute lymphoblastic leukemia in a child with Weiss-Kruszka syndrome: Casual or causal association? Eur J Med Genet. 2022;65(4):104457.
115. Iivonen AP, Kärkinen J, Yellapragada V, Sidoroff V, Almusa H, Vaaralahti K, et al. Kallmann syndrome in a patient with Weiss-Kruszka syndrome and a de novo deletion in 9q31.2. Eur J Endocrinol. 2021;185(1):57-66.
116. Renaux A, Papadimitriou S, Versbraegen N, Nachtegael C, Boutry S, Nowé A, et al. ORVAL: a novel platform for the prediction and exploration of disease-causing oligogenic variant combinations. Nucleic Acids Res. 2019;47(W1):W93-w8.
117. Rossetti S, Chauveau D, Kubly V, Slezak JM, Saggar-Malik AK, Pei Y, et al. Association of mutation position in polycystic kidney disease 1 (PKD1) gene and development of a vascular phenotype. Lancet. 2003;361(9376):2196-201.
118. Bleyl SB, Saijoh Y, Bax NA, Gittenberger-de Groot AC, Wisse LJ, Chapman SC, et al. Dysregulation of the PDGFRA gene causes inflow tract anomalies including TAPVR: integrating evidence from human genetics and model organisms. Hum Mol Genet. 2010;19(7):1286-301.

119. Longoni M, Russell MK, High FA, Darvishi K, Maalouf FI, Kashani A, et al. Prevalence and penetrance of ZFPM2 mutations and deletions causing congenital diaphragmatic hernia. Clin Genet. 2015;87(4):362-7.
120. Geller F, Feenstra B, Carstensen L, Pers TH, van Rooij IA, Körberg IB, et al. Genome-wide association analyses identify variants in developmental genes associated with hypospadias. Nat Genet. 2014;46(9):957-63.
121. Radhakrishna U, Bornholdt D, Scott HS, Patel UC, Rossier C, Engel H, et al. The phenotypic spectrum of GLI3 morphopathies includes autosomal dominant preaxial polydactyly type-IV and postaxial polydactyly type-A/B; No phenotype prediction from the position of GLI3 mutations. Am J Hum Genet. 1999;65(3):645-55.
122. Zhou XL, Eriksson U, Werelius B, Kressner U, Sun XF, Lindblom A. Definition of candidate low risk APC alleles in a Swedish population. Int J Cancer. 2004;110(4):550-7.
123. Edrees BM, Athar M, Al-Allaf FA, Taher MM, Khan W, Bouazzaoui A, et al. Next-generation sequencing for molecular diagnosis of autosomal recessive polycystic kidney disease. Gene. 2016;591(1):214-26.
124. Andres EM, Earnest KK, Xuan H, Zhong C, Rice ML, Raza MH. Innovative Family-Based Genetically Informed Series of Analyses of Whole-Exome Data Supports Likely Inheritance for Grammar in Children with Specific Language Impairment. Children (Basel). 2023;10(7).
125. Kim H, Park HC, Ryu H, Kim H, Lee HS, Heo J, et al. Genetic Characteristics of Korean Patients with Autosomal Dominant Polycystic Kidney Disease by Targeted Exome Sequencing. Sci Rep. 2019;9(1):16952.
126. Martínez-Gil N, Roca-Ayats N, Atalay N, Pineda-Moncusí M, Garcia-Giralt N, Van Hul W, et al. Functional Assessment of Coding and Regulatory Variants From the DKK1 Locus. JBMR Plus. 2020;4(12):e10423.
127. Mazzoni SM, Fearon ER. AXIN1 and AXIN2 variants in gastrointestinal cancers. Cancer Lett. 2014;355(1):1-8.
128. Bertolacini CD, Ribeiro-Bicudo LA, Petrin A, Richieri-Costa A, Murray JC. Clinical findings in patients with GLI2 mutations--phenotypic variability. Clin Genet. 2012;81(1):70-5.
129. Waterworth DM, Li L, Scott R, Warren L, Gillson C, Aponte J, et al. A low-frequency variant in MAPK14 provides mechanistic evidence of a link with myeloperoxidase: a prognostic cardiovascular risk marker. J Am Heart Assoc. 2014;3(4).
130. Yang G, Ullah HMA, Parker E, Gorsi B, Libowitz M, Maguire C, et al. Neurite outgrowth deficits caused by rare PLXNB1 mutation in pediatric bipolar disorder. Mol Psychiatry. 2023;28(6):2525-39.
131. Iwasa YI, Nishio SY, Usami SI. Comprehensive Genetic Analysis of Japanese Autosomal Dominant Sensorineural Hearing Loss Patients. PLoS One. 2016;11(12):e0166781.
132. Yang H, Zheng Z, Cai H, Li H, Ye X, Zhang X, et al. Three novel missense mutations in the filamin B gene are associated with isolated congenital talipes equinovarus. Hum Genet. 2016;135(10):1181-9.
133. Ouyang XM, Yan D, Du LL, Hejtmancik JF, Jacobson SG, Nance WE, et al. Characterization of Usher syndrome type I gene mutations in an Usher syndrome patient population. Hum Genet. 2005;116(4):292-9.
134. Bear KA, Solomon BD, Antonini S, Arnhold IJ, França MM, Gerkes EH, et al. Pathogenic mutations in GLI2 cause a specific phenotype that is distinct from holoprosencephaly. J Med Genet. 2014;51(6):413-8.
135. Rahimov F, Ribeiro LA, de Miranda E, Richieri-Costa A, Murray JC. GLI2 mutations in four Brazilian patients: how wide is the phenotypic spectrum? Am J Med Genet A. 2006;140(23):2571-6.
136. Férec C, Novelli G, Verlingue C, Quéré I, Dallapiccola B, Audrézet MP, et al. Identification of six novel CFTR mutations in a sample of Italian cystic fibrosis patients. Mol Cell Probes. 1995;9(2):135-7.
137. Castellví-Bel S, Sheikhavandi S, Telatar M, Tai LQ, Hwang M, Wang Z, et al. New mutations, polymorphisms, and rare variants in the ATM gene detected by a novel SSCP strategy. Hum Mutat. 1999;14(2):156-62.

138. Bogliolo M, Bluteau D, Lespinasse J, Pujol R, Vasquez N, d'Enghien CD, et al. Biallelic truncating FANCM mutations cause early-onset cancer but not Fanconi anemia. Genet Med. 2018;20(4):458-63.
139. de Breuk A, Lechanteur YTE, Astuti G, Galbany JC, Klaver CCW, Hoyng CB, et al. Common and rare variants in patients with early onset drusen maculopathy. Clin Genet. 2022;102(5):414-23.
140. Ratnapriya R, Zhan X, Fariss RN, Branham KE, Zipprer D, Chakarova CF, et al. Rare and common variants in extracellular matrix gene Fibrillin 2 (FBN2) are associated with macular degeneration. Hum Mol Genet. 2014;23(21):5827-37.
141. Peter M, Fawaz L, Drop SL, Visser HK, Sippell WG. Hereditary defect in biosynthesis of aldosterone: aldosterone synthase deficiency 1964-1997. J Clin Endocrinol Metab. 1997;82(11):3525-8.
142. Ng SB, Buckingham KJ, Lee C, Bigham AW, Tabor HK, Dent KM, et al. Exome sequencing identifies the cause of a mendelian disorder. Nat Genet. 2010;42(1):30-5.
143. Gorsic LK, Dapas M, Legro RS, Hayes MG, Urbanek M. Functional Genetic Variation in the Anti-Müllerian Hormone Pathway in Women With Polycystic Ovary Syndrome. J Clin Endocrinol Metab. 2019;104(7):2855-74.
144. Cella W, de Vasconcellos JP, de Melo MB, Kneipp B, Costa FF, Longui CA, et al. Structural assessment of PITX2, FOXC1, CYP1B1, and GJA1 genes in patients with Axenfeld-Rieger syndrome with developmental glaucoma. Invest Ophthalmol Vis Sci. 2006;47(5):1803-9.
145. Boutboul S, Black GC, Moore JE, Sinton J, Menasche M, Munier FL, et al. A subset of patients with epithelial basement membrane corneal dystrophy have mutations in TGFBI/BIGH3. Hum Mutat. 2006;27(6):553-7.
146. Kanipakam H, Sharma K, Thinlas T, Mohammad G, Pasha MAQ. Structural and functional alterations of nitric oxide synthase 3 due to missense variants associate with high-altitude pulmonary edema through dynamic study. J Biomol Struct Dyn. 2021;39(1):294-309.
147. Desai SS, Achrekar SK, Paranjape SR, Desai SK, Mangoli VS, Mahale SD. Association of allelic combinations of FSHR gene polymorphisms with ovarian response. Reprod Biomed Online. 2013;27(4):400-6.
148. Hsu JS, Zhang R, Yeung F, Tang CSM, Wong JKL, So MT, et al. Cancer gene mutations in congenital pulmonary airway malformation patients. ERJ Open Res. 2019;5(1).
149. Yang T, Wei X, Chai Y, Li L, Wu H. Genetic etiology study of the non-syndromic deafness in Chinese Hans by targeted next-generation sequencing. Orphanet J Rare Dis. 2013;8:85.
150. Kostopoulou E, Shah P, Ahmad N, Semple R, Hussain K. Gastrointestinal dysmotility and pancreatic insufficiency in 2 siblings with Donohue syndrome. Pediatr Diabetes. 2017;18(8):839-43.
151. Torrezan GT, de Almeida F, Figueiredo MCP, Barros BDF, de Paula CAA, Valieris R, et al. Complex Landscape of Germline Variants in Brazilian Patients With Hereditary and Early Onset Breast Cancer. Front Genet. 2018;9:161.
152. Patiño LC, Beau I, Carlosama C, Buitrago JC, González R, Suárez CF, et al. New mutations in non-syndromic primary ovarian insufficiency patients identified via whole-exome sequencing. Hum Reprod. 2017;32(7):1512-20.
153. Dörk T, Bendix R, Bremer M, Rades D, Klöpper K, Nicke M, et al. Spectrum of ATM gene mutations in a hospital-based series of unselected breast cancer patients. Cancer Res. 2001;61(20):7608-15.
154. Cox AJ, Grady F, Velez G, Mahajan VB, Ferguson PJ, Kitchen A, et al. In trans variant calling reveals enrichment for compound heterozygous variants in genes involved in neuronal development and growth. Genet Res (Camb). 2019;101:e8.
155. Ghani M, Lang AE, Zinman L, Nacmias B, Sorbi S, Bessi V, et al. Mutation analysis of patients with neurodegenerative disorders using NeuroX array. Neurobiol Aging. 2015;36(1):545.e9-14.
156. Jaouadi H, Theron A, Norscini G, Avierinos JF, Zaffran S. Genetic and phenotypic continuum of HOXA genes: A case with double HOXA9/HOXA13 mutations. Mol Med Rep. 2023;27(3).
157. Liu H, Chatel S, Simard C, Syam N, Salle L, Probst V, et al. Molecular genetics and functional anomalies in a series of 248 Brugada cases with 11 mutations in the TRPM4 channel. PLoS One. 2013;8(1):e54131.

158. Feuillan PP, Ng D, Han JC, Sapp JC, Wetsch K, Spaulding E, et al. Patients with Bardet-Biedl syndrome have hyperleptinemia suggestive of leptin resistance. J Clin Endocrinol Metab. 2011;96(3):E528-35.
159. Barbat B, Bogyo A, Raux-Demay MC, Kuttenn F, Boué J, Simon-Bouy B, et al. Screening of CYP21 gene mutations in 129 French patients affected by steroid 21-hydroxylase deficiency. Hum Mutat. 1995;5(2):126-30.
160. Salehi Z, Keramatipour M, Talebi S, Arab SS, Naser Moghadasi A, Sahraian MA, et al. Exome sequencing reveals novel rare variants in Iranian familial multiple sclerosis: The importance of POLD2 in the disease pathogenesis. Genomics. 2021;113(4):2645-55.
161. Van Mil SW, Milona A, Dixon PH, Mullenbach R, Geenes VL, Chambers J, et al. Functional variants of the central bile acid sensor FXR identified in intrahepatic cholestasis of pregnancy. Gastroenterology. 2007;133(2):507-16.
162. Aitman TJ, Cooper LD, Norsworthy PJ, Wahid FN, Gray JK, Curtis BR, et al. Malaria susceptibility and CD36 mutation. Nature. 2000;405(6790):1015-6.
163. Eiselt R, Domanski TL, Zibat A, Mueller R, Presecan-Siedel E, Hustert E, et al. Identification and functional characterization of eight CYP3A4 protein variants. Pharmacogenetics. 2001;11(5):447-58.
164. Donner I, Katainen R, Tanskanen T, Kaasinen E, Aavikko M, Ovaska K, et al. Candidate susceptibility variants for esophageal squamous cell carcinoma. Genes Chromosomes Cancer. 2017;56(6):453-9.
165. Abreu GM, Tarantino RM, da Fonseca ACP, Andrade J, de Souza RB, Soares C, et al. Identification of Variants Responsible for Monogenic Forms of Diabetes in Brazil. Front Endocrinol (Lausanne). 2022;13:827325.
166. Hood RL, Lines MA, Nikkel SM, Schwartzentruber J, Beaulieu C, Nowaczyk MJ, et al. Mutations in SRCAP, encoding SNF2-related CREBBP activator protein, cause Floating-Harbor syndrome. Am J Hum Genet. 2012;90(2):308-13.
167. Gallardo TD, John GB, Bradshaw K, Welt C, Reijo-Pera R, Vogt PH, et al. Sequence variation at the human FOXO3 locus: a study of premature ovarian failure and primary amenorrhea. Hum Reprod. 2008;23(1):216-21.
168. Angrist M, Bolk S, Halushka M, Lapchak PA, Chakravarti A. Germline mutations in glial cell line-derived neurotrophic factor (GDNF) and RET in a Hirschsprung disease patient. Nat Genet. 1996;14(3):341-4.
169. Yamakawa-Kobayashi K, Ishiguro H, Arinami T, Miyazaki R, Hamaguchi H. A Val227Ala polymorphism in the peroxisome proliferator activated receptor alpha (PPARalpha) gene is associated with variations in serum lipid levels. J Med Genet. 2002;39(3):189-91.
170. Liu Q, Hesson LB, Nunez AC, Packham D, Hawkins NJ, Ward RL, et al. Pathogenic germline MCM9 variants are rare in Australian Lynch-like syndrome patients. Cancer Genet. 2016;209(11):497-500.
171. Eriksen MB, Nielsen MF, Brusgaard K, Tan Q, Andersen MS, Glintborg D, et al. Genetic alterations within the DENND1A gene in patients with polycystic ovary syndrome (PCOS). PLoS One. 2013;8(9):e77186.
172. D'Alessandro LC, Al Turki S, Manickaraj AK, Manase D, Mulder BJ, Bergin L, et al. Exome sequencing identifies rare variants in multiple genes in atrioventricular septal defect. Genet Med. 2016;18(2):189-98.
